# Early protective effect of a (“pan”) coronavirus vaccine (PanCoVac) in Roborovski dwarf hamsters after single-low dose intranasal administration

**DOI:** 10.3389/fimmu.2023.1166765

**Published:** 2023-07-13

**Authors:** Mohammed O. Abdelaziz, Martin J. Raftery, Julian Weihs, Olivia Bielawski, Richard Edel, Julia Köppke, Daria Vladimirova, Julia M. Adler, Theresa Firsching, Anne Voß, Achim D. Gruber, Luca V. Hummel, Ivan Fernandez Munoz, Francesca Müller-Marquardt, Gerald Willimsky, Nooran S. Elleboudy, Jakob Trimpert, Günther Schönrich

**Affiliations:** ^1^ Institute of Virology, Charité – Universitätsmedizin Berlin, Corporate Member of Freie Universität Berlin and Humboldt-Universität zu Berlin, Berlin, Germany; ^2^ Berlin Institute of Health, Charité – Universitätsmedizin Berlin, Berlin, Germany; ^3^ Department of Hematology, Oncology and Tumor Immunology, Charité – Universitätsmedizin Berlin, Corporate Member of Freie Universität Berlin and Humboldt-Universität zu Berlin, Berlin, Germany; ^4^ Department of Pediatrics, Division of Gastroenterology, Charité – Universitätsmedizin Berlin, Corporate Member of Freie Universität Berlin and Humboldt-Universität zu Berlin, Berlin, Germany; ^5^ Institute of Virology, Freie Universität Berlin, Berlin, Germany; ^6^ Institute of Veterinary Pathology, Freie Universität Berlin, Berlin, Germany; ^7^ Institute of Immunology, Charité-Universitätsmedizin Berlin, Corporate Member of Freie Universität Berlin and Humboldt-Universität zu Berlin, Berlin, Germany; ^8^ German Cancer Research Center, Heidelberg, Germany; ^9^ German Cancer Consortium, Partner Site Berlin, Berlin, Germany; ^10^ Department of Microbiology and Immunology, Faculty of Pharmacy, Ain Shams University, Cairo, Egypt

**Keywords:** universal COVID-19 vaccine, coronaviruses, multi-epitope vaccine, T cell epitopes, pan-coronavirus vaccine, dwarf hamster COVID-19 model, T-cell-directed vaccine

## Abstract

**Introduction:**

The coronavirus disease 2019 (COVID-19) pandemic caused by severe acute respiratory syndrome coronavirus 2 (SARS-CoV-2) has highlighted the danger posed by human coronaviruses. Rapid emergence of immunoevasive variants and waning antiviral immunity decrease the effect of the currently available vaccines, which aim at induction of neutralizing antibodies. In contrast, T cells are marginally affected by antigen evolution although they represent the major mediators of virus control and vaccine protection against virus-induced disease.

**Materials and methods:**

We generated a multi-epitope vaccine (PanCoVac) that encodes the conserved T cell epitopes from all structural proteins of coronaviruses. PanCoVac contains elements that facilitate efficient processing and presentation of PanCoVac-encoded T cell epitopes and can be uploaded to any available vaccine platform. For proof of principle, we cloned PanCoVac into a non-integrating lentivirus vector (NILV-PanCoVac). We chose Roborovski dwarf hamsters for a first step in evaluating PanCoVac *in vivo*. Unlike mice, they are naturally susceptible to SARS-CoV-2 infection. Moreover, Roborovski dwarf hamsters develop COVID-19-like disease after infection with SARS-CoV-2 enabling us to look at pathology and clinical symptoms.

**Results:**

Using HLA-A^*^ 0201-restricted reporter T cells and U251 cells expressing a tagged version of PanCoVac, we confirmed *in vitro* that PanCoVac is processed and presented by HLA-A^*^ 0201. As mucosal immunity in the respiratory tract is crucial for protection against respiratory viruses such as SARS-CoV-2, we tested the protective effect of single-low dose of NILV-PanCoVac administered *via* the intranasal (i.n.) route in the Roborovski dwarf hamster model of COVID-19. After infection with ancestral SARS-CoV-2, animals immunized with a single-low dose of NILV-PanCoVac i.n. did not show symptoms and had significantly decreased viral loads in the lung tissue. This protective effect was observed in the early phase (2 days post infection) after challenge and was not dependent on neutralizing antibodies.

**Conclusion:**

PanCoVac, a multi-epitope vaccine covering conserved T cell epitopes from all structural proteins of coronaviruses, might protect from severe disease caused by SARS-CoV-2 variants and future pathogenic coronaviruses. The use of (HLA-) humanized animal models will allow for further efficacy studies of PanCoVac-based vaccines *in vivo*.

## 1 Introduction

The coronavirus disease 2019 (COVID-19) pandemic illustrates the great danger posed by coronaviruses. These enveloped viruses belong to the subfamily *Coronavirinae* from the family *Coronaviridae* (1). They can jump from bats *via* bridging hosts into humans thereby adapting to and spreading in human populations (2, 3). This happened three times in the past 20 years. Severe acute respiratory syndrome coronavirus (SARS-CoV)-1 emerged in 2002 (4) and Middle East respiratory syndrome coronavirus (MERS-CoV) was first detected in 2012 (5). They were responsible for separate viral epidemics with case fatality rates of up to 10% for SARS-CoV-1 (6) and 35% for MERS-CoV (7). The currently circulating pandemic SARS-CoV-2 emerged in 2019 and is causing huge detrimental socio-economic damage and millions of deaths (8) although it has a much lower case fatality rate in unvaccinated populations compared to SARS-CoV-1 and MERS-CoV (9). In South East Asia, numerous bat species are infected with coronaviruses belonging to the *Sarbecovirus* subgenus of the genus *Betacoronavirus* like SARS-CoV-1 and SARS-CoV-2 (10–13). In this region, significant levels of bat-to-human coronavirus spillover are observed suggesting that future outbreaks with sarbecoviruses are likely (14). Thus, universal coronavirus vaccines that provide a broad, robust, and durable protection are urgently needed (15–19).

The coronavirus genome consists of non-segmented, single-stranded, positive-sense RNA and is the largest known amongst RNA viruses (20). It encodes non-structural and structural proteins. The latter encompass the spike (S), envelope (E), membrane (M), and nucleocapsid (N) protein. A receptor-binding domain (RBD) located on the S protein interacts with host cell surface receptors thereby facilitating viral entry. Currently available SARS-CoV-2 vaccines are administered *via* intramuscular injection and aim at systemic induction of neutralizing antibodies, which mostly bind to the RBD thereby preventing virus infection (21). Although these first generation vaccines have mitigated the effects of the pandemic (22), major problems remain. Firstly, the levels of neutralizing antibodies quickly decrease after vaccination (23, 24). Secondly, intramuscular injection only weakly stimulates antiviral mucosal immunity in the respiratory tract, the site of viral entry (25). Thirdly, emerging viral variants of concern (VOC) such as B.1.1.7 (Alpha), B.1.351 (Beta), P.1 (Gamma), B.1.617.2 (Delta), and the recently identified B.1.1.529 (Omicron) with its numerous subvariants (notably BA.1, BA.2, BA.4 and BA.5) evade neutralizing antibodies due to mutations mainly within the RBD sequence (26–30). These disadvantages combined explain why the effectiveness of current vaccines is waning rapidly resulting in loss of protection from infection and possibly also from disease (31–33).

Besides neutralizing antibodies, T cells originating in the thymus fulfill essential antiviral functions (34). CD8+ T cells eliminate virus-infected cells thereby preventing viral cell-to-cell spread and CD4+ T cells optimize antibody production by B cells (35). In addition, CD4+ T cells provide signals that help to generate and program memory CD8+ T cells (36, 37). In non-severe SARS-CoV-2 infections of unvaccinated virus-naive individuals virus-specific T cell responses precede PCR detection and occur 1-2 weeks before virus-specific antibodies (38). T cells, either induced by infection, by vaccination or by their combination, protect from severe COVID-19 and are more important players than neutralizing antibodies in elimination of SARS-CoV-2 (15, 39–43). For example, patients deficient in B cells but with intact T cell function can cope with SARS-CoV-2 infection (44–46). In macaques that had recovered from SARS-CoV-2 infection, depletion of CD8+ T cells decreases the protective effect of acquired immunity against re-challenge (47). In line with these observations, a SARS-CoV-2 N protein-based vaccine, which does not elicit neutralizing antibodies, established protective immunity in small animal models of COVID-19 (48).

The T cell responses against SARS-CoV-2 persist most likely for many years and are detectable even in the absence of memory B cell responses (49–57). In contrast to neutralizing antibodies that bind to the RBD, T cell responses are directed against a broad spectrum of epitopes and are not disrupted by the antigenic evolution of SARS-CoV-2 (58–70). This is explained by the polymorphic HLA molecules, which present a highly diverse repertoire of T cell epitopes derived from all viral proteins thereby preventing efficient viral immune escape (71).

Intriguingly, pre-existing T cell responses to SARS-CoV-2 epitopes are found frequently in unexposed individuals and pre-pandemic blood samples (51, 72–77). They are best explained by previous exposure to the four known endemic coronaviruses (HCoV-OC43, HCoV-229E, HCoV-HKU1, and HCoV-NL63) that cause about one-third of common colds in humans (78–80). There is accumulating evidence that these cross-reactive T cells are functional *in vivo* and have a positive effect on COVID-19 outcome and COVID-19 vaccination (41, 49, 55, 75, 81–88). It has been reported that pre-existing cross-reactive memory T cells predict efficient COVID-19 vaccine-induced immune responses (82–84). In addition, T cell epitopes have been identified that are highly conserved between human and animal coronaviruses (89) and bind to common human MHC molecules (90). Thus, induction of a broad and durable cross-reactive T cell response specific for highly conserved epitopes of pathogenic coronaviruses in the upper respiratory tract is an attractive strategy for urgently needed pan-coronavirus vaccines (91).

In this study, we generated a codon optimized DNA sequence (PanCoVac) that encodes in a compact form the conserved T cell epitopes from all structural proteins. For this purpose, we deconstructed coronavirus genomes and generated a multi-epitope vaccine with a special architecture facilitating processing and presentation of epitopes. We cloned PanCoVac into a non-integrating lentivirus vector (NILV-PanCoVac) and tested the protective effect of intranasal (i.n.) administration of a single low dose of NILV-PanCoVac in the Roborovski dwarf hamster model of COVID-19.

## 2 Materials and methods

### 2.1 *In silico* identification of epitopes

For PanCoVac design, NetMHCpan-4.1 in combination with data available in the Immune Epitope Database (IEDB, http://www.iedb.org/) were used to identify peptides potentially binding to human MHC-I (HLA-I) alleles (HLA-A, HLA-B, and HLA-C). NetMHCIIpan 4.0 was used for bioinformatic analysis of peptide binding to human MHC-II (HLA-II) alleles (92). Conserved regions of at least 8 amino acids from the structural proteins of SARS-CoV-1 (Tor 2), ancestral SARS-CoV-2 (Wuhan-Hu-1), SARS-CoV-2 variants (B.1.1.7, Alpha; B.1.351, Beta; P.1, Gamma), and common cold coronaviruses (HKU1, 229E, NL63 and OC43) were considered using the commonly applied half-maximal inhibitory concentration (IC_50_) threshold of 500 nM for HLA-I and 1000 nM for HLA-II.

### 2.2 *In silico* testing of antigenicity, allergenicity, and toxicity

For predicting antigenicity of PanCoVac, we used VaxiJen, the web server for alignment independent prediction of protective antigens (http://www.ddgpharmfac.net/vaxijen/VaxiJen/VaxiJen.html). Prediction is based on auto- and cross-covariance (ACC) transformation method. The threshold was adjusted to 0.5, the recommended threshold for maximal accuracy (93). The web server AllerCatPro 2.0 (https://allercatpro.bii.a-star.edu.sg/) was used for predicting allergenic potential (94). We analyzed peptide toxicity using the web server ToxinPred (https://webs.iiitd.edu.in/raghava/toxinpred/pep_test.php). This tool was adjusted to screen all PanCoVac peptides at fragment length of 20 amino acids. We applied a hybrid approach that combines support vector machine (SVM) output, at a threshold of 1.0, with motif information for a biologically reliable prediction of toxic peptides (95).

### 2.3 Codon optimization

PanCoVac amino acid sequence was reverse translated and the DNA codon usage was optimized for human cell expression using the Codon Optimization tool from Integrated DNA Technologies (IDT) (www.idtdna.com). The final PanCoVac DNA sequence was synthesized by Thermo-Fisher Scientific and cloned into pLeGo-iG2 (96). LeGO-iG2 was a gift from Boris Fehse (Addgene plasmid # 27341; http://n2t.net/addgene:27341; RRID: Addgene_27341). The PanCoVac sequences were inserted at the BamHI and NotI multiple-cloning site, followed by an internal ribosome entry site (IRES), which drives expression of enhanced green fluorescence protein (EGFP).

### 2.4 Detection of PanCoVac protein

We generated a FLAG-tagged version of PanCoVacE6 (see 2.9) by fusing the FLAG peptide to the C-terminus of PanCoVacE6 (PanCoVacE6-FLAG). U251 cells were left untransfected or transfected with PanCoVacE6-FLAG mRNA using lipofectamineTM MessengerMaxTM (Thermo-Fisher Scientific) following the manufacturer´s instructions. After 24 h, the medium was removed and the cells were washed two times with PBS and subsequently lysed with M-PER™ Mammalian protein extraction reagent (Thermo-Fisher Scientific). Immunoblotting of PanCoVacE6-FLAG and glyceraldehyde-3-phosphate dehydrogenase (GAPDH) encoded by a housekeeping gene was performed using anti-FLAG Antibody (FG4R) and anti-GAPDH antibody (1A10A10), respectively (both from Thermo-Fisher Scientific). The membrane was visualized using SuperSignal™ West Pico PLUS chemiluminescent substrate (Thermo-Fisher Scientific).

### 2.5 Cells and media

The glioblastoma cell line U251, which expresses HLA-A^*^ 0201, was a kind gift of L. Wiebusch (The Children’s Hospital, Laboratory for Molecular Biology, Charité-Universitätsmedizin Berlin, Berlin, Germany). Human Embryonic Kidney (HEK)-293T cells were purchased from Sigma-Aldrich. HEK-293 T and U251 cells were cultured in Dulbecco’s Modified Eagle Medium (DMEM) (Gibco™) supplemented with 1 mM sodium pyruvate (Gibco), 50 μg/ml gentamicin (Sigma-Aldrich), and 10% heat inactivated FBS (hiFBS) (HyClone™). Jurkat cells were cultured in RPMI 1640 medium (Gibco) supplemented with 2 mM L-glutamine (Gibco), 25 mM HEPES Buffer (Gibco), 50 μg/ml gentamicin, and 10% heat inactivated fetal bovine serum (hiFBS). Vero E6 cells (ATCC CRL-1586) were cultured in DMEM supplemented with 5% fetal bovine serum (PAN Biotech) as well as 100 IU/ml penicillin G and 100 µg/ml streptomycin (Corning).

### 2.6 Production and titration of lentivirus particles

Non-integrating lentiviral vector (NILV) particles were produced using HEK-293 T cells, as previously described (97, 98). Briefly, HEK-293T cells were transiently co-transfected with pLeGo-iG2-PanCoVac or empty vector, pMD2.G expressing the envelope glycoprotein of vesicular stomatitis virus Indiana (VSV-G), and encapsidation plasmid pD64V by using Lipofectamine™ 3000 (Thermo-Fisher Scientific). The plasmid pMD2.G (Addgene plasmid # 12259; http://n2t.net/addgene:12259; RRID: Addgene_12259) was a gift from Didier Trono (Laboratory for Virology and Genetics, School of Life Sciences, École Polytechnique Fédérale de Lausanne, Switzerland). For production of integrating lentiviral vector (LV) particles, pMDLg/pRRE (99) and pRSV-Rev (99) were used as packaging plasmids. Both, pMDLg/pRRE (Addgene plasmid # 12251; http://n2t.net/addgene:12251; RRID: Addgene_12251) and pRSV-Rev (Addgene plasmid # 12253; http://n2t.net/addgene:12253; RRID : Addgene_12253) were also gifts from Didier Trono.

At 24 h after transfection, the medium was changed. The supernatants were harvested at 48 h after transfection and cell debris was removed by 10 min centrifugation at 600 × g at 4°C. The virus particles were concentrated by ultracentrifugation at 30,000 rpm for 90 min at 4°C in thinwall polypropylene tubes (Beckmann Coulter) containing a 2 ml layer of 20% sucrose in PBS at the bottom. Lentivirus particles were resuspended in PBS, aliquoted and stored in -80°C until further use. Lentivirus vector copies were quantified by RT-qPCR as previously described (100) using SYBR Green and EGFP specific primers (EGFP_F: CACATGAAGCAGCACGACTT and EGFP_R: TGCTCAGGTAGTGGTTGTCG).

### 2.7 Transduction of U251 cells

Transduction of U251 cells with lentivirus particles was carried out as previously described (101). Briefly, the U251 cell suspensions (1 × 10^6^ cells in 1 ml of DMEM) were transduced with concentrated viral particles at a multiplicity of infection (MOI) of 2 in the presence of 8 μg/ml polybrene (Sigma-Aldrich). The cells were incubated at 37°C for 1 h, then spinoculated by centrifugation for 90 min at 600 × g at room temperature followed by seeding in 1.5 ml of fresh media in 6-well cell culture plates. Transduced cells were expanded and passaged in supplemented DMEM. The maximum number of passages before being included in assays was 2 for NILV- and 15 for LV-transduced cells.

### 2.8 *In vitro* mRNA transcription and mRNA transfection

First, we amplified the sequences of interest by PCR. The primer sequences for EGFP were: TAATACGACTCACTATAGATGGTGAGCAAGGGCGAGGAGC (forward primer) and TTACTTGTACAGCTCGTCCATG (reverse primer). PanCoVac was amplified with GCTAATACGACTCACTATAGGGACAGGCCACCATGGACTGGACCTGGATCCT as forward and TCATTTCTTTTTTTTGTCCTTTTTAGGCT as reverse primers, respectively. Then, *in vitro*-transcribed (IVT) mRNA of EGFP and PanCoVac were synthesized by using the HiScribe™ T7 ARCA mRNA Kit with tailing (New England Biolabs). Pseudo-UTP and 5-Methyl-CTP (Jena Bioscience) were used as modified nucleotides for mRNA synthesis. The synthesized mRNA was purified by the Monarch^®^RNA Cleanup Kit (New England Biolabs), aliquoted and stored at -80°C until further use. The mRNA purity and concentrations were analyzed using Nanodrop™ spectrophotometer (Thermo-Fisher Scientific). The mRNA transfection into U251 cells was carried out using lipofectamine™ MessengerMax™ (Thermo-Fisher Scientific) following the manufacturer´s instructions.

### 2.9 Reporter T cell assays

The reporter T cell assay was carried out as described previously (101). We used Jurkat cells expressing a HLA-A^*^ 0201-restricted TCR recognizing the epitope E6_29−38_ (TIHDIILECV) derived from the E6 protein of human papillomavirus (HPV) type 16 (102). These cells also express an EGFP reporter driven by activation of nuclear factor kappa-light-chain-enhancer of activated B-cells (NF-κB) (103). In order to investigate processing and presentation, the HPV 16 E6_29−38_ epitope was inserted in the middle of the S module of PanCoVac resulting in PanCoVacE6. To address the influence of the furin cleavage sites on processing and presentation of PanCovacE6, we generated a version of PanCoVacE6 lacking all the furin cleavage sites (PanCoVacE6Δfurin). The reporter T cells were stimulated with HLA-A^*^ 0201 expressing U251 cells, which had been transduced with integrating lentiviral vector (LV) or NILV encoding PanCoVacE6 (LV-PanCoVacE6 and NILV-PanCoVacE6, respectively). Moreover, we transfected U251 cells with 100 ng of *in vitro*-transcribed PanCoVacE6 mRNA or PanCoVacE6Δfurin. As a negative control, the cells were transduced with lentiviral vector encoding untagged PanCoVac (LV-PanCoVac) or transfected with 100 ng of EGFP mRNA. U251 cells pulsed with 1 μg/ml of HPV E6_29−38_ peptide were used as a positive control. The transduced or transfected U251 cells as well as positive and negative control cells were seeded in 96-well plates and incubated for 18 h. Afterwards, the reporter cells were added at a reporter cell to antigen-presenting cell (APC) ratio of 2:1. Co-culture was done for 24 h, then cells were removed and reporter cells were stained with Brilliant Violet 711™ anti-human CD3ϵ antibody (BioLegend) and the viability dye Zombie Violet™ (BioLegend). Stimulation of the reporter T cells with HPV E6-peptide bound to HLA-A^*^ 0201 was analyzed by detection of NF-κB driven EGFP fluorescence using FACS.

### 2.10 Roborovski dwarf hamster model and vaccination

The COVID-19 model based on Roborovski dwarf hamster (*P. roborovskii*) has been described previously (104). All animal procedures were performed according to the European Guidelines for Animal Studies after approval by the Institutional Animal Care Committee and the relevant state authority (Landesamt für Gesundheit und Soziales, Berlin, Permit number 0086/20). We obtained male and female Roborovski dwarf hamsters of 5 to 7 weeks of age from the German pet trade. Animals were housed in groups of 3–6 hamsters in GR-900 IVC cages (Tecniplast, Buguggiate) and provided with bountiful enrichment and nesting materials (Carfil, Oud-Turnhout). We randomly distributed animals into two groups; the test group (9 animals) was immunized with NILV-PanCoVac whereas the control group (9 animals) was immunized with empty vector (NILV) (**Supplementary Table 1**) shows number, sex distribution, and analysis date of experimental animals). All experimental animals were individually marked with a subcutaneously implanted IPTT-300 transponder (BMDS, Seaford) that facilitates remote identification and measurement of body temperature. The hamsters were inoculated i.n. with 30 μl PBS containing 1×10^5^ NILV-PanCoVac particles or NILV. After 21 days, the hamsters were challenged with a sub-lethal dose (1×10^4^ pfu) of the ancestral SARS-CoV-2 (BetaCoV/Germany/BavPat1/2020) strain in 30 μl cell culture medium. SARS-CoV-2 infection was performed i.n. as previously described (104).

RNA was extracted from oropharyngeal swabs and 50 mg lung tissue using the innuPREP Virus DNA/RNA Kit (Analytic Jena) according to the manufacturer’s instructions. Genomic virus RNA was quantified using a one-step RT–qPCR reaction with the NEB Luna Universal Probe One-Step RT–qPCR (New England Biolabs) and the 2019-nCoV RT–qPCR primers and probe (E_Sarbeco) on a qTOWER³ Real-Time PCR System (Analytik Jena), as previously described (104, 105). To obtain virus titers, duplicate ten-fold serial dilutions of lung tissue homogenates were plated on Vero E6 monolayers for 2 h at 37 °C. Afterwards, cells were washed with PBS and overlaid with semi-solid cell culture medium containing 1.5% microcrystalline cellulose (Vivapur MCG 611P, JRS Pharma) and incubated for 48 h at 37 °C. Plates were then fixed with 4% formalin and stained with 0.75% crystal violet for plaque counting

### 2.11 Lung histopathology

Samples from the lung tissue of hamsters were fixed with formalin, embedded in paraffin and analyzed as described previously (106). Briefly, paraffin sections of 2 μm thickness were prepared and stained with hematoxylin and eosin (HE). Microscopic changes were qualitatively described and scored according to standardized reporting criteria using a four-scale severity grading system (0: no lesions, 1: mild, 2: moderate, and 3: severe).

### 2.12 Serum neutralization tests

The capacity of sera obtained from dwarf hamsters after SARS-CoV-2 challenge to neutralize SARS-CoV-2 was assessed *in vitro* as previously described (107). After inactivation of complement for 30 min at 56 C°, sera were prepared in duplicates as two fold serial dilutions in Minimum Essential Medium (MEM) supplemented with 10% FBS and penicillin/streptomycin in 96-well cell culture plates (Sarstedt). To each serum dilution and the respective control wells, 40 pfu of SARS-CoV-2 was added and neutralization was allowed to proceed for 30 min at room temperature. Afterwards, approximately 1 × 10^4^ Vero E6 cells were added to each well. Subsequently, the plates were incubated at 37 C° under a 5% CO_2_ atmosphere for 3 days, fixed with 4% formaldehyde and stained with 0.75% crystal violet (aqueous solution) for quantification of cytopathic effects (CPE). Virus neutralization was considered successful in wells with no evidence of CPE and the last effective serum dilution was counted.

### 2.13 Statistical analysis

FACS results were evaluated with FlowJo V10.8.0 (Tree Star, Inc). Statistical analyses were performed using GraphPad Prism 9.5.0. The statistical details of all experiments are described in the respective figure legends. Significance of the data was assumed if p ≤ 0.05.

## 3 Results

### 3.1 Design of the pan-coronavirus vaccine

**Figure 1:**
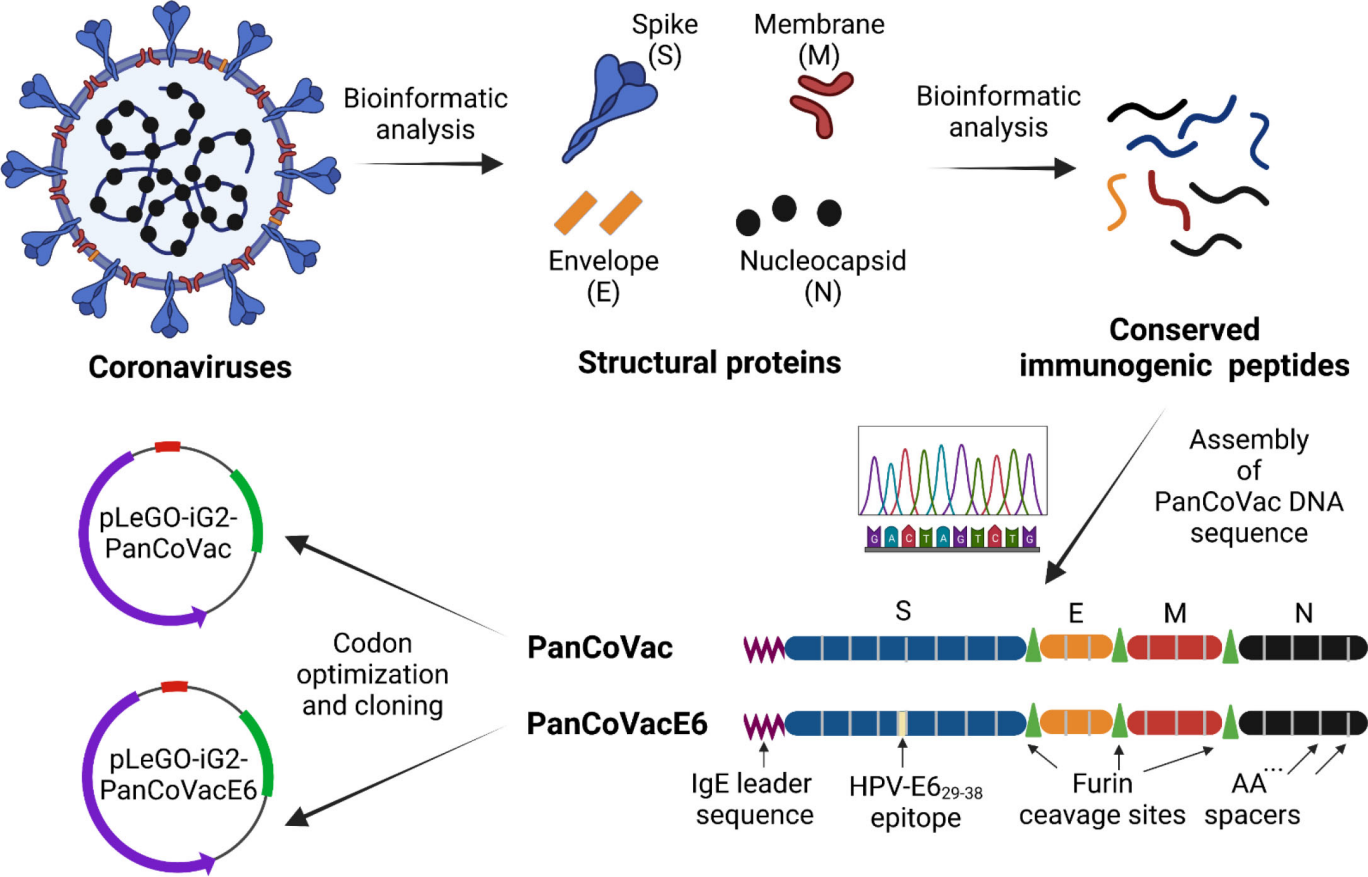
PanCoVac design. Structural proteins (S, E, M, N) from coronaviruses were analyzed. Conserved immunogenic peptides from all structural proteins were identified by bioinformatics analyses. The conserved immunogenic peptides from each protein were linked together with double alanine (AA) spacers resulting in four polypeptide blocks which were fused together by furin cleavage sites. An IgE leader sequence was attached to the N terminus. In order to investigate processing and presentation, the HPV 16 E6_29−38_ (TIHDIILECV) epitope, which binds to HLA-A^*^ 0201, was inserted in the middle of the S module (PanCoVacE6). The amino acid sequences of PanCoVac and PanCovacE6 were codon optimized for human expression and cloned into pLeGo-iG2 plasmids (pLeGo-iG2-PanCoVac, pLeGo-iG2-PanCoVacE6).

Using bioinformatic tools, we deconstructed coronavirus genomes and designed a DNA sequence (PanCoVac) encoding the conserved T-cell epitopes of all structural proteins (S, E, M, and N) from coronaviruses (**Figure 1**). The most essential step during processing and presentation of T cell epitopes is binding to MHC molecules, which are extremely polymorphic. CD8+ T cells recognize peptides bound to the polymorphic regions of MHC class I (MHC-I) molecules on the surface of virus-infected cells. These peptides are mostly derived from intracellular proteins undergoing proteasomal degradation in the cytosol. The resulting cytosolic peptides are transferred *via* transporter associated with antigen processing (TAP) molecules into the endoplasmic reticulum (ER) and subsequently loaded onto MHC-I molecules (108). In contrast, CD4+ T cells detect peptides bound to MHC class II (MHC-II) molecules on the surface of professional APCs. These peptides are obtained from extracellular proteins after cellular uptake and digestion (108).

Conserved regions of at least 8 amino acids from the structural proteins of SARS-CoV-1 (Tor2), ancestral SARS-CoV-2 (Wuhan-Hu-1), SARS-CoV-2 variants (B.1.1.7, Alpha; B.1.351, Beta; B.1.617.2, Delta; P.1, Gamma; BA.1, Omicron), and common cold coronaviruses (HKU1, 229E, NL63 and OC43) were considered. The alignment of these conserved amino acid sequences with PanCoVac is shown in **Supplementary Figure 1**. PanCoVac-encoded peptides are supposed to bind to all nine HLA-I supertypes, which are defined as groups of molecules that share largely overlapping peptide binding specificities (109), as well as other common HLA-I alleles (**Supplementary Table 2**). In addition, we determined PanCoVAc-encoded peptides that are supposed to bind to HLA-II molecules (**Supplementary Table 3**). Due to the high and comprehensive workload, we have not analyzed all HLA-II molecules but focused on frequent HLA-DR alleles (HLA-DRB1*01:01, HLA-DRB1*04:01, HLA-DRB1*07:01, HLA-DRB1*09:01, HLA-DRB1*1302, HLA-DRB1*15:01, HLA-DRB5*01:01). A very high percentage of experimentally validated HLA-I epitopes matches those that have been predicted by *in silico* analysis but experimental epitope screenings may be slightly biased due to the low frequency of some alleles analyzed (110). Thus, most of the SARS-CoV-2-derived peptides that have been shown to stimulate T cells from convalescent individuals are present in PanCoVac (42, 111). PanCoVac also encodes immunodominant CD8+ T cell epitopes (e.g. N105–113, SPRWYFYYL) and CD4+ T cell epitopes (e.g. M176–190, LSYYKLGASQRVAGD), which have broad HLA binding capacity as their main feature (42, 111).

The DNA sequences encoding the identified T cell epitopes were fused to generate a “string of beads” multi-epitope vaccine. It has been demonstrated that oligo-alanine spacing of epitopes can increase their processing and recognition by T cells (112, 113). For this reason, we joined epitopes belonging to the same structural protein by double alanine linkers (AA) thereby generating a single immunogenic compact module. The different immunogenic modules (S, E, M, and N) were separated by furin cleavage sites (**Figure 1**). Furin is a cellular endoprotease that is principally located in the *trans*-Golgi network (TGN), which is responsible for sorting secretory pathway proteins to their final destinations, including the cell surface, endosomes, lysosomes and secretory granules (114). Importantly, furin processes T cell epitopes independently from TAP and the proteasome (115–117). In addition, an immunoglobin E (IgE) leader sequence consisting of 18 amino acids (118) was attached to the 5´end (N-Terminus) of PanCoVac to achieve strong expression (**Figure 1**). Moreover, the PanCoVac was codon optimized to further increase its expression. Finally, *in-silico* antigenicity prediction using VaxiJen, showed an antigen score of 0.5308 indicating the probable antigenic nature of PanCoVac. Neither the *in silico* testing of the allergenic potential of PanCoVac protein using AllerCatPro 2.0 nor the peptide toxicity testing using ToxinPred provided evidence that PanCoVac is allergenic or yields additional toxic peptides as compared to the original sequences of the viral proteins. Accordingly, bioinformatic tools predicted that PanCoVac is probably an antigenic protein but has no allergic or toxic side effects. PanCoVac can be loaded onto any available vaccine platform to create coronavirus vaccines that could provide broad, robust, and durable T cell responses.

### 3.2 Processing and presentation of PanCoVac-encoded epitopes *in vitro*

**Figure 2:**
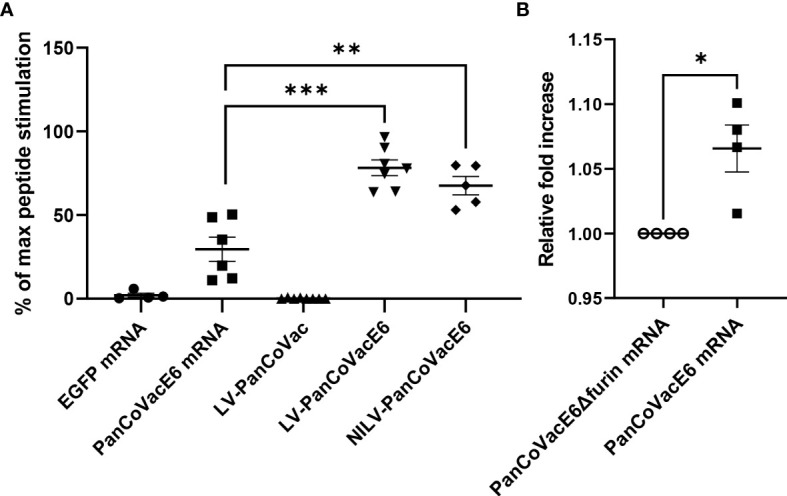
Stimulation of HPV E6-specific reporter T cells by PanCoVacE6 expressing cells. **(A)** U251 cells were transduced with PanCoVac or PanCoVacE6 using LV or NILV. In addition, U251 cells were transfected with either EGFP mRNA or PanCoVacE6 mRNA. After 18 h, the medium was removed and HPV E6 peptide-specific reporter cells were added at a ratio 2:1 for 24 h. Subsequently, the cells were collected, washed and stained with BV711 mouse anti-human CD3 antibody and live/dead Zombie Violet dye. Stimulation of reporter cells is given as percentage of maximal peptide stimulation, i.e. stimulation of reporter cells with U251 cells pulsed with HPV E6 peptide (TIHDIILECV). Results are derived from at least 5 independent experiments; error bars represent the mean ± SEM.; ***P < 0.001; **P < 0.01; ns not significant, unpaired t-test. **(B)** U251 cells were transfected with PanCoVacE6Δfurin mRNA (control) and PanCoVacE6 mRNA, respectively. HPV E6 peptide-specific reporter cells were added to transfected U251 cells as described in **(A)**. The results are shown as fold change relative to the control. Results are derived from 4 independent experiments; error bars represent the mean ± SEM.; *P < 0.05, unpaired t-test.

We confirmed that PanCoVac is expressed, processed and presented *in vitro* by using a T cell reporter assay. For this purpose, we tagged PanCoVac in the middle of the S module with a sequence encoding the HLA-A^*^ 0201-binding epitope E6_29−38_ (TIHDIILECV) derived from the E6 protein of HPV type 16 resulting in PanCoVacE6 (**Figure 1**) (102). We transduced U251 cells (HLA-A^*^ 0201+) with LV or NILV expressing PanCoVacE6 or untagged PanCoVac as a negative control. U251 cells were also transfected with PanCoVacE6 mRNA or EGFP mRNA as a negative control. U251 cells transfected with PanCoVacE6 mRNA, but not U251 cells transfected with EGFP mRNA, stimulated HPV E6-specific reporter T cells (**Figure 2A**). Moreover, U251 cells transduced with LV or NILV expressing PanCoVacE6 both strongly activated HPV E6_29−38_ specific reporter T cells whereas U251 cells transduced with untagged PanCoVac did not (**Figure 2A**). We also transfected U251 cells with PanCoVac mRNA containing a FLAG tag-encoding sequence and detected PanCoVac in western blot analysis using antibodies against the FLAG tag (**Supplementary Figure 2**).

Finally, we tested whether the furin cleavage sites of PanCoVac affects processing and presentation the HPV E6_29−38_ –peptide. For this purpose, we compared PanCoVacE6 with a PanCoVacE6 construct that has no furin cleavage sites (PanCoVacE6Δfurin). We observed a very small but significant increase in HPV E6-specific reporter T cell activation when U251 cells transfected with PanCoVacE6 (containing furin cleavage sites) were used for stimulation as compared to cells transfected with PanCoVacE6Δfurin (**Figure 2B**). However, the binding affinity of the HPV E6_29−38_ –peptide for HLA-A^*^ 0201 is very high (102) and our reporter T cell assay operates in the saturated range. Thus, the positive effect of furin cleavage on processing and presentation of PanCoVac-encoded epitopes with lower binding affinity for MHC-I molecules is most likely much more pronounced. Altogether, these results strongly suggest PanCoVac-encoded epitopes are processed and presented in cells expressing PanCoVac.

### 3.3 Mild course of SARS-CoV-2 infection in Roborovski dwarf hamsters after vaccination with NILV-PanCoVac

**Figure 3:**
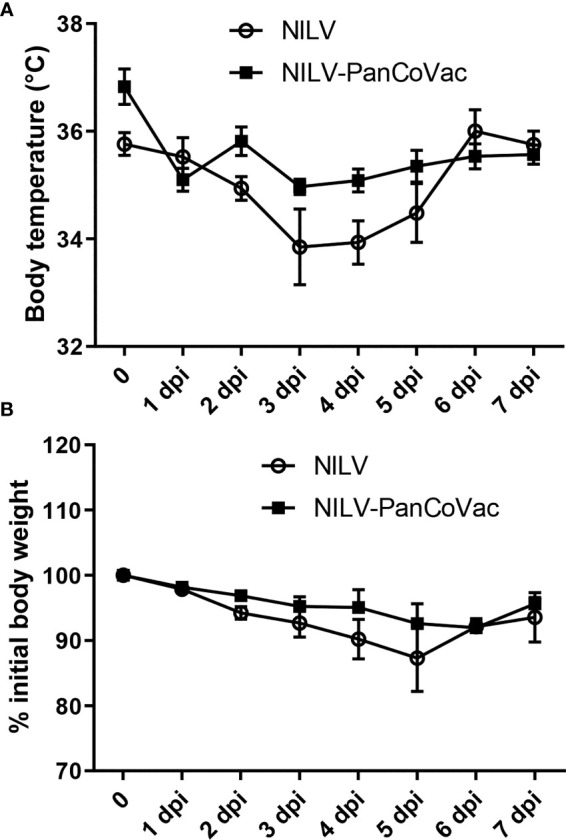
Body weight, body temperature and lung histopathology after challenge of vaccinated dwarf hamsters with SARS-CoV-2. Body temperature **(A)** and body weight **(B)** of dwarf hamsters vaccinated with NILV-PanCoVac or NILV were monitored on a daily basis until the experiment was terminated at 7 days post infection (dpi).

The protective effect of PanCoVac was tested in Roborovski dwarf hamsters, which represent an appropriate model for analyzing the pathology of COVID-19 (104, 119). To this end, hamsters were vaccinated i.n. either with a single-low dose (1 × 10^5^ pfu) of NILV-PanCoVac (9 animals) or empty NILV particles (9 animals) as a control. After 21 days, the animals were challenged i.n. with a sublethal dose (1×10^4^ pfu) of the ancestral SARS-CoV-2 (Wuhan) strain. We observed a drop in body temperature in the control group vaccinated with empty NILV particles (**Figure 3A**), which indicates a more severe course of SARS-CoV-2 infection in Roborovski dwarf hamsters (104). *Vis-a-vis*, NILV-PanCoVac vaccinated hamsters kept more steady body temperatures demonstrating a very mild infection course (**Figure 3A**). In addition, body weights of dwarf hamsters that had received empty NILV particles went down until 5 dpi then returned to pre-infection values whereas body weights of animals vaccinated with NILV-PanCoVac were stable (**Figure 3B**).

**Figure 4:**
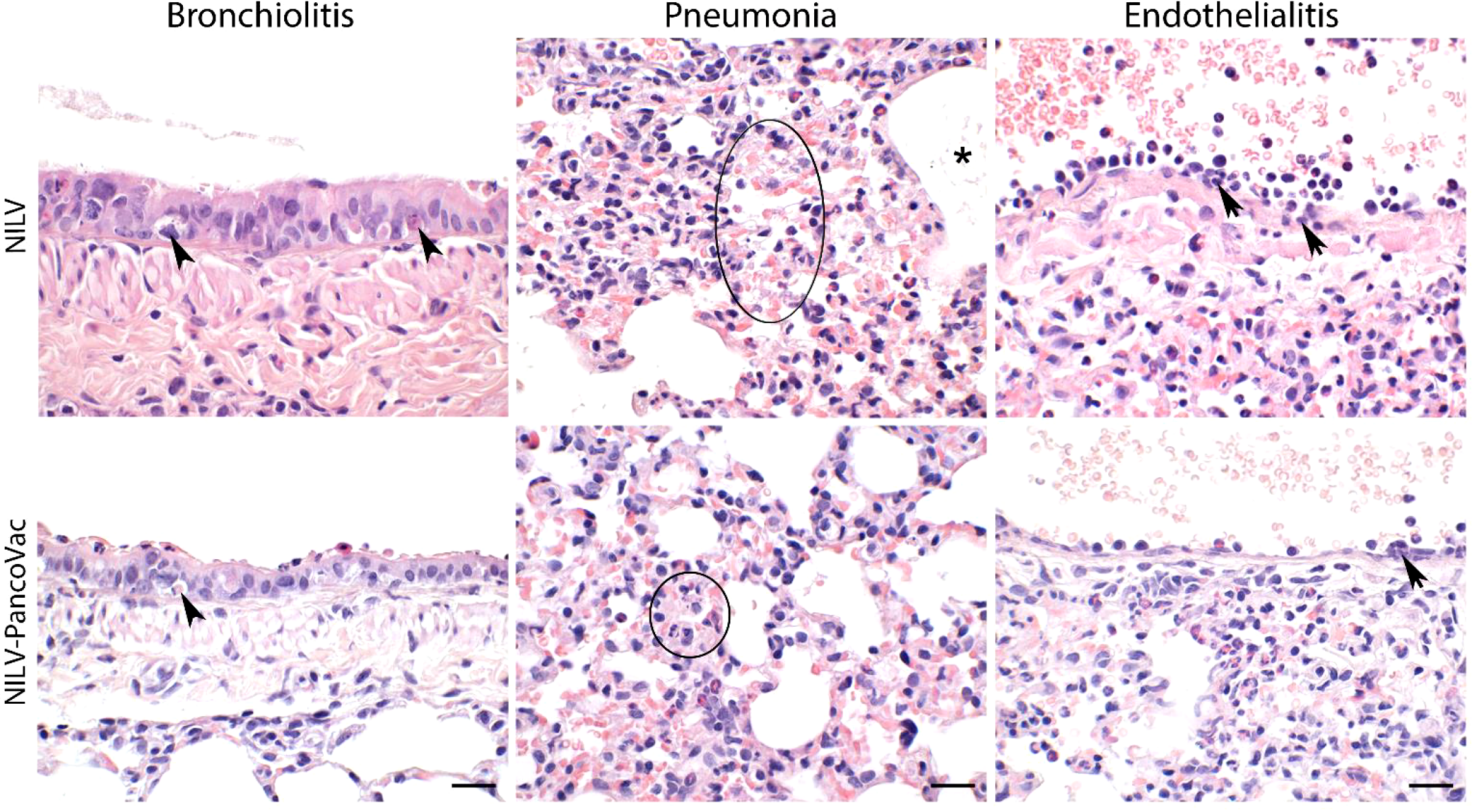
Representative histopathology of lung tissue derived from NILV or NILV-PanCoVac vaccinated Roborovski dwarf hamsters after challenge with SARS-CoV-2. At 2 dpi, bronchioli with mild bronchiolitis and moderate epithelial cell necrosis (arrow heads; left panel) were observed. The respiratory parenchyma presented with moderate to severe inflammation, alveolar wall necrosis (circle; central panel) and alveolar edema (asterisk; central panel). Histopathological analysis of blood vessels revealed endothelialitis (arrows; right panel). Haematoxylin and eosin stain; bars represent 20 µm.

**Figure 5:**
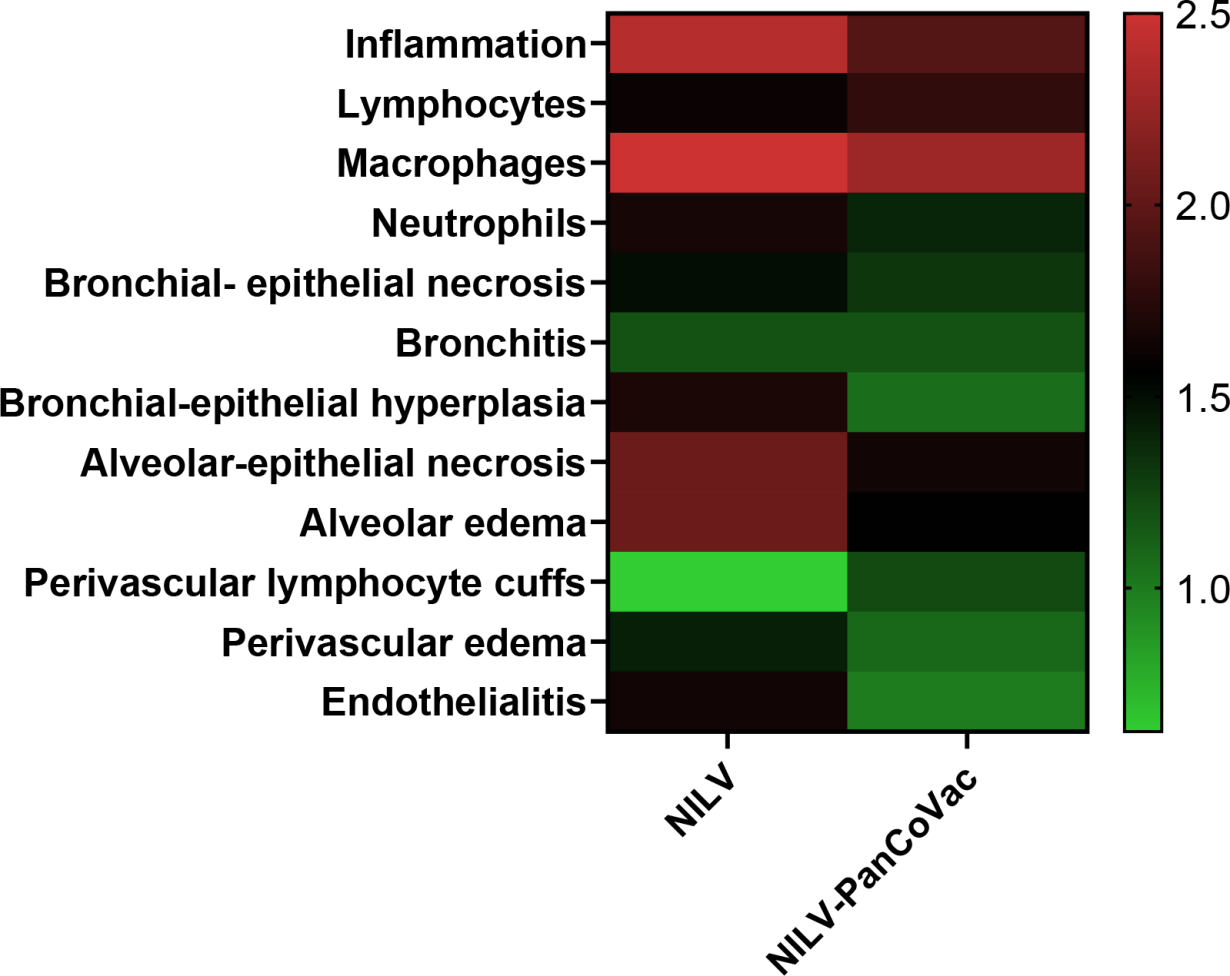
Histopathological scores of lung tissue derived from NILV or NILV-PanCoVac vaccinated Roborovski dwarf hamsters after challenge with SARS-CoV-2. At 2 days post infection (dpi), 5 dpi and 7 dpi, three animals of each group were sacrificed and histopathological changes in the lung were scored using a four-scale severity grading system (0: no lesions, 1: mild, 2: moderate, and 3: severe). The cumulative results are also shown.

At 2 dpi, 5 dpi, and 7 dpi, three animals of each group were sacrificed and sera, oropharyngeal swabs and lung tissue were collected for further analysis. The histopathological analysis of lung tissue also demonstrates the protective effect of a single-low dose of i.n. NILV-PanCoVac. The representative histopathology in **Figure 4** shows pathological changes at 2 dpi especially in lung tissue derived from NILV-vaccinated animals: bronchioli with mild bronchiolitis and moderate epithelial cell necrosis; respiratory parenchyma with moderate to severe inflammation, alveolar wall necrosis and alveolar edema; and blood vessels with endothelialitis. Cumulative histopathological scoring illustrates the finding that lung tissue from NILV-PanCoVac vaccinated animals was less affected by virus-induced damage and inflammation than the corresponding control tissue from animals vaccinated with empty NILV particles (**Figure 5**). The corresponding histopathological scoring at 2 dpi, 5 dpi and 7 dpi 2 is shown in **Supplementary Figure 3**. Altogether, these clinical and histopathological findings demonstrate a comparatively mild course of SARS-CoV-2 infection in Roborovski dwarf hamsters vaccinated i.n. with NILV-PanCoVac.

### 3.4 Independence of NILV-PanCoVac induced protection from SARS-CoV-2 neutralizing antibodies

**Figure 6:**
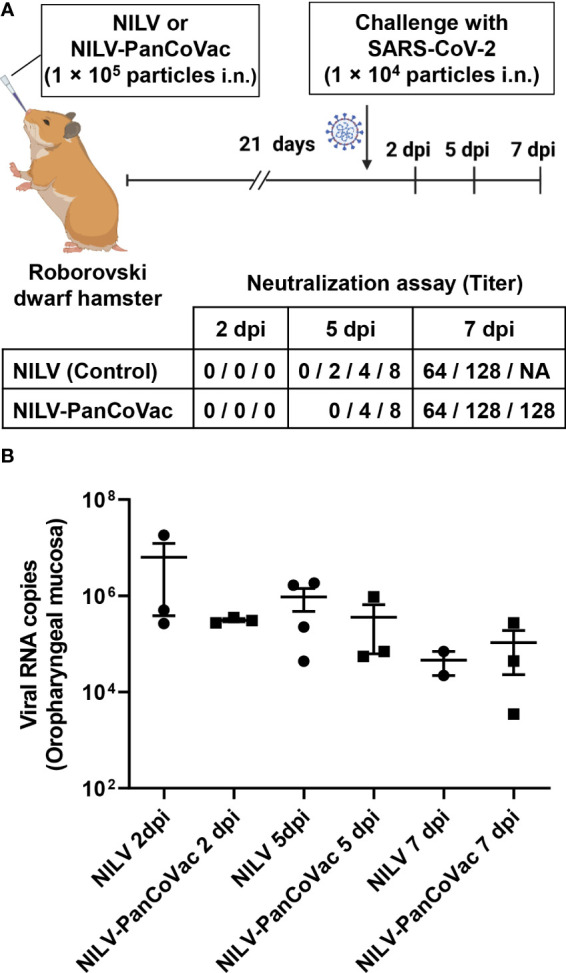
Titers of SARS-CoV-2 neutralizing antibodies in sera and SARS-CoV-2 quantification in oropharyngeal swabs derived from of infected dwarf hamsters. Roborovski dwarf hamsters (*P. roborovskii*) were immunized i.n. with 1×10^5^ particles either NILV-PanCoVac (9 animals) or NILV (9 animals). After 21 days, the hamsters were challenged with a sublethal dose (1×10^4^ pfu) of the ancestral SARS-CoV-2 (Wuhan) strain. At 2 dpi, 5 dpi, and 7 dpi, three animals of each group were sacrificed and sera and oropharyngeal swabs were collected for further analysis. **(A)** Titers of SARS-CoV-2 neutralizing antibodies were determined. Shown is the maximal serum dilution that still completely neutralized SARS-CoV-2 in a cell culture assay. **(B)** SARS-CoV-2 genome copy numbers in oropharyngeal swabs derived from animals vaccinated either with NILV-PanCoVac or NILV (control) were determined. One animal in the NILV-vaccinated group scheduled for the analysis at 7 dpi (Hamster No. 8) died at 4 dpi. The corresponding serum and oropharyngeal swab, respectively was analyzed together with the probes scheduled for 5 dpi resulting in 4 measured values at this time point.

Although PanCoVac is a T cell-based vaccine, we could not exclude *a priori* that NILV-PanCoVac induces virus-specific humoral immune responses. To clarify this issue, we performed neutralization assays with sera from Roborovski dwarf hamsters after i.n. vaccination with NILV-PanCoVac and empty NILV particles, respectively. The systemic humoral immunity induced by i.n. vaccination is usually comparable to or even stronger than that after intramuscular injection (25, 120, 121). This is also true for hamsters immunized against SARS-CoV-2 with a single-low dose of a vectored S protein-based vaccine administered i.n (120). In serum neutralization assays, we did not detect any difference in the timing or level of neutralizing antibody production between animals vaccinated i.n. with NILV-PanCoVac and those vaccinated i.n. with empty NILV particles (**Figure 6A**). Neutralizing antibody production was not detectable at 2 dpi and started at 5 dpi (**Figure 6A**). At 7 dpi, high neutralizing antibody titers were measured in both NILV-PanCoVac animals and animals immunized with empty NILV (**Figure 6A**). This result suggested that the protective effect of NILV-PanCoVac was not associated with sterilizing immunity, which requires induction of neutralizing antibodies at the site of infection (122). To analyze sterilizing immunity, we determined the viral load in the oropharyngeal mucosa using quantitative RT-qPCR. As shown in **Figure 6B**, we did not observe significant differences between NILV-PanCoVac vaccinated and control animals regarding the viral load in the oropharynx. This experiment confirms that NILV-PanCoVac did not stimulate production of neutralizing antibodies in the oropharyngeal mucosa. Thus, in Roborovski dwarf hamsters a single-low dose of NILV-PanCoVac did induce SARS-CoV-2 neutralizing antibodies neither systemically nor in the mucosa of the oropharynx.

### 3.5 Early protective effect of NILV-PanCoVac in Roborovski dwarf hamsters

**Figure 7:**
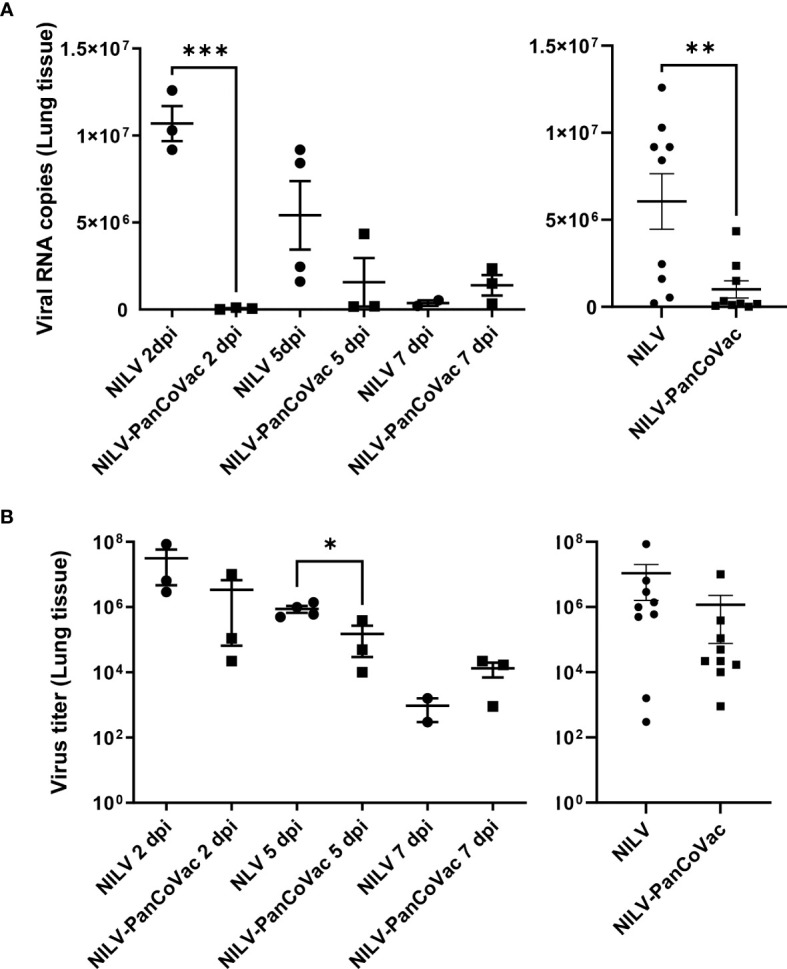
SARS-CoV-2 quantification in lung tissue from Roborovski dwarf hamsters after challenge with SARS-CoV-2. Roborovski dwarf hamsters (*P. roborovskii*) were immunized i.n. (1×10^5^ particles) with NILV-PanCoVac (9 animals) or NILV (9 animals). After 21 days, the hamsters were challenged with a sublethal dose (1×10^4^ pfu) of the ancestral SARS-CoV-2 (Wuhan) strain. At 2 dpi, 5 dpi and 7 dpi, three animals of each group were sacrificed and lung tissue was analyzed. **(A)** SARS-CoV-2 genome copy numbers per 2.5 mg lung tissue derived from animals vaccinated either with NILV-PanCoVac or NILV (control) were determined at the indicated time points (left graph). The cumulative results are also shown (right graph). **(B)** Virus titers in 50 mg lung tissues from animals vaccinated either with NILV-PanCoVac or NILV (control) were analyzed at the indicated time points (left graph). The cumulative results are also shown (right graph). Error bars represent the mean ± SEM; ***P < 0.001; **P < 0.01; *P < 0.05, unpaired t-test. One animal in the control group (Hamster No. 8) scheduled for the analysis at day 7 dpi died at 4 dpi. The corresponding lung tissue was analyzed together with the probes scheduled for 5 dpi resulting in 4 measured values at this time point.

Next, we determined the viral load in lung tissue. The cumulative SARS-CoV-2 genome copy numbers (**Figure 7A**, right graph) and virus titers (**Figure 7B**, right graph) were significantly lower in lung tissue from NILV-PanCoVac vaccinated animals as compared to control animals that had received empty NILV particles. Intriguingly, the protective effect of NILV-PanCoVac vaccination on the viral load in lung tissue was already very strong at 2 dpi (**Figures 7A, B**, left graphs). At this time point, SARS-CoV-2 neutralizing antibodies were detectable neither in animals vaccinated with NILV-PanCoVac nor in animals vaccinated with empty NILV particles (**Figure 6A**). Taken together these results provide evidence that the protective effect of i.n. administered NILV-PanCoVac in Roborovski dwarf hamsters challenged with SARS-CoV-2 was most likely due to airway memory T cells that can mount a strong early antiviral response.

## 4 Discussion

We designed a codon-optimized universal coronavirus vaccine (PanCoVac) encoding conserved T-cell epitopes derived from all structural proteins (S, E, M, and N) for use in human populations. Using a tagged version of PanCoVac, we demonstrated in T cell reporter assays that the PanCoVac-design allows expression, processing, and presentation by human cells. Finally, we evaluated the vaccine in the Roborovski dwarf hamster model of COVID-19. We observed a milder course of sublethal SARS-CoV-2 infection in Roborovski dwarf hamsters after vaccination with a single-low dose of NILV-PanCoVac as compared to animals receiving empty NILV particles. In vaccinated animals the viral load was reduced within 2 days after challenge. The NILV-PanCoVac induced immunity, however, was not sterilizing and independent of neutralizing antibodies.

Conserved T-cell epitopes of coronaviruses can elicit broadly cross-reactive T cells. For example, CD8+ T cells specific for a highly conserved N-encoded epitope of SARS-CoV-2 were found in unexposed donors and could be stimulated by homologous peptide sequences of seasonal coronaviruses (123). Mounting evidence suggests that during vaccination or infection, cross-reactive T cells are integrated into SARS-CoV-2 specific immunity and contribute to protection from COVID-19 (41, 124, 125). Epidemiological studies revealed that individuals infected recently with common cold coronaviruses had less severe COVID-19 outcomes (126, 127). Healthcare workers with cross-reactive T cells against the virus-encoded RNA polymerase, a protein that is highly conserved across coronaviruses, cleared subclinical SARS-CoV-2 infection before seroconversion (88). This is confirmed by studies of household contacts of COVID-19 patients demonstrating that induction of virus-specific T cell responses without seroconversion protect from SARS-CoV-2 infection (81, 128). Finally, in the absence of neutralizing antibodies, T cells provided effective protection against the Beta variant of SARS-CoV-2 in a transgenic mouse model of SARS-CoV-2 infection (129). Thus, clinical studies and animal experiments suggest that cross-reactive T cells can clear SARS-CoV-2 independently of humoral immunity.

Why did pre-existing cross-reactive T cell immunity not have a greater impact on the course of the pandemic although it was detected in a large proportion of healthy, SARS-CoV-2–naive individuals? There are at least two mutually not exclusive explanations. Firstly, cross-reactive T cells protect from severe disease but less efficiently from infection and virus transmission to other persons. Secondly, cross-reactive immunity is virtually absent in individuals that are at risk of severe SARS-CoV-2 infection. Indeed, functional pre-existing SARS-CoV-2-reactive memory T cells are induced by common cold coronavirus in early childhood, peak at age six, and subsequently decline with age (130, 131). This finding explains the age-dependent ability to control SARS-CoV-2 infection with older adult people, who often suffer from comorbidities, at risk of an unfavorable outcome (132). In accordance, CD8+ T cells specific for conserved coronavirus epitopes are much more abundant in patients with mild COVID-19 as compared to individuals with more severe illness (133). This indicates that especially individuals at high-risk of COVID-19 could benefit from vaccines that strengthen T cell responses against conserved coronavirus epitopes.

Current COVID-19 vaccines are approved for intramuscular application notwithstanding that SARS-CoV-2 is spreading *via* the mucosa of the respiratory tract. For this type of viral pathogens, the innate and adaptive immune responses in the lung and airways following infection and vaccination play a pivotal role (reviewed in (25, 134). In SARS-CoV-2 susceptible mice, even a single-dose i.n. immunization with a replication-deficient adenoviral vector expressing the RBD of SARS-CoV-2 S protein induced robust immunity both in the mucosa of the respiratory tract and systemically (135, 136). Along this line, a trivalent vaccine based on adenoviral vectors expressing antigens derived from the S-protein, N protein, and RNA-dependent RNA polymerase induced local and systemic antibody responses and protected against both the ancestral SARS-CoV-2 strain and two VOCs (137).

After i.n. immunization with a single-low dose of NILV-PanCoVac, we observed a strong protective effect at 2 dpi. At this early time point, we did not detect neutralizing antibodies in the sera of vaccinated animals suggesting that T cells were responsible. In accordance, antiviral CD8+ T cells induced by a neutralizing antibody-independent i.n. vaccine curbed viral spread in the respiratory tract of macaques after SARS-CoV-2 challenge (138). PanCoVac also encodes a highly conserved region of the N protein, which not only cross-protected mice from human and bat coronaviruses after i.n. vaccination but also is presented by human MHC-II molecules (139). The protective effect was observed within 1-2 days after challenge and mediated by memory CD4+ T cells that secreted interferon-γ and supported strong innate as well as virus-specific CD8+ T cell responses (139). Moreover, systemic immunization of mice with dendritic cells (DCs) presenting a single SARS-CoV-1 epitope followed by i.n. boosting with recombinant vaccinia virus encoding the same epitope resulted in accumulation of virus-specific memory CD8+ T cells in lungs and protected from lethal infection (140). Similarly, repeated booster vaccinations with a single T cell epitope induced CD8+ T cells that protected against lethal SARS-CoV-2 infection in a mouse model of COVID-19 (141). Others investigators have also demonstrated that in the absence of neutralizing antibodies, lung-resident memory CD4+ and CD8+ T cells provide effective protection against SARS-CoV-2 (142). It is conceivable that PanCoVac could induce similar memory T cells in Roborovski dwarf hamsters. Although virus neutralization is a key function of antiviral antibodies, they can also contribute to protection by other means, e.g. *via* binding to and triggering Fc receptors (143). Thus, we cannot categorically exclude the possibility that PanCoVac-induced antibodies contribute to the immune response against SARS-CoV-2. Altogether, PanCoVac-encoded conserved T cell epitopes could generate cross-reactive T cells in vaccinated humans that act as a first layer of defense. In accordance, T cells in the respiratory tract of a large proportion of unexposed individuals cross-react with SARS-CoV-2 and may enable rapid antiviral immune responses (86, 144, 145).

Vaccination with a single-low dose of NILV-PanCoVac did not prevent infection of the oropharynx, the site of SARS-CoV-2 entry. However, sterilizing immunity with prevention of virus transmission is difficult to achieve by single vaccination i.n. and requires vaccine boosts (122). Indeed, the protective effect of i.n. immunization can be enhanced if combined with systemic priming (prime-boost regime). For example, systemic priming and i.n. boost with NILV expressing S protein in the Syrian hamster model resulted in strong vaccine efficacy and only limited lung damage after challenge with SARS-CoV-2 (97). Similarly, prime and i.n. boost with adenoviral vector expressing both the S protein and N protein resulted in complete protection against SARS-CoV-2 (146). Moreover, Syrian hamsters immunized *via* the i.n. route with the S protein linked to outer membrane vesicles from *Neisseria meningitides* were protected from weight loss and viral replication in the lungs (147). In a mouse model of COVID-19, boosting mice i.n. with non-replicating adenovirus vectoring S protein after priming with lipid nanoparticles (LNPs) containing S protein-mRNA (heterologous prime-boost regime) improved SARS-CoV-2 immunity in the lung (148). Finally, a heterologous prime-boost regime using an i.n. unadjuvanted S protein boost after intramuscular priming with LNPs containing S protein-mRNA induces neutralizing immunoglobulin A at the respiratory mucosa and simultaneously increases systemic immunity, which protects from lethal SARS-CoV-2 infection (149). Intriguingly, i.n. boosting with a divergent S protein from SARS-CoV-2 induces mucosal immunity against diverse sarbecovirus clades (149).

The NILV vaccine platform has the advantage that vaccines get access to non-proliferating cells including DCs, which are located in the mucosa of the respiratory tract. NILV-transduced DCs show strong and reliable expression of the vectored protein (150–153). Importantly, DCs play a pivotal role in successful vaccinations (154). Firstly, they transport vaccine-encoded antigen to the T cell areas of lymphoid organs. Secondly, they efficiently process and present this antigen as peptides bound to MHC molecules to activate antigen-specific T cells. There are possible advantages of using NILV as a vaccine platform as compared to adenoviral vectors. For example, immunization with lentiviral vectors generates highly multifunctional CD8+ T cells and primes development of CD8+ T cells with central memory phenotype (155). In contrast to adenoviral vector, the problem of pre-existing immunity to the vector, which can prevent successful vaccination, does not exist for NILV due to pseudotyping with VSV-G. Thus, after i.n. immunization NILV-PanCoVac could induce a long-lasting, multi-functional T cell immunity against SARS-CoV-2.

Our study has several limitations. First of all, PanCoVac was designed for binding to a broad range of common human MHC molecules, which are highly polymorphic. Although not characterized, it is likely that the MHC molecules of dwarf hamster are less diverse and have different peptide binding traits when compared to human molecules. This suggests that only a single or very few PanCoVac-encoded epitopes bind to MHC molecules of Roborovski dwarf hamsters. Secondly, PanCoVac was codon optimized for expression in human cells. Thus, it is unlikely that PanCoVac-based vaccines develop their full protective potential in the Roborovski dwarf hamster model of COVID-19. Thirdly, we did not prime *via* systemic injection of a PanCoVac-based vaccine, which provides broader mucosal protection against SARS-CoV-2 after i.n. boosting (97, 149). Fourthly, we used a single-low dose of NILV particles (1 × 10^5)^ i.n. whereas a recent study, which analyzed the protective effect of NILV expressing S protein in the Syrian hamster model of COVID-19, primed systemically and boosted with a high dose of NILV particles (1 × 10^8^) i.n (97). Moreover, we challenged vaccinated dwarf hamster only with the ancestral strain of SARS-CoV- 2 but not against currently circulating VOCs. However, T cell epitopes are very resistant to antigenic evolution of SARS-CoV-2 as compared to epitopes recognized by neutralizing antibodies (60, 68, 69, 156). Thus, PanCoVac is most likely also protective against SARS-CoV-2 variants and subvariants. Finally, we did not study T cell responses because the necessary tools and reagents for studying specific T cell responses in Roborovski dwarf hamsters are not available.

In summary, we generated a universal vaccine (PanCoVac) encoding cross-reactive T cell epitopes that are highly conserved across structural proteins of human coronaviruses and bind to common human MHC molecules. Despite of the huge differences between human and hamster MHC molecules a single-low dose of a PanCoVac-based vaccine i.n. induced an early protective effect in Roborovski dwarf hamsters independently of neutralizing antibodies. The use of (HLA-) humanized animal models will allow for further efficacy studies of PanCoVac-based vaccines *in vivo*. In humans, PanCoVac could induce broad T cell responses that prevent severe disease courses leading to hospitalizations and death.

## Data availability statement

The original contributions presented in the study are included in the article/**Supplementary Material**. Further inquiries can be directed to the corresponding authors.

## Ethics statement

The animal experiments were reviewed and approved by the Landesamt für Gesundheit und Soziales (LAGeSo) in Berlin, Germany (permit number 0086/20). They were performed in compliance with relevant national and international guidelines for responsible care and humane use of animals in the BSL-3 facility at the Institute of Virology, Free University Berlin, Germany.

## Author contributions

MA and MR designed experiments, performed experiments, analyzed results, and co-wrote the article. JW, OB, RE, JK, DV, JA, TF, AV, AG, LH, IFM, FM-M, GW and NE conducted experiments and data analysis. JT and GS directed the project, designed experiments, interpreted results, and wrote the article. All authors contributed to the article and approved the submitted version.

## Supplementary material

Supplementary Figure 1 (Alignment of PanCoVac with SARS-CoV-2 variants).

Supplementary Figure 2 (Western blot analysis of FLAG tagged PanCoVac).

Supplementary Figure 3 (Histopathological scoring of tissue).

Supplementary Table 1 (Sex distribution of Roborovski dwarf hamsters).

Supplementary Table 2 (HLA-I alleles presenting PanCoVac-encoded peptides).

Supplementary Table 3 (HLA-II alleles presenting PanCoVac-encoded peptides).
