# Supplementary material for: Early protective effect of a (“pan”) coronavirus vaccine (PanCoVac) in Roborovski dwarf hamsters after single-low dose intranasal administration
Source: Front Immunol. 2023 Jul 13;14:1166765. doi: 10.3389/fimmu.2023.1166765 (PMC10372429; doi:10.3389/fimmu.2023.1166765)
Supplement: Supplementary Figure 1 — (Alignment of PanCoVac with SARS-CoV-2 variants). [file Image_1.pdf]

```
sp|229E|           ------------------------------------------------------------
sp|NL63|           MKLFLILLVLPLAS----CFFTCNSNANLSMLQLGVPDNSSTIVTGLLPTHWFCANQSTS
sp|HKU1            ---MLLIIFILPTTLAVIGDFNCTNF---------AINDKNTTVPRIS---EY----VVD
sp|OC43|           --MFLILLISLPTAFAVIGDLNCTLDPSL----KGSFNNRDTGSPSIS---ID----TVD
sp|Tor2|           --MFIFLLFLTLTSG--SDLDRCTTFDDV----QAPN---------------Y----TQH
sp|BA.1.1|         --MFVFLVLLPLVSS--QCVNLKT--RTQ----LPPA---------------Y------T
sp|B.1.617.2|      --MFVFLVLLPLVSS--QCVNLRT--RTQ----LPPA---------------Y------T
sp|B.1.351|        --MFVFLVLLPLVSS--QCVNFTT--RTQ----LPPA---------------Y------T
sp|B.1.1.7|        --MFVFLVLLPLVSI--QCVNLTT--RTQ----LPPA---------------Y------T
sp|Wuhan-Hu-1|     --MFVFLVLLPLVSS--QCVNLTT--RTQ----LPPA---------------Y------T
sp|p.1|            --MFVFLVLLPLVSS--QCVNLTT--RTQ----LPPA---------------Y------T
sp|PanCoVac|       ------------------------------------------------------------

sp|229E|           ------------------------------------------------------------
sp|NL63|           VYSANGFFYIDVGNHR-----SAFALHTGYYDANQYYIYVTNEIGLNASVTLKICKFSRN
sp|HKU1            VSYGLGTYY-----ILDRVYLNTTILFTGYFPKSGANF---RDLSLKGTTYLSTLWYQKP
sp|OC43|           VTNGLGTYY-----VLDRVYLNTTLFLNGYYPTSGSTY---RNMALKGTDLLSTLWFKPP
sp|Tor2|           TSSMRGVYYPDEIFRSDTLYLTQDLFLPFYSNVTGFHT---IN-----------HTFGNP
sp|BA.1.1|         NSFTRGVYYPDKVFRSSVLHSTQDLFLPFFSNVTWFHV---I--S--G--TNGTKRFDNP
sp|B.1.617.2|      NSFTRGVYYPDKVFRSSVLHSTQDLFLPFFSNVTWFHA---IHVS--G--TNGTKRFDNP
sp|B.1.351|        NSFTRGVYYPDKVFRSSVLHSTQDLFLPFFSNVTWFHA---IHVS--G--TNGTKRFANP
sp|B.1.1.7|        NSFTRGVYYPDKVFRSSVLHSTQDLFLPFFSNVTWFHA---IHVS--G--TNGTKRFDNP
sp|Wuhan-Hu-1|     NSFTRGVYYPDKVFRSSVLHSTQDLFLPFFSNVTWFHA---IHVS--G--TNGTKRFDNP
sp|p.1|            NSFTRGVYYPDKVFRSSVLHSTQDLFLPFFSNVTWFHA---IHVS--G--TNGTKRFDNP
sp|PanCoVac|       ------------------------------------------------------------

sp|229E|           ---------------------------------------------------MFVLLVAY-
sp|NL63|           TTFDFLSNA----SSSFDCIVNLLFTEQLGAPLGITISGETVRLHLYNVTRTFYVPAAYK
sp|HKU1            FLSDFNNGIFSRVKNTKLYVNKTLYSEFSTIVIGSVFINNSYTIVVQPH-----------
sp|OC43|           FLSDFINGIFAKVKNTKVFKDGVMYSEFPAITIGSTFVNTSYSVVVQPR--------TIN
sp|Tor2|           V-IPFKDGIYFAATEKSNVV--------RGWVFGSTMNNKSQSVIIINN-----------
sp|BA.1.1|         V-LPFNDGVYFASIEKSNII--------RGWIFGTTLDSKTQSLLIVNN-----------
sp|B.1.617.2|      V-LPFNDGVYFASIEKSNII--------RGWIFGTTLDSKTQSLLIVNN-----------
sp|B.1.351|        V-LPFNDGVYFASTEKSNII--------RGWIFGTTLDSKTQSLLIVNN-----------
sp|B.1.1.7|        V-LPFNDGVYFASTEKSNII--------RGWIFGTTLDSKTQSLLIVNN-----------
sp|Wuhan-Hu-1|     V-LPFNDGVYFASTEKSNII--------RGWIFGTTLDSKTQSLLIVNN-----------
sp|p.1|            V-LPFNDGVYFASTEKSNII--------RGWIFGTTLNSKTQSLLIVNN-----------
sp|PanCoVac|       ------------------------------------------------------------

sp|229E|           ----------------A---LLHIAGCQTTNGTNTSHSVCNGCVGHSENVFAVESGGYIP
sp|NL63|           LTKLSVKCYFNYSCVFSVVNATVTVNVTTHNGRVVNYTVCDDCNGYTDNIFSVQQDGRIP
sp|HKU1            ------------------NGVLEI--------TACQYTMCEYP-----------------
sp|OC43|           STQDG---------VNKLQGLLEV--------SVCQYNMCEYP-----------------
sp|Tor2|           ------------------STNVVI--------RACNFELCDNP-----------------
sp|BA.1.1|         ------------------ATNVVI--------KVCEFQFCNDP-----------------
sp|B.1.617.2|      ------------------ATNVVI--------KVCEFQFCNDP-----------------
sp|B.1.351|        ------------------ATNVVI--------KVCEFQFCNDP-----------------
sp|B.1.1.7|        ------------------ATNVVI--------KVCEFQFCNDP-----------------
sp|Wuhan-Hu-1|     ------------------ATNVVI--------KVCEFQFCNDP-----------------
sp|p.1|            ------------------ATNVVI--------KVCEFQFCNDP-----------------
sp|PanCoVac|       ------------------------------------------------------------

sp|229E|           SNFAFNNWFLLTNTSSVVDGVVRSFQPLLLNCLWSVSGSQFT----------TGFVYFNG
sp|NL63|           NGFPFNNWFLLTNGSTLVDGVSRLYQPLRLTCLWPVPGLKSS----------TGFVYFNA
sp|HKU1            ----HTICKSK-GSSRNESWH---FDKSEPLCLF---KKNFTYNVSTD------FLYFHF
sp|OC43|           ----HTICHPNLGNHFKELWH---YDTGVVSCLY---KRNFTYDVNAT------YLYFHF
sp|Tor2|           ----FFAVSKPMG----TQTHTMIFDNA-FNCTFEYISDAFSLDVSEKSGNFKHLREFVF
sp|BA.1.1|         ----FLD---HKNNKSWMESEFRVYSSA-NNCTFEYVSQPFLMDLEGKQGNFKNLREFVF
sp|B.1.617.2|      ----FLXVYYHKNNKSWMES--GVYSSA-NNCTFEYVSQPFLMDLEGKQGNFKNLREFVF
sp|B.1.351|        ----FLGVYYHKNNKSWMESEFRVYSSA-NNCTFEYVSQPFLMDLEGKQGNFKNLREFVF
sp|B.1.1.7|        ----FLGVYYHKNNKSCMESEFRVYSSA-NNCTFEYVSQPFLMDLEGKQGNFKNLREFVF
sp|Wuhan-Hu-1|     ----FLGVYYHKNNKSWMESEFRVYSSA-NNCTFEYVSQPFLMDLEGKQGNFKNLREFVF
sp|p.1|            ----FLGVYYHKNNKSWMESEFRVYSSA-NNCTFEYVSQPFLMDLEGKQGNFKNLREFVF
sp|PanCoVac|       ------------------------------------------------------------
```

```
sp|229E|            TGR-GDCKGFYSNASSDVIRYNINFEE----NLRRGTILFKTSYGAVVFYCTNNTLVSGD
sp|NL63|            TGSDVNCNGYQHNSVVDVMRYNLNFSANSLDNLKSGVIVFKTLQYDVLFYCSNSSSGVLD
sp|HKU1             YQERGTFYAYYAD---------------------SGMP-TTF-----------------L
sp|OC43|            YQEGGTFYAYFTD---------------------TGFV-TKF-----------------L
sp|Tor2|            KNKDGFLYVYKGYQPIDVVR-----------DLPSGFNTLKP-----------------I
sp|BA.1.1|          KNIDGYFKIYSKHTPII-VX-----------XXPQGFSALEP-----------------L
sp|B.1.617.2|       KNIDGYFKIYSKHTPINLVR-----------DLPQGFLALEP-----------------L
sp|B.1.351|         KNIDGYFKIYSKHTPINLVR-----------GLPQGFSALEP-----------------L
sp|B.1.1.7|         KNIDGYFKIYSKHTPINLVR-----------DLPQGFSALEP-----------------L
sp|Wuhan-Hu-1|      KNIDGYFKIYSKHTPINLVR-----------DLPQGFSALEP-----------------L
sp|p.1|             KNIDGYFKIYSKHTPINLVR-----------DLPQGFSALEP-----------------L
sp|PanCoVac|        ------------------------------------------------------------


sp|229E|            AHIPSGTVLGNFYCFVNTTI------------GNETTSAFVGALPKTVREFVISRTGHFY
sp|NL63|            TTIPFGPSSQPYYCFINSTI------------NTTHVSTFVGILPPTVREIVVARTGQFY
sp|HKU1             FSLYLGTLLSHYYVLPLTCN------AISSNTDNETLQYWVTPLSKRQ------------
sp|OC43|            FNVYLGMALSHYYVMPLTCI------RR--AKDGFSPEYWVTPLTPRQ------------
sp|Tor2|            FKLPLGINITNFRAILTAFS------PAQDIWGTSAAAYFVGYLKPTT------------
sp|BA.1.1|          VDLPIGINITRFQTLLALHRSYLTPGDSSSGWTAGAAAYYVGYLQPRT------------
sp|B.1.617.2|       VDLPIGINITRFQTLLALHRSYLTPGDSSSGWTAGAAAYYVGYLQPRT------------
sp|B.1.351|         VDLPIGINITRFQT---LHRSYLTPGDSSSGWTAGAAAYYVGYLQPRT------------
sp|B.1.1.7|         VDLPIGINITRFQTLLALHRSYLTPGDSSSGWTAGAAAYYVGYLQPRT------------
sp|Wuhan-Hu-1|      VDLPIGINITRFQTLLALHRSYLTPGDSSSGWTAGAAAYYVGYLQPRT------------
sp|p.1|             VDLPIGINITRFQTLLALHRSYLTPGDSSSGWTAGAAAYYVGYLQPRT------------
sp|PanCoVac|        -----------------------------MDWT---------------------------


sp|229E|            INGYRYFSLGNVEAVNFNVTNAA-TTVCTVALASYADVLVNVSQTAIANIIYCN-SVINR
sp|NL63|            INGFKYFDLGFIEAVNFNVTTASATDFWTVAFATFVDVLVNVSATNIQNLLYCD-SPFEK
sp|HKU1             ------------------------------------YLLKFDNRGVITNAVDCSSSFFSE
sp|OC43|            ------------------------------------YLLAFNQDGIIFNAVDCMSDFMSE
sp|Tor2|            ------------------------------------FMLKYDENGTITDAVDCSQNPLAE
sp|BA.1.1|          ------------------------------------FLLKYNENGTITDAVDCALDPLSE
sp|B.1.617.2|       ------------------------------------FLLKYNENGTITDAVDCALDPLSE
sp|B.1.351|         ------------------------------------FLLKYNENGTITDAVDCALDPLSE
sp|B.1.1.7|         ------------------------------------FLLKYNENGTITDAVDCALDPLSE
sp|Wuhan-Hu-1|      ------------------------------------FLLKYNENGTITDAVDCALDPLSE
sp|p.1|             ------------------------------------FLLKYNENGTITDAVDCALDPLSE
sp|PanCoVac|        ------------------------------------WILFLV----------AAATRVHS


sp|229E|            LRCDQLSFDVPDGFYSTSPIQPVE------------------------------------
sp|NL63|            LQCEHLQFGLQDGFYSANFLDDNV------------------------------------
sp|HKU1             IQCKTKSLLPNTGVYDLSGFTVKPVATVHRRIPDLPDCDIDKWLNNFNVPSPLNWERKIF
sp|OC43|            IKCKTQSIAPPTGVYELNGYTVQPIADVYRRKPDLPNCNIEAWLNDKSVPSPLNWERKTF
sp|Tor2|            LKCSVKSFEIDKGIYQTSNFRVVPSGDVVRFPNITNLCPFGEVFNATKFPSVYAWERKKI
sp|BA.1.1|          TKCTLKSFTVEKGIYQTSNFRVQPTESIVRFPNITNLCPFDEVFNATKFASVYAWNRKRI
sp|B.1.617.2|       TKCTLKSFTVEKGIYQTSNFRVQPTESIVRFPNITNLCPFGEVFNATRFASVYAWNRKRI
sp|B.1.351|         TKCTLKSFTVEKGIYQTSNFRVQPTESIVRFPNITNLCPFGEVFNATRFASVYAWNRKRI
sp|B.1.1.7|         TKCTLKSFTVEKGIYQTSNFRVQPTESIVRFPNITNLCPFGEVFNATRFASVYAWNRKRI
sp|Wuhan-Hu-1|      TKCTLKSFTVEKGIYQTSNFRVQPTESIVRFPNITNLCPFGEVFNATRFASVYAWNRKRI
sp|p.1|             TKCTLKSFTVEKGIYQTSNFRVQPTESIVRFPNITNLCPFGEVFNATRFASVYAWNRKRI
sp|PanCoVac|        MFIFLLFLTAAKGIYQTSNFRVA----AVRFPNITNLCPFGEVFNATA----------AI


sp|229E|            ------------------------------------------------------------
sp|NL63|            ------------------------------------------------------------
sp|HKU1             SNCNFNLSTLLRLVHTDSFSCNNFDESKIYGSCFKSIVLDKFAIPNSRRSDLQLGSSG-F
sp|OC43|            SNCNFNMSSLMSFIQADSFTCNNIDAAKIYGMCFSSITIDKFAIPNRRKVDLQLGNLG-Y
sp|Tor2|            SNCVADYSVLYNSTFFSTFKCYGVSATKLNDLCFSNVYADSFVVKGDDVRQIAPGQTG-V
sp|BA.1.1|          SNCVADYSVLYNLAPFFTFKCYGVSPTKLNDLCFTNVYADSFVIRGDEVRQIAPGQTG-N
sp|B.1.617.2|       SNCVADYSVLYNSASFSTFKCYGVSPTKLNDLCFTNVYADSFVIRGDEVRQIAPGQTG-K
sp|B.1.351|         SNCVADYSVLYNSASFSTFKCYGVSPTKLNDLCFTNVYADSFVIRGDEVRQIAPGQTG-N
sp|B.1.1.7|         SNCVADYSVLYNSASFSTFKCYGVSPTKLNDLCFTNVYADSFVIRGDEVRQIAPGQTG-K
sp|Wuhan-Hu-1|      SNCVADYSVLYNSASFSTFKCYGVSPTKLNDLCFTNVYADSFVIRGDEVRQIAPGQTG-K
sp|p.1|             SNCVADYSVLYNSASFSTFKCYGVSPTKLNDLCFTNVYADSFVIRGDEVRQIAPGQTG-K
sp|PanCoVac|        SNCVADYSVLYNSA---------------------------------AVRQIAPGQTGAA
```

```
sp|229E|           -------LPVSIVSLPVYHKHTFIVLYVNFELRRGPGRCYNCRPAVINITLANFNET---
sp|NL63|           -------LPETYVALPIYYQHTDINFTAT---ASFGGSCYVCKPHQVNISLNG-------
sp|HKU1            LQSSNYKIDTTSSSCQLYYSLPAINVTI----NNYNPSSWNRRYGFNNF---------NL
sp|OC43|           LQSSNYRIDTTATSCQLYYNLPAANVSV----SRFNPSTWNKRFGFIEDSVFKPQPAGVL
sp|Tor2|           IADYNYKLPDDFMGCVLAWNTRNIDATS--------TGNYNYKYRYLRHGKLRPFERDI-
sp|BA.1.1|         IADYNYKLPDDFTGCVIAWNSNKLDSKV--------SGNYNYLYRLFRKSNLKPFERDI-
sp|B.1.617.2|      IADYNYKLPDDFTGCVIAWNSNNLDSKV--------GGNYNYRYRLFRKSNLKPFERDI-
sp|B.1.351|        IADYNYKLPDDFTGCVIAWNSNNLDSKV--------GGNYNYLYRLFRKSNLKPFERDI-
sp|B.1.1.7|        IADYNYKLPDDFTGCVIAWNSNNLDSKV--------GGNYNYRYRLFRKSNLKPFERDI-
sp|Wuhan-Hu-1|     IADYNYKLPDDFTGCVIAWNSNNLDSKV--------GGNYNYLYRLFRKSNLKPFERDI-
sp|p.1|            IADYNYKLPDDFTGCVIAWNSNNLDSKV--------GGNYNYLYRLFRKSNLKPFERDI-
sp|PanCoVac|       IADYNYKLPDDFA-----------------------------------------------

sp|229E|           -KGPLCVDTSHFTTQFVGVK-------F--DRWSASINTGNCPFSFGKV-----------
sp|NL63|           -NTSVCVRTSHFSIRYIYNRVKSGSPGD--SSWHIYLKSGTCPFSFSKL-----------
sp|HKU1            SSHSVVYSRYCFSVNNTF--CPCAKPSFASSCKSHKPPSASCPIGTNYRSCESTTVLDHT
sp|OC43|           TNHDVVYAQHCFKAPKNF--CPCS------SCSGKNNGIGTCPAGTNSLTCDNLCTL---
sp|Tor2|           ---------------------------------------SNVPFSPDGKPCTP-PALN-C
sp|BA.1.1|         ---------------------------------------STEIYQAGNKPCNGVAGFN-C
sp|B.1.617.2|      ---------------------------------------STEIYQAGSKPCNGVEGFN-C
sp|B.1.351|        ---------------------------------------STEIYQAGSTPCNGVKGFN-C
sp|B.1.1.7|        ---------------------------------------STEIYQAGSTPCNGVEGFN-C
sp|Wuhan-Hu-1|     ---------------------------------------STEIYQAGSTPCNGVEGFN-C
sp|p.1|            ---------------------------------------STEIYQAGSTPCNGVEGFN-C
sp|PanCoVac|       ------------------------------------------------------------

sp|229E|           -----------------------NNFVKFGSVCFSLKDIPGGCAMPIMANLVNHKSHNIG
sp|NL63|           -----------------------NNFQKFKTICFSTVEVPGSCNFPLEATWHYTSYTIVG
sp|HKU1            DWCRCSCLPDPITAYDPRSCSQKKSLVGVGEHCAGFGVDEEKCGVLD--GSYNVSCLCST
sp|OC43|           -------DPITLKAPDTYKCPQSKSLVGIGEHCSGLAVKSDYCG--------NNSCTCQP
sp|Tor2|           YW--------PLNDYGFYT------TTGIGYQ-------P-----------Y--------
sp|BA.1.1|         YF--------PLRSYSFRP------TYGVGHQ-------P-----------Y--------
sp|B.1.617.2|      YF--------PLQSYGFQP------TNGVGYQ-------P-----------Y--------
sp|B.1.351|        YF--------PLQSYGFQP------TYGVGYQ-------P-----------Y--------
sp|B.1.1.7|        YF--------PLQSYGFQP------TNGVGYQ-------P-----------Y--------
sp|Wuhan-Hu-1|     YF--------PLQSYGFQP------TNGVGYQ-------P-----------Y--------
sp|p.1|            YF--------PLQSYGFQP------TNGVGYQ-------P-----------Y--------
sp|PanCoVac|       ----------------------------AGYQ-------P-----------Y--------

sp|229E|           SLYVSWSDGDVITGVP-----------------KPVEGVSSFMNVTLNKCTKYNIYDVSG
sp|NL63|           ALYVTWSEGNSITGVP-----------------YPVSGIREFSNLVLNNCTKYNIYDYVG
sp|HKU1            DAFLGWSYDTCVSNNRCNIFSNFILNGINSGTTCSNDLLQPNTEVYTDVCVDYDLYGITG
sp|OC43|           QAFLGWSADSCLQGDKCNIFANFILHDVNNGLTCSTDLQKANTEIELGVCVNYDLYGISG
sp|Tor2|           ---------------RVVVL-SFELLN-APATVCGP---KLSTDLIKNQCVNFNFNGLTG
sp|BA.1.1|         ---------------RVVVL-SFELLH-APATVCGP---KKSTNLVKNKCVNFNFNGLKG
sp|B.1.617.2|      ---------------RVVVL-SFELLH-APATVCGP---KKSTNLVKNKCVNFNFNGLTG
sp|B.1.351|        ---------------RVVVL-SFELLH-APATVCGP---KKSTNLVKNKCVNFNFNGLTG
sp|B.1.1.7|        ---------------RVVVL-SFELLH-APATVCGP---KKSTNLVKNKCVNFNFNGLTG
sp|Wuhan-Hu-1|     ---------------RVVVL-SFELLH-APATVCGP---KKSTNLVKNKCVNFNFNGLTG
sp|p.1|            ---------------RVVVL-SFELLH-APATVCGP---KKSTNLVKNKCVNFNFNGLTG
sp|PanCoVac|       ---------------RVVVL-SFELLA---------------------ACVNFNFNGLTG

sp|229E|           VGVIRISNDTFLNGIT--YTSTSGNLLGFKDVTNGTIYSITPCNPPDQLVVYQQAVVGAM
sp|NL63|           TGIIRSSNQSLAGGIT--YVSNSGNLLGFKNVSTGNIFIVTPCNQPDQVAVYQQSIIGAM
sp|HKU1            QGIFKEVSAVYYNSWQNLLYDSNGNIIGFKDFVTNKTYNIFPCYAGRVSAAF--HQNASS
sp|OC43|           QGIFVEVNATYYNSWQNLLYDSNGNLYGFRDYITNRTFMIHSCYSGRVSAAY--HANSSE
sp|Tor2|           TGVLTPSSKR-FQPFQQFGRDVSDFTDSVRDPKTSEILDISPCSFGGVSVITPGTNASSE
sp|BA.1.1|         TGVLTESNKK-FLPFQQFGRDIADTTDAVRDPQTLEILDITPCSFGGVSVITPGTNTSNQ
sp|B.1.617.2|      TGVLTESNKK-FLPFQQFGRDIADTTDAVRDPQTLEILDITPCSFGGVSVITPGTNTSNQ
sp|B.1.351|        TGVLTESNKK-FLPFQQFGRDIADTTDAVRDPQTLEILDITPCSFGGVSVITPGTNTSNQ
sp|B.1.1.7|        TGVLTESNKK-FLPFQQFGRDIADTTDAVRDPQTLEILDITPCSFGGVSVITPGTNTSNQ
sp|Wuhan-Hu-1|     TGVLTESNKK-FLPFQQFGRDIADTTDAVRDPQTLEILDITPCSFGGVSVITPGTNTSNQ
sp|p.1|            TGVLTESNKK-FLPFQQFGRDIADTTDAVRDPQTLEILDITPCSFGGVSVITPGTNTSNQ
sp|PanCoVac|       TGVLTA------------------------------------------------------
```

```
sp|229E|            LSENF----TSYGFSNVV--E-MPKFFYASNGTY------NCT---DAVL--------TY
sp|NL63|            TAVNE----SRYGLQNLL--Q-LPNFYYVSNGGN------NCT---TAVM--------TY
sp|HKU1             LALLYRNLKCSYVLNNISLA----T-------QPYFDSYLGCVFNADNLTDYSVSSCALR
sp|OC43|            PALLFRNIKCNYVFNNSLTRQLQPI-------NYSFDSYLGCVVNAYNSTAISVQTCDLT
sp|Tor2|            VAVLYQDVNCTDVSTAIHADQLTPAWRIYSTGNNVFQTQAGCLIGAEHVD--TSYECDIP
sp|BA.1.1|          VAVLYQGVNCTEVPVAIHADQLTPTWRVYSTGSNVFQTRAGCLIGAEYVN--NSYECDIP
sp|B.1.617.2|       VAVLYQGVNCTEVPVAIHADQLTPTWRVYSTGSNVFQTRAGCLIGAEHVN--NSYECDIP
sp|B.1.351|         VAVLYQGVNCTEVPVAIHADQLTPTWRVYSTGSNVFQTRAGCLIGAEHVN--NSYECDIP
sp|B.1.1.7|         VAVLYQGVNCTEVPVAIHADQLTPTWRVYSTGSNVFQTRAGCLIGAEHVN--NSYECDIP
sp|Wuhan-Hu-1|      VAVLYQDVNCTEVPVAIHADQLTPTWRVYSTGSNVFQTRAGCLIGAEHVN--NSYECDIP
sp|p.1|             VAVLYQGVNCTEVPVAIHADQLTPTWRVYSTGSNVFQTRAGCLIGAEHVN--NSYECDIP
sp|PanCoVac|        ------------------------------------------------------------

sp|229E|            SSFGVCADGSIIAVQPRNVSYDSV----------------------SAIVTANLSIPSNW
sp|NL63|            SNFGICADGSLIPVRPRNSSDNGI----------------------SAIITANLSIPSNW
sp|HKU1             MGSGFCVDYNSPSFSSSRRKRRSISASYRFVTFEPFNVSFVNDSIESVGGLYEIKIPTNF
sp|OC43|            VGSGYCVDYFK-----NRRSRRAITTGYRFTNFEPFTVNSVNDSLEPVGGLYEIQIPSEF
sp|Tor2|            IGAGICASYHTVSL---LRSTSQKS------IVAYTMSLGADSS-IAYSNNTIAIPTNF
sp|BA.1.1|          IGAGICASYQTQTKSHRRARSVASQS------IIAYTMSLGAENS-VAYSNNSIAIPTNF
sp|B.1.617.2|       IGAGICASYQTQTNSRRRARSVASQS------IIAYTMSLGAENS-VAYSNNSIAIPTNF
sp|B.1.351|         IGAGICASYQTHTNSPWRARSVASQS------IIAYTMSLGVENS-VAYSNNSIAIPTNF
sp|B.1.1.7|         IGAGICASYQTQTNSPRRARSVASQS------IIAYTMSLGAENS-VAYSNNSIAIPTNF
sp|Wuhan-Hu-1|      IGAGICASYQTQTNSPRRARSVASQS------IIAYTMSLGAENS-VAYSNNSIAIPTNF
sp|p.1|             IGAGICASYQTQTNSPRRARSVASQS------IIAYTMSLGAENS-VAYSNNSIAIPTNF
sp|PanCoVac|        ------------------------------------------------------------

sp|229E|            TTSVQVEYLQITSTPIVVDCSTYVCNGNVRCVELLKQYTSACKTIEDALRNSAMLESADV
sp|NL63|            TTSVQVEYLQITSTPIVVDCATYVCNGNPRCKNLLKQYTSACKTIEDALRLSAHLETNDV
sp|HKU1             TIVGQEEFIQTNSPKVTIDCSLFVCSNYAACHDLLSEYGTFCDNINSILDEVNGLLDTTQ
sp|OC43|            TIGNMEEFIQTSSPKVTIDCAAFVCGDYAACKLQLVEYGSFCDNINAILTEVNELLDTTQ
sp|Tor2|            SISITTEVMPVSMAKTSVDCNMYICGDSTECANLLLQYGSFCTQLNRALSGIAAEQDRNT
sp|BA.1.1|          TISVTTEILPVSMTKTSVDCTMYICGDSTECSNLLLQYGSFCTQLKRALTGIAVEQDKNT
sp|B.1.617.2|       TISVTTEILPVSMTKTSVDCTMYICGDSTECSNLLLQYGSFCTQLNRALTGIAVEQDKNT
sp|B.1.351|         TISVTTEILPVSMTKTSVDCTMYICGDSTECSNLLLQYGSFCTQLNRALTGIAVEQDKNT
sp|B.1.1.7|         TISVTTEILPVSMTKTSVDCTMYICGDSTECSNLLLQYGSFCTQLNRALTGIAVEQDKNT
sp|Wuhan-Hu-1|      TISVTTEILPVSMTKTSVDCTMYICGDSTECSNLLLQYGSFCTQLNRALTGIAVEQDKNT
sp|p.1|             TISVTTEILPVSMTKTSVDCTMYICGDSTECSNLLLQYGSFCTQLNRALTGIAVEQDKNT
sp|PanCoVac|        -------------------------------ANLLLQYGSFCTQLNRALA----------

sp|229E|            SEMLTFDKKAFTL------ANVSSFGDYNLSSVIPSLPRSGSRVAGRSAIEDILFSKLVT
sp|NL63|            SSMLTFDSNAFSL------ANVTSFGDYNLSSVLPQRNIRSSRIAGRSALEDLLFSKVVT
sp|HKU1             LHVADTLMQGVTLSSNLNTNLHFDVDNINFKSLVGCLGPHCGS-SSRSFFEDLLFDKVKL
sp|OC43|            LQVANSLMNGVTLSTKLKDGVNFNVDDINFAPVLGCLGSECSKASSRSAIEDLLFDKVKL
sp|Tor2|            REVFAQVKQMY-KTPTLKY-----FGGFNFSQ----ILPDPLKPTKRSFIEDLLFNKVTL
sp|BA.1.1|          QEVFAQVKQIY-KTPPIKY-----FGGFNFSQ----ILPDPSKPSKRSFIEDLLFNKVTL
sp|B.1.617.2|       QEVFAQVKQIY-KTPPIKD-----FGGFNFSQ----ILPDPSKPSKRSFIEDLLFNKVTL
sp|B.1.351|         QEVFAQVKQIY-KTPPIKD-----FGGFNFSQ----ILPDPSKPSKRSFIEDLLFNKVTL
sp|B.1.1.7|         QEVFAQVKQIY-KTPPIKD-----FGGFNFSQ----ILPDPSKPSKRSFIEDLLFNKVTL
sp|Wuhan-Hu-1|      QEVFAQVKQIY-KTPPIKD-----FGGFNFSQ----ILPDPSKPSKRSFIEDLLFNKVTL
sp|p.1|             QEVFAQVKQIY-KTPPIKD-----FGGFNFSQ----ILPDPSKPSKRSFIEDLLFNKVTL
sp|PanCoVac|        -------------------------------------------------AEDLLFNKVTL

sp|229E|            SGLGTVDADYKKCTKGLSIADLACAQYYNGIMVLPGVADAERMAMYTGSLIGGIALGGLF
sp|NL63|            SGLGTVDVDYKSCTKGLSIADLACAQYYNGIMVLPGVADAERMAMYTGSLIGGMVLGGLT
sp|HKU1             SDVGFVE-AYNNCTGGSEIRDLLCVQSFNGIKVLPPILSESQISGYTTAATVAAMFPPWS
sp|OC43|            SDVGFVE-AYNNCTGGAEIRDLICVQSYKGIKVLPPLLSENQISGYTLAATSASLFPPWT
sp|Tor2|            ADAGFMK-QYGECLGDINARDLICAQKFNGLTVLPPLLTDDMIAAYTAALVSGTATAGWT
sp|BA.1.1|          ADAGFIK-QYGDCLGDIAARDLICAQKFKGLTVLPPLLTDEMIAQYTSALLAGTITSGWT
sp|B.1.617.2|       ADAGFIK-QYGDCLGDIAARDLICAQKFNGLTVLPPLLTDEMIAQYTSALLAGTITSGWT
sp|B.1.351|         ADAGFIK-QYGDCLGDIAARDLICAQKFNGLTVLPPLLTDEMIAQYTSALLAGTITSGWT
sp|B.1.1.7|         ADAGFIK-QYGDCLGDIAARDLICAQKFNGLTVLPPLLTDEMIAQYTSALLAGTITSGWT
sp|Wuhan-Hu-1|      ADAGFIK-QYGDCLGDIAARDLICAQKFNGLTVLPPLLTDEMIAQYTSALLAGTITSGWT
sp|p.1|             ADAGFIK-QYGDCLGDIAARDLICAQKFNGLTVLPPLLTDEMIAQYTSALLAGTITSGWT
sp|PanCoVac|        ADAGFA-----------AARDLICAQKFNGLTVLPPLLTD---------------AAGWT
```

```
sp|229E|          SAA----SIPFSLAIQSRLNYVALQTDVLQENQKILAASFNKAMTNIVDAFTGVNDAITQ
sp|NL63|          SAA----AIPFSLALQARLNYVALQTDVLQENQKILAASFNKAINNIVASFSSVNDAITQ
sp|HKU1           AAA----GIPFSLNVQYRINGLGVTMDVLNKNQKLIATAFNNALLSIQNGFSAT------
sp|OC43|          AAA----GVPFYLNVQYRINGLGVTMDVLSQNQKLIANAFNNALHAIQQGFDAT------
sp|Tor2|          FGAGAALQIPFAMQMAYRFNGIGVTQNVLYENQKQIANQFNKAISQIQESLTTT------
sp|BA.1.1|        FGAGAALQIPFAMQMAYRFNGIGVTQNVLYENQKLIANQFNSAIGKIQDSLSST------
sp|B.1.617.2|     FGAGAALQIPFAMQMAYRFNGIGVTQNVLYENQKLIANQFNSAIGKIQDSLSST------
sp|B.1.351|       FGAGAALQIPFAMQMAYRFNGIGVTQNVLYENQKLIANQFNSAIGKIQDSLSST------
sp|B.1.1.7|       FGAGAALQIPFAMQMAYRFNGIGVTQNVLYENQKLIANQFNSAIGKIQDSLSST------
sp|Wuhan-Hu-1|    FGAGAALQIPFAMQMAYRFNGIGVTQNVLYENQKLIANQFNSAIGKIQDSLSST------
sp|p.1|           FGAGAALQIPFAMQMAYRFNGIGVTQNVLYENQKLIANQFNSAIGKIQDSLSST------
sp|PanCoVac|      FGAGAALQIPFAMQMAYRFNGIGVTQNVLYENQKA-------------------------

sp|229E|          TSQALQTVATALNKIQDVVNQQGNSLNHLTSQLRQNFQAISSSIQAIYDRLDIIQADQQV
sp|NL63|          TAEAIHTVTIALNKIQDVVNQQGSALNHLTSQLRHNFQAISNSIQAIYDRLDSIQADQQV
sp|HKU1           --------NSALAKIQSVVNSNAQALNSLLQQLFNKFGAISSSLQEILSRLDALEAQVQI
sp|OC43|          --------NSALVKIQAVVNANSEALNNLLQQLSNRFGAISASLQEILSRLDALEAEAQI
sp|Tor2|          --------STALGKLQDVVNQNAQALNTLVKQLSSNFGAISSVLNDILSRLDKVEAEVQI
sp|BA.1.1|        --------ASALGKLQDVVNHNAQALNTLVKQLSSKFGAISSVLNDIFSRLDKVEAEVQI
sp|B.1.617.2|     --------ASALGKLQNVVNQNAQALNTLVKQLSSNFGAISSVLNDILSRLDKVEAEVQI
sp|B.1.351|       --------ASALGKLQDVVNQNAQALNTLVKQLSSNFGAISSVLNDILSRLDKVEAEVQI
sp|B.1.1.7|       --------ASALGKLQDVVNQNAQALNTLVKQLSSNFGAISSVLNDILSRLDKVEAEVQI
sp|Wuhan-Hu-1|    --------ASALGKLQDVVNQNAQALNTLVKQLSSNFGAISSVLNDILSRLDKVEAEVQI
sp|p.1|           --------ASALGKLQDVVNQNAQALNTLVKQLSSNFGAISSVLNDILSRLDKVEAEVQI
sp|PanCoVac|      ---------AALGKLQDVVNQNAQALNTLVKQLSSNFGAISSVLNDILSRLDKVEAEVQI

sp|229E|          DRLITGRLAALNVFVSHTLTKYTEVRASRQLAQQKVNECVKSQSKRYGFCGNGTHIFSLV
sp|NL63|          DRLITGRLAALNAFVSQVLNKYTEVRGSRRLAQQKINECVKSQSNRYGFCGNGTHIFSIV
sp|HKU1           DRLINGRLTALNAYVSQQLSDISLVKFGAALAMEKVNECVKSQSPRINFCGNGNHILSLV
sp|OC43|          DRLINGRLTALNAYVSQQLSDSTLVKFSAAQAMEKVNECVKSQSSRINFCGNGNHIISLV
sp|Tor2|          DRLITGRLQSLQTYVTQQLIRAAEIRASANLAATKMSECVLGQSKRVDFCGKGYHLMSFP
sp|BA.1.1|        DRLITGRLQSLQTYVTQQLIRAAEIRASANLAATKMSECVLGQSKRVDFCGKGYHLMSFP
sp|B.1.617.2|     DRLITGRLQSLQTYVTQQLIRAAEIRASANLAATKMSECVLGQSKRVDFCGKGYHLMSFP
sp|B.1.351|       DRLITGRLQSLQTYVTQQLIRAAEIRASANLAATKMSECVLGQSKRVDFCGKGYHLMSFP
sp|B.1.1.7|       DRLITGRLQSLQTYVTQQLIRAAEIRASANLAATKMSECVLGQSKRVDFCGKGYHLMSFP
sp|Wuhan-Hu-1|    DRLITGRLQSLQTYVTQQLIRAAEIRASANLAATKMSECVLGQSKRVDFCGKGYHLMSFP
sp|p.1|           DRLITGRLQSLQTYVTQQLIRAAEIRASANLAATKMSECVLGQSKRVDFCGKGYHLMSFP
sp|PanCoVac|      DRLITGRLQSLQTYVTQQLIRAAEIRASANLAATKMSECVLGQSKRVDFCGKGYHLMSFP

sp|229E|          NA-APEGLVFLHTVLLPTQYKDVEAWSGLCVDGINGYVLRQPNLALYKEGNYYRITSRIM
sp|NL63|          NS-APDGLLFLHTVLLPTDYKNVKAWSGICVDGIYGYVLRQPNLVLYSDNGVFRVTSRIM
sp|HKU1           QN-APYGLLFMHFSYKPISFKTVLVSPGLCISGDVGIAPKQGYF--IKHNDHWMFTGSSY
sp|OC43|          QN-APYGLYFIHFNYVPTKYVTAKVSPGLCIAGNRGIAPKSGYF--VNVNNTWMYTGSGY
sp|Tor2|          QA-APHGVVFLHVTYVPSQERNFTTAPAICHEGK-AYFPREGVF--VFNGTSWFITQRNF
sp|BA.1.1|        QS-APHGVVFLHVTYVPAQEKNFTTAPAICHDGK-AHFPREGVF--VSNGTHWFVTQRNF
sp|B.1.617.2|     QS-APHGVVFLHVTYVPAQEKNFTTAPAICHDGK-AHFPREGVF--VSNGTHWFVTQRNF
sp|B.1.351|       QS-APHGVVFLHVTYVPAQEKNFTTAPAICHDGK-AHFPREGVF--VSNGTHWFVTQRNF
sp|B.1.1.7|       QS-APHGVVFLHVTYVPAQEKNFTTAPAICHDGK-AHFPREGVF--VSNGTHWFVTQRNF
sp|Wuhan-Hu-1|    QS-APHGVVFLHVTYVPAQEKNFTTAPAICHDGK-AHFPREGVF--VSNGTHWFVTQRNF
sp|p.1|           QS-APHGVVFLHVTYVPAQEKNFTTAPAICHDGK-AHFPREGVF--VSNGTHWFVTQRNF
sp|PanCoVac|      QAAAPHGVVFLHVTYVPA------------------------------------------

sp|229E|          FEPRIPTIADFVQIENCNVTFVNI-SRSELQTIVPEYIDVNKTLQELSYKLPNYTVPDLV
sp|NL63|          FQPRLPVLSDFVQIYNCNVTFVNI-SRVELHTVIPDYVDVNKTLQEFAQNLPKYVKPNFD
sp|HKU1           YYPEPISDKNVVFMNTCSVNFTKA-PLVYLNHSVPKLSDFESELSHWF---KNQTSIAP-
sp|OC43|          YYPEPITENNVVVMSTCAVNYTKA-PYVMLNTSIPNLPDFKEELDQWF---KNQTSVAP-
sp|Tor2|          FSPQIITTDNTFVSGNCDVVIGII-NNTVYDPLQPELDSFKEELDKYF---KNHTSPDV-
sp|BA.1.1|        YEPQIITTDNTFVSGNCDVVIGIV-NNTVYDPLQPELDSFKEELDKYF---KNHTSPDV-
sp|B.1.617.2|     YEPQIITTDNTFVSGNCDVVIGIV-NNTVYDPLQPELDSFKEELDKYF---KNHTSPDV-
sp|B.1.351|       YEPQIITTDNTFVSGNCDVVIGIV-NNTVYDPLQPELDSFKEELDKYF---KNHTSPDV-
sp|B.1.1.7|       YEPQIITTDNTFVSGNCDVVIGIV-NNTVYDPLQPELDSFKEELDKYF---KNHTSPDV-
sp|Wuhan-Hu-1|    YEPQIITTDNTFVSGNCDVVIGIV-NNTVYDPLQPELDSFKEELDKYF---KNHTSPDV-
sp|p.1|           YEPQIITTDNTFVSGNCDVVIGIV-NNTVYDPLQPELDSFKEELDKYF---KNHTSPDV-
sp|PanCoVac|      -APQIITTDNTFVSGNCDVVIGIAANNTVYDPLQPELDSFKEELDKYF---KNHTSPDV-
```

```
sp|229E|           VEQYNQTILNLTSEISTLENKSAELNYTVQKLQTLIDNINSTLVDLKWLNRVETYIKWPW
sp|NL63|           LTPFNLTYLNLSSELKQLEAKTASLFQTTVELQGLIDQINSTYVDLKLLNRFENYIKWPW
sp|HKU1            ---------NLTLNLHTINATFLDLYYEMNLIQESIKSLNNSYINLKDIGTYEMYVKWPW
sp|OC43|           ---------DLSL--DYINVTFLDLQVEMNRLQEAIKVLNHSYINLKDIGTYEYYVKWPW
sp|Tor2|           ---------DLG-DISGINASVVNIQKEIDRLNEVAKNLNESLIDLQELGKYEQYIKWPW
sp|BA.1.1|         ---------DLG-DISGINASVVNIQKEIDRLNEVAKNLNESLIDLQELGKYEQYIKWPW
sp|B.1.617.2|      ---------DLG-DISGINASVVNIQKEIDRLNEVAKNLNESLIDLQELGKYEQYIKWPW
sp|B.1.351|        ---------DLG-DISGINASVVNIQKEIDRLNEVAKNLNESLIDLQELGKYEQYIKWPW
sp|B.1.1.7|        ---------DLG-DISGINASVVNIQKEIDRLNEVAKNLNESLIDLQELGKYEQYIKWPW
sp|Wuhan-Hu-1|     ---------DLG-DISGINASVVNIQKEIDRLNEVAKNLNESLIDLQELGKYEQYIKWPW
sp|p.1|            ---------DLG-DISGINASVVNIQKEIDRLNEVAKNLNESLIDLQELGKYEQYIKWPW
sp|PanCoVac|       ---------DLG-DISGINASVVNIQKEIDRLNEVAKNLNESLIDLQELGKYEQYIKWPW

sp|229E|           WV-WLCISVVLIFVVSMLLLCCCSTGCCGFFSCFASSIRGCCE--STK---LPYYDVEKI
sp|NL63|           WV-WLIISVVFVVLLSLLVFCCLSTGCCGCCNCLTSSMRGCCDCGSTK---LPYYEFEKV
sp|HKU1            YV-WLLISFSFIIFLVLLFFICCCTGCGSACF---SKCHNCCDEYGGHH--DFVIKTSH-
sp|OC43|           YV-WLLICLAGVAMLVLLFFICCCTGCGTSCF---KKCGGCCDDYTGYQ--ELVIKTSH-
sp|Tor2|           Y-VWLGFIAGLIAIVMVTILLCCMTSCCS-CLKGACSCGSCCKFDE--DDSEPVLKGVKL
sp|BA.1.1|         Y-IWLGFIAGLIAIVMVTIMLCCMTSCCS-CLKGCCSCGSCCKFDE--DDSEPVLKGVKL
sp|B.1.617.2|      Y-IWLGFIAGLIAIVMVTIMLCCMTSCCS-CLKGCCSCGSCCKFDE--DDSEPVLKGVKL
sp|B.1.351|        Y-IWLGFIAGLIAIVMVTIMLCCMTSCCS-CLKGCCSCGSCCKFDE--DDSEPVLKGVKL
sp|B.1.1.7|        Y-IWLGFIAGLIAIVMVTIMLCCMTSCCS-CLKGCCSCGSCCKFDE--DDSEPVLKGVKL
sp|Wuhan-Hu-1|     Y-IWLGFIAGLIAIVMVTIMLCCMTSCCS-CLKGCCSCGSCCKFDE--DDSEPVLKGVKL
sp|p.1|            Y-IWLGFIAGLIAIVMVTIMLCCMTSCCS-CLKGCCSCGSCCKFDE--DDSEPVLKGVKL
sp|PanCoVac|       YAAWLGFIAGLIAIVMVTIA--------------ACSCGSCCKFDEAADDSEPVLKGVKL

sp|229E|           H-------IQMFL---KLVDDHALIVNVLLWCVVLIVILLVCITIIKLIKLCFTCHMFCN
sp|NL63|           H-------VQMFL---RLIDDNGIVLNSILWLLVMIFFFVLAMTFIKLIQLCFTCHYFFS
sp|HKU1            --------DDM--VDLFFNDTAWYIGQILVLVLFCLISLIFVVAFLATIKLCMQLCGFCN
sp|OC43|           --------DDMFMADAYLADTVWYVGQIIFIVAICLLVTIVVVAFLATFKLCIQLCGMCN
sp|Tor2|           HYT-------MY--SFVSEETGTLIVNSVLLFLAFVVFLLVTLAILTALRLCAYCCNIVN
sp|BA.1.1|         HYT-------MY--SFVSEEIGTLIVNSVLLFLAFVVFLLVTLAILTALRLCAYCCNIVN
sp|B.1.617.2|      HYT-------MY--SFVSEETGTLIVNSVLLFLAFVVFLLVTLAILTALRLCAYCCNIVN
sp|B.1.351|        HYT-------MY--SFVSEETGTLIVNSVLLFLAFVVFLLVTLAILTALRLCAYCCNIVN
sp|B.1.1.7|        HYT-------MY--SFVSEETGTLIVNSVLLFLAFVVFLLVTLAILTALRLCAYCCNIVN
sp|Wuhan-Hu-1|     HYT-------MY--SFVSEETGTLIVNSVLLFLAFVVFLLVTLAILTALRLCAYCCNIVN
sp|p.1|            HYT-------MY--SFVSEETGTLIVNSVLLFLAFVVFLLVTLAILTALRLCAYCCNIVN
sp|PanCoVac|       HYTRGRKRRSMY--SFVSEETGTLIVNSVLLFLAFVVFLLVTLAILTALRLCAYCCNIVN

sp|229E|           RTVYGPIKNVYH----IYQSYMHIDPFPKRVIDFMSND--------NCTGDIVTHLKNWN
sp|NL63|           RTLYQPVYKIFL----AYQDYMQIAPVPAEVLNVMFLR--------LIDDN---------
sp|HKU1            FFIISPSAYVYKRGMQLYKSYSEQVIPPTSDYLIMNKS----FFPQFTSDQAVTFLKEWN
sp|OC43|           TLVLSPSIYVFNRGRQFYEFYNDIKPPVLDVDDVMSSKPTPAPVYIWTADEAIKFLKEWN
sp|Tor2|           VSLVKPTVYVYSRVKNLNSSEGVPD---L--LV-MA------DNGTITVEELKQLLEQWN
sp|BA.1.1|         VSLVKPSFYVYSRVKNLNSSRV-PD---L--LVMAG------SNGTITVEELKKLLEEWN
sp|B.1.617.2|      VSLVKPSFYVYSRVKNLNSSRV-PD---L--LVMAD------SNGTITVEELKKLLEQWN
sp|B.1.351|        VSLVKPSFYVYSRVKNLNSSRV-LD---L--LVMAD------SNGTITVEELKKLLEQWN
sp|B.1.1.7|        VSLVKPSFYVYSRVKNLNSSRV-PD---L--LVMAD------SNGTITVEELKKLLEQWN
sp|Wuhan-Hu-1|     VSLVKPSFYVYSRVKNLNSSRV-PD---L--LVMAD------SNGTITVEELKKLLEQWN
sp|p.1|            VSLVKPSFYVYSRVKNLNSSRV-PD---L--LVMAD------SNGTITVEELKKLLEQWN
sp|PanCoVac|       VSLVKPAAYVYSRVKNLNSSRGRKRRS---------------------------------

sp|229E|           FGWNVILTIFIVILQFGHYKYSRLFYGLKM-LVLWLLWPLVLALSIFDTWANWDSNWAFV
sp|NL63|           ----------------------------GI-VLNSILWLLVMIF----------------
sp|HKU1            FSLGVILLFITIILQFGYTSRSMFVYFIKM-IILWLMWPLTITLTIFNC--FYALNNAFL
sp|OC43|           FSLGIILLFITIILQYGYTSRSMFVYVIKM-IILWLMWPITIILTTFNC--VYALNNVYL
sp|Tor2|           LVIGFLFLAWIMLLQFAYSNRNRFLYIIKL-VFLWLLWPVTLACFVLAA--VYRINWVTG
sp|BA.1.1|         LVIGFLFLTWICLLQFAYANRNRFLYIIKL-IFLWLLWPVTLTCFVLAA--VYRINWITG
sp|B.1.617.2|      LVIGFLFLTWICLLQFAYANRNRFLYIIKL-IFLWLLWPVTLACFVLAA--VYRINWITG
sp|B.1.351|        LVIGFLFLTWICLLQFAYANRNRFLYIIKL-IFLWLLWPVTLACFVLAA--VYRINWITG
sp|B.1.1.7|        LVIGFLFLTWICLLQFAYANRNRFLYIIKL-IFLWLLWPVTLACFVLAA--VYRINWITG
sp|Wuhan-Hu-1|     LVIGFLFLTWICLLQFAYANRNRFLYIIKL-IFLWLLWPVTLACFVLAA--VYRINWITG
sp|p.1|            LVIGFLFLTWICLLQFAYANRNRFLYIIKL-IFLWLLWPVTLACFVLAA--VYRINWITG
sp|PanCoVac|       -------------------NRNRFLYIIKLAAFLWLLWPVTLACFVLAA--VYRINWAA-
```

```
sp|229E|          AFSFLMAVSTLVMWVMYFANSFRLFRRARTFWAWNPEVNAITVTTVLGQTYYQPIQQAPT
sp|NL63|          --FFVLAMTFIKLIQLCFTC-----------------------HYFFSRTLYQPVYKIFL
sp|HKU1           AFSIVFTIISIVIWILYFVNSIRLFIRTGSWWSFNPETNNLMCIDMKGKMFVRPVIEDYH
sp|OC43|          GLSIVFTIVAIIMWIVYFVNSIRLFIRTGSFWSFNPETNNLMCIDMKGTMYVRPIIEDYH
sp|Tor2|          GIAIAMACIVGLMWLSYFVASFRLFARTRSMWSFNPETNILLNVPLRGTIVTRPLMESEL
sp|BA.1.1|        GIAIAMACLVGLMWLSYFIASFRLFARTRSMWSFNPETNILLNVPLHGTILTRPLLESEL
sp|B.1.617.2|     GIATAMACLVGLMWLSYFIASFRLFARTRSMWSFNPETNILLNVPLHGTILTRPLLESEL
sp|B.1.351|       GIAIAMACLVGLMWLSYFIASFRLFARTRSMWSFNPETNILLNVPLHGTILTRPLLESEL
sp|B.1.1.7|       GIAIAMACLVGLMWLSYFIASFRLFARTRSMWSFNPETNILLNVPLHGTILTRPLLESEL
sp|Wuhan-Hu-1|    GIAIAMACLVGLMWLSYFIASFRLFARTRSMWSFNPETNILLNVPLHGTILTRPLLESEL
sp|p.1|           GIAIAMACLVGLMWLSYFIASFRLFARTRSMWSFNPETNILLNVPLHGTILTRPLLESEL
sp|PanCoVac|      -------------------ASFRLFARTRSMWSFNPETNILLNVPLAA--------ESEL

sp|229E|          GITVTLLSGVLYVDGHRLASGVQVHNLPEYMTVAVPSTTIIYSRVGRSVNSQNSTGWVFY
sp|NL63|          -------------------------AYQDYMQIAP-------------------------
sp|HKU1           TLTATVIRGHLYIQGVKLGTGYTLSDLPVYVTVAKVQVLC-TYKRAFLDKLDVNSGFAVF
sp|OC43|          TLTVTIIRGHLYIQGIKLGIGYSLADLPAYMSVAKVTHLC-TYKRGFLDRISDTSGFAVY
sp|Tor2|          VIGAVIIRGHLRMAGHSLGR-CDIKDLPKEITVATSRTLS-YYKLGASQRVGTDSGFAAY
sp|BA.1.1|        VIGAVILRGHLRIAGHHLGR-CDIKDLPKEITVATSRTLS-YYKLGASQRVAGDSGFAAY
sp|B.1.617.2|     VIGAVILRGHLRIAGHHLGR-CDIKDLPKEITVATSRTLS-YYKLGASQRVAGDSGFAAY
sp|B.1.351|       VIGAVILRGHLRIAGHHLGR-CDIKDLPKEITVATSRTLS-YYKLGASQRVAGDSGFAAY
sp|B.1.1.7|       VIGAVILRGHLRIAGHHLGR-CDIKDLPKEITVATSRTLS-YYKLGASQRVAGDSGFAAY
sp|Wuhan-Hu-1|    VIGAVILRGHLRIAGHHLGR-CDIKDLPKEITVATSRTLS-YYKLGASQRVAGDSGFAAY
sp|p.1|           VIGAVILRGHLRIAGHHLGR-CDIKDLPKEITVATSRTLS-YYKLGASQRVAGDSGFAAY
sp|PanCoVac|      VIGAVIA---------ALGR-CDIKDLPKEITVATSRTLS-YYKLGASQRVA---------

sp|229E|          VRVKHGDFSAVSSPMSNMTENERLL---------------------HFFMATVKWADASE
sp|NL63|          --------------------VPAEV---------------------LNVMASVNWADDRA
sp|HKU1           VKSKVGNYRLPSS-KPSGMDTALLR--AMSYTPGHYAGSRSSSGNRSGILKKTSWADQSE
sp|OC43|          VKFKVGNYRLPSTQKGSGMDTALLRNNIMSFTPGKQSSSRASSGNRSGNG-ILKWADQSD
sp|Tor2|          NRYRIGNYKLNTDHAGSNDNIALLVQMS---DNGPQSNQRSAP--------RITFGGPTD
sp|BA.1.1|        SRYRIGNYKLNTDHSSSSDNIALLVQMS---DNGPQ-NQRNAL--------RITFGGPSD
sp|B.1.617.2|     SRYRIGNYKLNTDHSSSSDNIALLVQMS---DNGPQ-NQRNAP--------RITFGGPSD
sp|B.1.351|       SRYRIGNYKLNTDHSSSSDNIALLVQMS---DNGPQ-NQRNAP--------RITFGGPSD
sp|B.1.1.7|       SRYRIGNYKLNTDHSSSSDNIALLVQMS---DNGPQ-NQRNAP--------RITFGGPSD
sp|Wuhan-Hu-1|    SRYRIGNYKLNTDHSSSSDNIALLVQMS---DNGPQ-NQRNAP--------RITFGGPSD
sp|p.1|           SRYRIGNYKLNTDHSSSSDNIALLVQMS---DNGPQ-NQRNAP--------RITFGGPSD
sp|PanCoVac|      ARYRIGNYKLNTDH----------------------------------------------

sp|229E|          PQ-----RGRQGR----------------IPYSLYSPLLVDS-EQPWKVIPRNLVPINKK
sp|NL63|          A--------RKKF----------------PPPSFYMPLLVSSDKAPYRVIPRNLVPIGKG
sp|HKU1           RNYQTFNRGRKTQPKFTVST--QPQGNTIPHYSWFSGITQFQKGRDFKFSDGQGVPIAFG
sp|OC43|          QFRNFQTRGRRAQPKQTATSQQPSGGNVVPHYSWFSGITQFQKGKEFEFAEGQGVPIAPG
sp|Tor2|          STDNNQNGGRNGA----RPKQRRPQGLPNNTASWFTALTQHGK-EELRFPRGQGVPINTN
sp|BA.1.1|        STGSNQNG---GA----RSKQRRPQGLPNNTASWFTALTQHGK-EDLKFPRGQGVPINTN
sp|B.1.617.2|     STGSNQNGERSGA----RSKQRRPQGLPNNTASWFTALTQHGK-EGLKFPRGQGVPINTN
sp|B.1.351|       STGSNQNGERSGA----RSKQRRPQGLPNNTASWFTALTQHGK-EDLKFPRGQGVPINTN
sp|B.1.1.7|       STGSNQNGERSGA----RSKQRRPQGLPNNTASWFTALTQHGK-EDLKFPRGQGVPINTN
sp|Wuhan-Hu-1|    STGSNQNGERSGA----RSKQRRPQGLPNNTASWFTALTQHGK-EDLKFPRGQGVPINTN
sp|p.1|           STGSNQNGERSGA----RSKQRRPQGLPNNTASWFTALTQHGK-EDLKFPRGQGVPINTN
sp|PanCoVac|      ------------RGRKRRSKQRRPQGLPNNTASWFTALTQHGK-E-AAFPRGQGVPINTN

sp|229E|          -DKNKLIGYWNVQ--KRFRTRKGKRVDLSPKLHFYYLGTGPHKDAKFRERVEGVVWVAVD
sp|NL63|          -NKDEQIGYWNVQ--ERWRMRRGQRVDLPPKVHFYYLGTGPHKDLKFRQRSDGVVWVAKE
sp|HKU1           VPPSEAKGYWYRHSRRSFKTADGQQKQLLPRWYFYYLGTGPYANASYGESLEGVFWVANH
sp|OC43|          VPATEAKGYWYKHNRRSFKTADGNQRQLLPRWYFYYLGTGPHAKDQYGTDINGVYWVASN
sp|Tor2|          SGPDDQIGYYRRAT-RRVRGGDGKMKELSPRWYFYYLGTGPEASLPYGANKEGIVWVATE
sp|BA.1.1|        SSPDDQIGYYRRAT-RRIRGGDGKMKDLSPRWYFYYLGTGPEAGLPYGANKDGIIWVATE
sp|B.1.617.2|     SSPDDQIGYYRRAT-RRIRGGDGKMKDLSPRWYFYYLGTGPEAGLPYGANKDGIIWVATE
sp|B.1.351|       SSPDDQIGYYRRAT-RRIRGGDGKMKDLSPRWYFYYLGTGPEAGLPYGANKDGIIWVATE
sp|B.1.1.7|       SSPDDQIGYYRRAT-RRIRGGDGKMKDLSPRWYFYYLGTGPEAGLPYGANKDGIIWVATE
sp|Wuhan-Hu-1|    SSPDDQIGYYRRAT-RRIRGGDGKMKDLSPRWYFYYLGTGPEAGLPYGANKDGIIWVATE
sp|p.1|           SSPDDQIGYYRRAT-RRIRGGDGKMKDLSPRWYFYYLGTGPEAGLPYGANKDGIIWVATE
sp|PanCoVac|      SA------------------------ALSPRWYFYYLGTGPEA-----------------
```

```
sp|229E|          GAKTEPTG-YGVRRKNSEPEIPHFNQKLPNGVTVVE--EPDSRA-----PSRSQSRS--Q
sp|NL63|          GAKTVNTS-LGNRKRNQKPLEPKFSIALPPELSVVE--FEDRSNNSSRASSRSSTRN--N
sp|HKU1           QADTSTPSDVSSRDPTTQEAIP---TRFPPGTILPQGYYVEGSGRSASN-SRPGSRS--Q
sp|OC43|          QADVNTPADIVDRDPSSDEAIP---TRFPPGTVLPQGYYIEGSGRSAPN-SRSTSRT--S
sp|Tor2|          GALNTPKDHIGTRNPNNNAATV---LQLPQGTTLPKGFYAEGSRGGSQASSRSSSRSRGN
sp|BA.1.1|        GALNTPKDHIGTRNPANNAAIV---LQLPQGTTLPKGFYAEGSRGGSQASSRSSSRSRNS
sp|B.1.617.2|     GALNTPKDHIGTRNPANNAAIV---LQLPQGTTLPKGFYAEGSRGGSQASSRSSSRSRNS
sp|B.1.351|       GALNTPKDHIGTRNPANNAAIV---LQLPQGTTLPKGFYAEGSRGGSQASSRSSSRSRNS
sp|B.1.1.7|       GALNTPKDHIGTRNPANNAAIV---LQLPQGTTLPKGFYAEGSRGGSQASSRSSSRSRNS
sp|Wuhan-Hu-1|    GALNTPKDHIGTRNPANNAAIV---LQLPQGTTLPKGFYAEGSRGGSQASSRSSSRSRNS
sp|p.1|           GALNTPKDHIGTRNPANNAAIV---LQLPQGTTLPKGFYAEGSRGGSQASSRSSSRSRNS
sp|PanCoVac|      -------------------AAV---LQLPQGTTLPKGFYAEGSRGGSQASSRSSSRSRA-

sp|229E|          SRSRGE-SKSQSRNPSSDRNHNSQDDIMKAVAAALKSLGFDKPQEKDKKSAKTGTPKPSR
sp|NL63|          SRDSSR-STSRQQSRTRSDSNQSSSDLVAAVTLALKNLGFDNQSKSPSSS---GTSTPKK
sp|HKU1           SRGPNTRSLSRSNSNFRHSDSIVKPDM----ADEIANLVLAKLGKDS---------KPQQ
sp|OC43|          SRASSAGSRSRANSGNRTPTSGVTPDM----ADQIASLVLAKLGKDAT--------KPQQ
sp|Tor2|          SRNSTPG-SSRGNSPARMASGGGE--------TALALLLLDRLNQLESK-VS-G-KGQQQ
sp|BA.1.1|        SRNSTPG-SSKRTSPARMAGNGGD--------AALALLLLDRLNQLESK-MS-G-KGQQQ
sp|B.1.617.2|     SRNSTPG-SSMGTSPARMAGNGCD--------AALALLLLDRLNQLESK-MS-G-KGQQQ
sp|B.1.351|       SRNSTPG-SSRGISPARMAGNGGD--------AALALLLLDRLNQLESK-MS-G-KGQQQ
sp|B.1.1.7|       SRNSTPG-SSRGISPARMAGNGGD--------AALALLLLDRLNQLESK-MS-G-KGQQQ
sp|Wuhan-Hu-1|    SRNSTPG-SSRGTSPARMAGNGGD--------AALALLLLDRLNQLESK-MS-G-KGQQQ
sp|p.1|           SRNSTPG-SSKRTSPARMAGNGGD--------AALALLLLDRLNQLESK-MS-G-KGQQQ
sp|PanCoVac|      --------------------------------AALALLLLDRLNQLESKAAS-G-KGQQQ

sp|229E|          NQSPASSQSAAKILARSQSSETKEQKHEMQKPRWKRQPNDDVTSNVTQCFGPRDLDHNFG
sp|NL63|          PNKPL-------------SQPRADKPSQLKKPRWKRVPTRE--ENVIQCFGPRDFNHNMG
sp|HKU1           V---------------TKQNAKEIRHKILTKPRQKRTPNK--HCNVQQCFGKRGPSQNFG
sp|OC43|          V---------------TKHTAKEVRQKILNKPRQKRSPNK--QCTVQQCFGKRGPNQNFG
sp|Tor2|          Q---------------GQTVTKKSAAEASKKPRQKRTATK--QYNVTQAFGRRGPEQTQG
sp|BA.1.1|        Q---------------GQTVTKKSAAEASKKPRQKRTATK--AYNVTQAFGRRGPEQTQG
sp|B.1.617.2|     Q---------------GQTVTKKSAAEASKKPRQKRTATK--AYNVTQAFGRRGPEQTQG
sp|B.1.351|       Q---------------GQTVTKKSAAEASKKPRQKRTATK--AYNVTQAFGRRGPEQTQG
sp|B.1.1.7|       Q---------------GQTVTKKSAAEASKKPRQKRTATK--AYNVTQAFGRRGPEQTQG
sp|Wuhan-Hu-1|    Q---------------GQTVTKKSAAEASKKPRQKRTATK--AYNVTQAFGRRGPEQTQG
sp|p.1|           Q---------------GQTVTKKSAAEASKKPRQKRTATK--AYNVTQAFGRRGPEQTQG
sp|PanCoVac|      Q---------------GQTVTKKSAAEASKKPRQKRTATKA-AYNVTQAFGRRGPEQTQG

sp|229E|          S----AGVVANGVKAKGYPQFAELVPSTAAMLFDSHIVSKESG----------NTVVLTF
sp|NL63|          D----SDLVQNGVDAKGFPQLAELIPNQAALFFDSEVSTDEVG----------DNVQITY
sp|HKU1           N----AEMLKLGTNDPQFPILAELAPTPGAFFFGSKLELVKRE---SEADSPVKDVFELR
sp|OC43|          G----GEMLKLGTSDPQFPILAELAPTAGAFFFGSKLELAKVQNLSGNPDEPQKDVYELR
sp|Tor2|          NFGDQD-LIRQGTDYKHWPQIAQFAPSASAFFGMSRIGMEVTP----------SGTWLTY
sp|BA.1.1|        NFGDQE-LIRQGTDYKHWPQIAQFAPSASAFFGMSRIGMEVTP----------SGTWLTY
sp|B.1.617.2|     NFGDQE-LIRQGTDYKHWPQIAQFAPSASAFFGMSRIGMEVTP----------SGTWLTY
sp|B.1.351|       NFGDQE-LIRQGTDYKHWPQIAQFAPSASAFFGMSRIGMEVTP----------SGTWLTY
sp|B.1.1.7|       NFGDQE-LIRQGTDYKHWPQIAQFAPSASAFFGMSRIGMEVTP----------SGTWLTY
sp|Wuhan-Hu-1|    NFGDQE-LIRQGTDYKHWPQIAQFAPSASAFFGMSRIGMEVTP----------SGTWLTY
sp|p.1|           NFGDQE-LTRQGTDYKHWPQIAQFAPSASAFFGMSRIGMEVTP----------SGTWLTY
sp|PanCoVac|      NFGDQAALIRQGTDYKHWPQIAQFAPSASAFFGMSRIGMEVTP----------SGTWLTY

sp|229E|          T-TRVTVPKDHPHLGKFL-------EELNAFTREMQQQPLLNPS--ALEFNP--------
sp|NL63|          T-YKMLVAKDNKNLPKFI-------EQISAFTKPSSIKEMQSQS--SHVAQN--------
sp|HKU1           YSGSIRF---DSTLPGFETIMKVLKENLDAYVNSNQNT-------VSGSLSPKPQRKRGV
sp|OC43|          YNGAIRF---DSTLSGFETIMKVLSENLNAYQQQDG----------MMNMSPKPQRQRGH
sp|Tor2|          H-GAIKL---DDKDPQFKDNVILLNKHIDAYKTFPPTEPKKDKKKKTDEAQPLPQRQKKQ
sp|BA.1.1|        T-GAIKL---DDKDPNFKDQVILLNKHIDAYKTFPPTEPKKDKKKKADETQALPQRQKKQ
sp|B.1.617.2|     T-GAIKL---DDKDPNFKDQVILLNKHIDAYKTFPPTEPKKDKKKKAYETQALPQRQKKQ
sp|B.1.351|       T-GAIKL---DDKDPNFKDQVILLNKHIDAYKTFPPTEPKKDKKKKADETQALPQRQKKQ
sp|B.1.1.7|       T-GAIKL---DDKDPNFKDQVILLNKHIDAYKTFPPTEPKKDKKKKADETQALPQRQKKQ
sp|Wuhan-Hu-1|    T-GAIKL---DDKDPNFKDQVILLNKHIDAYKTFPPTEPKKDKKKKADETQALPQRQKKQ
sp|p.1|           T-GAIKL---DDKDPNFKDQVILLNKHIDAYKTFPPTEPKKDKKKKADETQALPQRQKKQ
sp|PanCoVac|      AAGAIKL---DDKDP-----VILLNKHIDAYKTFPPTEPKKDKKK---------------
```

```
sp|229E|          -----SQTSPATVEPV-RDEVSIETDIIDEVN-----------------
sp|NL63|          ---TVLNASIPESKPL-ADDDSAIIEIVNEVLH----------------
sp|HKU1           KQSPELFDSLNLSA----DTQHISNDFTPEDHSLLATLDDPYVEDSVA-
sp|OC43|          KNGQGENDNISVAVPKSRVQQNKSIELTAEDISLLKKMDEPFTEDTSEI
sp|Tor2|          PTVT-------LL-PA-ADMDDFSR-------QLQNSMSGASADSTQA-
sp|BA.1.1|        QTVT-------LL-PA-ADLDDFSK-------QLQQSMSSADSTQA---
sp|B.1.617.2|     QTVT-------LL-PA-ADLDDFSK-------QLQQSMSSADSTQA---
sp|B.1.351|       QTVT-------LL-PA-ADLDDFSK-------QLQQSMSSADSTQA---
sp|B.1.1.7|       QTVT-------LL-PA-ADLDDFSK-------QLQQSMSSADSTQA---
sp|Wuhan-Hu-1|    QTVT-------LL-PA-ADLDDFSK-------QLQQSMSSADSTQA---
sp|p.1|           QTVT-------LL-PA-ADLDDFSK-------QLQQSMSSADSTQA---
sp|PanCoVac|      -------------------------------------------------
```

SUPPLEMENTARY FIGURE 1 | PanCoVac amino acids sequence. Alignment of PanCoVac with the structural proteins of human coronavirus 229E (GenBank: AB691767.2), human coronavirus NL63 (GenBank: JX504050.1), human coronavirus HKU1 (GenBank: KF686346.1), human coronavirus OC43 (GenBank: MZ450972.1), SARS-CoV-1 isolate Tor 2 (GenBank: JX163928.1), SARS-CoV-2 variant Omicron/BA.1.1 (GenBank: OQ899398.1), SARS-CoV-2 variant Delta/B.1.617.2 (GenBank: OQ906634.1), SARS-CoV-2 variant Beta/B.1.351 (GenBank: OX020113.1), SARS-CoV-2 variant Alpha/B.1.1.7 (GenBank: OX001768.1), SARS-CoV-2 isolate Wuhan-Hu-1 (Reference Sequence: NC_045512.2), and SARS-CoV-2 variant Gamma/P.1 (GenBank: OX000832.1). Homologous sequences are highlighted in yellow. Double alanine spacer are shown in red. Furin cleavage sites are italic and underlined. Mutations in SARS-CoV-2 variants compared to PanCoVac are highlighted in green.
