# Supplementary figures and images for: Early protective effect of a (“pan”) coronavirus vaccine (PanCoVac) in Roborovski dwarf hamsters after single-low dose intranasal administration
Source: Front Immunol. 2023 Jul 13;14:1166765. doi: 10.3389/fimmu.2023.1166765 (PMC10372429; doi:10.3389/fimmu.2023.1166765)

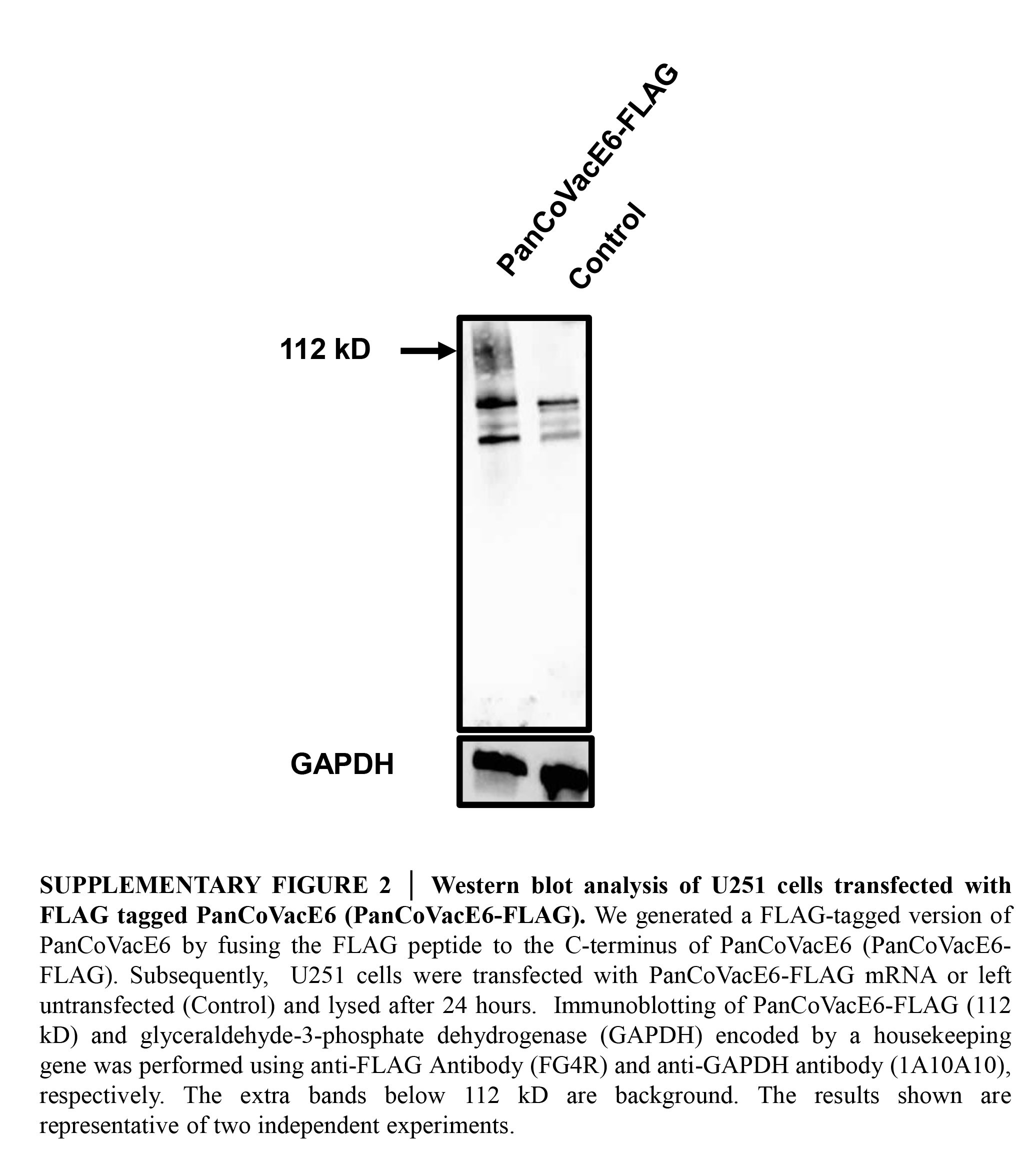

Supplement: Supplementary Figure 2 — (Western blot analysis of FLAG tagged PanCoVac). [file Image_2.tif]
