# Supplementary material for: Early protective effect of a (“pan”) coronavirus vaccine (PanCoVac) in Roborovski dwarf hamsters after single-low dose intranasal administration
Source: Front Immunol. 2023 Jul 13;14:1166765. doi: 10.3389/fimmu.2023.1166765 (PMC10372429; doi:10.3389/fimmu.2023.1166765)
Supplement: Supplementary Figure 3 — (Histopathological scoring of tissue). [file Image_3.pdf]

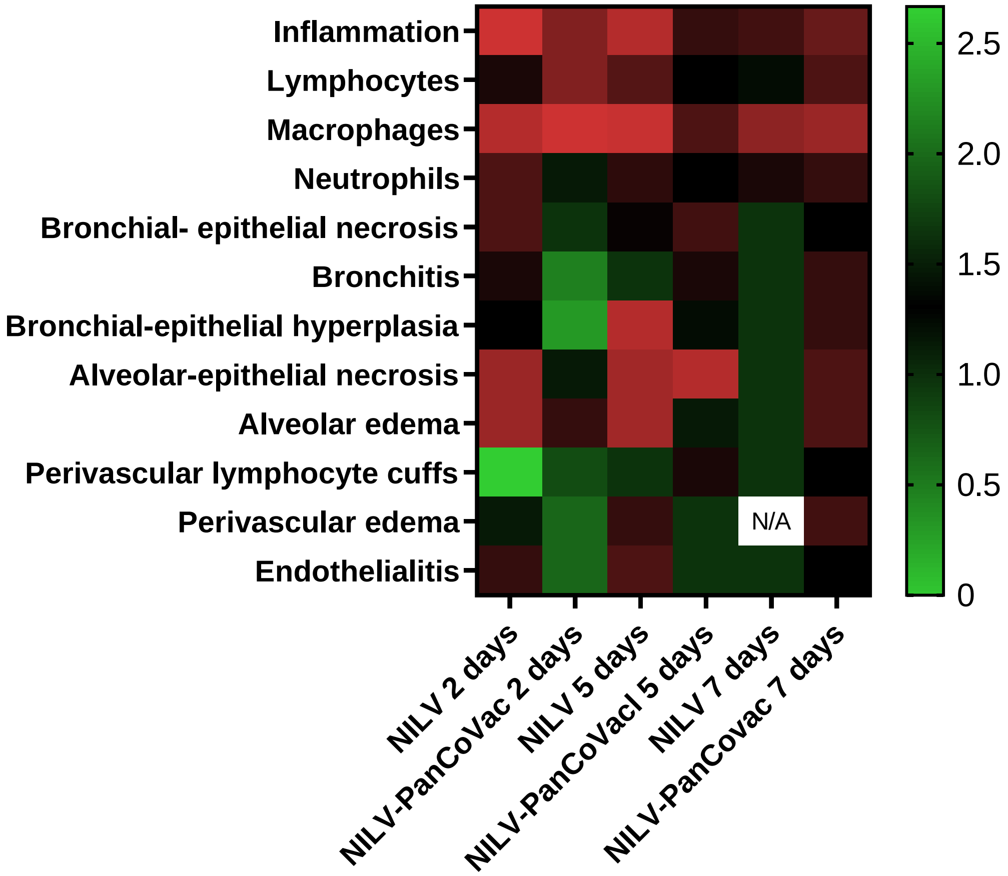

SUPPLEMENTARY FIGURE 3 │ Histopathological scores of lung tissue derived from NILV or NILV-PanCoVac vaccinated Roborovski dwarf hamsters at 2 dpi, 5 dpi and 7 dpi after challenge with SARS-CoV-2. At the indicated time points, three animals of each group were sacrificed and histopathological changes in the lung were scored using a four-scale severity grading system (0: no lesions, 1: mild, 2: moderate, and 3: severe). The mean histopathological scores for lung tissue were calculated at the indicated time points (N/A: not assessed). One animal in the NILV-vaccinated group scheduled for the analysis at 7 dpi (Hamster Nr. 8) died at 4 dpi. The corresponding lung tissue was analyzed together with lung tissue scheduled for 5 dpi.
