# Supplementary material for: Early protective effect of a (“pan”) coronavirus vaccine (PanCoVac) in Roborovski dwarf hamsters after single-low dose intranasal administration
Source: Front Immunol. 2023 Jul 13;14:1166765. doi: 10.3389/fimmu.2023.1166765 (PMC10372429; doi:10.3389/fimmu.2023.1166765)
Supplement: Supplementary Table 1 — (Sex distribution of Roborovski dwarf hamsters). [file Table_1.pdf]

Supplementary Table 1. Number, sex distribution, and analysis date of NILV-PanCoVac-vaccinated hamsters and the control group (vaccination with empty NILV particles), respectively.

Group	Hamster No.	Sex	Analysis date (dpi)
NILV (control)	1	male	5
	2	male	2
	3	male	5
	4	female	5
	5	female	7
	6	female	2
	7	female	7
	8<br>(died at 4 dpi)	female	5
	9	female	2
NILV-PanCoVac	1	male	7
	2	male	2
	3	male	5
	4	female	2
	5	female	5
	6	female	5
	7	female	7
	8	female	7
	9	female	2
