# Supplementary material for: Early protective effect of a (“pan”) coronavirus vaccine (PanCoVac) in Roborovski dwarf hamsters after single-low dose intranasal administration
Source: Front Immunol. 2023 Jul 13;14:1166765. doi: 10.3389/fimmu.2023.1166765 (PMC10372429; doi:10.3389/fimmu.2023.1166765)
Supplement: Supplementary Table 3 — (HLA-II alleles presenting PanCoVac-encoded peptides). [file Table_3.pdf]

Supplementary Table 3. PanCoVac-encoded peptides binding to frequent HLA-DR alleles (HLA-DRB1*01:01, HLA-DRB1*04:01, HLA-DRB1*07:01, HLA-DRB1*09:01, HLA-DRB1*13:02, HLA-DRB1*15:01, HLA-DRB5*01:01) with an IC50 < 1000 nM.

HLA-DRB1*01:01

Allele	#	Start	End	Length	Core Sequence	Peptide Sequence	IC50
HLA-DRB1*01:01	1	849	863	15	YKLGASQRV	TLSYYKLGASQRVAG	2.70
HLA-DRB1*01:01	1	850	864	15	YKLGASQRV	LSYYKLGASQRVAGD	2.80
HLA-DRB1*01:01	1	459	473	15	LQSLQTYVT	TGRLQSLQTYVTQQL	4.40
HLA-DRB1*01:01	1	458	472	15	LQSLQTYVT	ITGRLQSLQTYVTQQ	4.90
HLA-DRB1*01:01	1	460	474	15	LQSLQTYVT	GRLQSLQTYVTQQLI	5.70
HLA-DRB1*01:01	1	420	434	15	VKQLSSNFG	LNTLVKQLSSNFGAI	6.00
HLA-DRB1*01:01	1	421	435	15	VKQLSSNFG	NTLVKQLSSNFGAIS	6.10
HLA-DRB1*01:01	1	457	471	15	LQSLQTYVT	LITGRLQSLQTYVTQ	6.10
HLA-DRB1*01:01	1	708	722	15	LAILTALRL	LVTLAILTALRLCAY	7.10
HLA-DRB1*01:01	1	376	390	15	FAMQMAYRF	QIPFAMQMAYRFNGI	7.30
HLA-DRB1*01:01	1	427	441	15	FGAISSVLN	LSSNFGAISSVLNDI	7.40
HLA-DRB1*01:01	1	709	723	15	LAILTALRL	VTLAILTALRLCAYC	7.60
HLA-DRB1*01:01	1	277	291	15	FNGLTGTGV	VNFNFNGLTGTGVLT	8.10
HLA-DRB1*01:01	1	375	389	15	FAMQMAYRF	LQIPFAMQMAYRFNG	8.10
HLA-DRB1*01:01	1	422	436	15	VKQLSSNFG	TLVKQLSSNFGAISS	8.30
HLA-DRB1*01:01	1	461	475	15	LQSLQTYVT	RLQSLQTYVTQQLIR	8.50
HLA-DRB1*01:01	1	710	724	15	LAILTALRL	TLAILTALRLCAYCC	9.00
HLA-DRB1*01:01	1	454	468	15	LITGRLQSL	IDRLITGRLQSLQTY	9.20
HLA-DRB1*01:01	1	426	440	15	FGAISSVLN	QLSSNFGAISSVLND	9.50
HLA-DRB1*01:01	1	456	470	15	LQSLQTYVT	RLITGRLQSLQTYVT	9.60
HLA-DRB1*01:01	1	377	391	15	FAMQMAYRF	IPFAMQMAYRFNGIG	9.80
HLA-DRB1*01:01	1	453	467	15	IDRLITGRL	QIDRLITGRLQSLQT	10.30
HLA-DRB1*01:01	1	276	290	15	FNGLTGTGV	CVNFNFNGLTGTGVL	11.30
HLA-DRB1*01:01	1	452	466	15	IDRLITGRL	VQIDRLITGRLQSLQ	11.30
HLA-DRB1*01:01	1	706	720	15	LAILTALRL	FLLVTLAILTALRLC	11.60
HLA-DRB1*01:01	1	931	945	15	WFTALTQHG	NTASWFTALTQHGKE	13.90
HLA-DRB1*01:01	1	349	363	15	FNGLTVLPP	CAQKFNGLTVLPPLL	14.00
HLA-DRB1*01:01	1	451	465	15	IDRLITGRL	EVQIDRLITGRLQSL	14.00
HLA-DRB1*01:01	1	430	444	15	FGAISSVLN	NFGAISSVLNDILSR	14.50
HLA-DRB1*01:01	1	425	439	15	FGAISSVLN	KQLSSNFGAISSVLN	15.70
HLA-DRB1*01:01	1	378	392	15	FAMQMAYRF	PFAMQMAYRFNGIGV	15.80

Allele	#	Start	End	Length	Core Sequence	Peptide Sequence	IC50
HLA-DRB1*01:01	1	711	725	15	LAILTALRL	LAILTALRLCAYCCN	16.80
HLA-DRB1*01:01	1	423	437	15	VKQLSSNFG	LVKQLSSNFGAISSV	17.40
HLA-DRB1*01:01	1	455	469	15	LITGRLQSL	DRLITGRLQSLQTYV	19.30
HLA-DRB1*01:01	1	418	432	15	LVKQLSSNF	QALNTLVKQLSSNFG	19.80
HLA-DRB1*01:01	1	348	362	15	FNGLTVLPP	ICAQKFNGLTVLPPL	20.80
HLA-DRB1*01:01	1	839	853	15	ITVATSRTL	PKEITVATSRTLSYY	23.40
HLA-DRB1*01:01	1	930	944	15	WFTALTQHG	NNTASWFTALTQHGK	23.60
HLA-DRB1*01:01	1	705	719	15	LAILTALRL	VFLLVTLAILTALRL	24.70
HLA-DRB1*01:01	1	840	854	15	ITVATSRTL	KEITVATSRTLSYYK	25.70
HLA-DRB1*01:01	1	801	815	15	FNPETNILL	MWSFNPETNILLNVP	28.60
HLA-DRB1*01:01	1	489	503	15	CVLGQSKRV	KMSECVLGQSKRVDF	29.50
HLA-DRB1*01:01	1	490	504	15	CVLGQSKRV	MSECVLGQSKRVDFC	30.40
HLA-DRB1*01:01	1	424	438	15	LSSNFGAIS	VKQLSSNFGAISSVL	32.00
HLA-DRB1*01:01	1	569	583	15	FKNHTSPDV	DKYFKNHTSPDVDLG	32.20
HLA-DRB1*01:01	1	1096	1110	15	FAPSASAFF	PQIAQFAPSASAFFG	34.40
HLA-DRB1*01:01	1	1097	1111	15	FAPSASAFF	QIAQFAPSASAFFGM	35.00
HLA-DRB1*01:01	1	431	445	15	FGAISSVLN	FGAISSVLNDILSRL	35.10
HLA-DRB1*01:01	1	568	582	15	FKNHTSPDV	LDKYFKNHTSPDVDL	36.70
HLA-DRB1*01:01	1	570	584	15	FKNHTSPDV	KYFKNHTSPDVDLGD	37.30
HLA-DRB1*01:01	1	1136	1150	15	YKTFPPTEP	HIDAYKTFPPTEPKK	37.50
HLA-DRB1*01:01	1	838	852	15	ITVATSRTL	LPKEITVATSRTLSY	37.70
HLA-DRB1*01:01	1	1137	1151	15	YKTFPPTEP	IDAYKTFPPTEPKKD	38.20
HLA-DRB1*01:01	1	802	816	15	FNPETNILL	WSFNPETNILLNVPL	39.50
HLA-DRB1*01:01	1	379	393	15	FAMQMAYRF	FAMQMAYRFNGIGVT	40.30
HLA-DRB1*01:01	1	1111	1125	15	IGMEVTPSG	MSRIGMEVTPSGTWL	41.10
HLA-DRB1*01:01	1	449	463	15	IDRLITGRL	EAEVQIDRLITGRLQ	41.70
HLA-DRB1*01:01	1	328	342	15	FNKVTLADA	DLLFNKVTLADAGFA	44.80
HLA-DRB1*01:01	1	1098	1112	15	FAPSASAFF	IAQFAPSASAFFGMS	46.00
HLA-DRB1*01:01	1	488	502	15	CVLGQSKRV	TKMSECVLGQSKRVD	46.60
HLA-DRB1*01:01	1	841	855	15	ITVATSRTL	EITVATSRTLSYYKL	49.50
HLA-DRB1*01:01	1	681	695	15	FVSEETGTL	MYSFVSEETGTLIVN	50.00
HLA-DRB1*01:01	1	1138	1152	15	YKTFPPTEP	DAYKTFPPTEPKKDK	51.00
HLA-DRB1*01:01	1	1095	1109	15	FAPSASAFF	WPQIAQFAPSASAFF	52.30
HLA-DRB1*01:01	1	327	341	15	LLFNKVTLA	EDLLFNKVTLADAGF	52.40
HLA-DRB1*01:01	1	409	423	15	VVNQNAQAL	LQDVVNQNAQALNTL	52.90

Allele	#	Start	End	Length	Core Sequence	Peptide Sequence	IC50
HLA-DRB1*01:01	1	347	361	15	FNGLTVLPP	LICAQKFNGLTVLPP	53.00
HLA-DRB1*01:01	1	765	779	15	WLLWPVTLA	FLWLLWPVTLACFVL	53.20
HLA-DRB1*01:01	1	383	397	15	FNGIGVTQN	MAYRFNGIGVTQNVL	53.40
HLA-DRB1*01:01	1	1175	1189	15	FHNIRGRWT	KQRFHNIRGRWTGDY	55.50
HLA-DRB1*01:01	1	660	674	15	EPVLKGVKL	DDSEPVLKGVKLHYR	58.10
HLA-DRB1*01:01	1	326	340	15	LLFNKVTLA	IEDLLFNKVTLADAG	58.50
HLA-DRB1*01:01	1	296	310	15	FCTQLNRAL	LLQYGSFCTQLNRAL	60.10
HLA-DRB1*01:01	1	1110	1124	15	IGMEVTPSG	GMSRIGMEVTPSGTW	60.90
HLA-DRB1*01:01	1	385	399	15	FNGIGVTQN	YRFNGIGVTQNVLYE	62.00
HLA-DRB1*01:01	1	410	424	15	VVNQNAQAL	QDVVNQNAQALNTLV	62.20
HLA-DRB1*01:01	1	492	506	15	CVLGQSKRV	ECVLGQSKRVDFCGK	62.70
HLA-DRB1*01:01	1	846	860	15	YYKLGASQR	TSRTLSYYKLGASQR	63.10
HLA-DRB1*01:01	1	294	308	15	LLQYGSFCT	NLLLQYGSFCTQLNR	64.60
HLA-DRB1*01:01	1	1112	1126	15	IGMEVTPSG	SRIGMEVTPSGTWLT	65.70
HLA-DRB1*01:01	1	682	696	15	FVSEETGTL	YSFVSEETGTLIVNS	66.40
HLA-DRB1*01:01	1	1135	1149	15	YKTFPPTEP	KHIDAYKTFPPTEPK	66.90
HLA-DRB1*01:01	1	231	245	15	FASVYAWNR	RFASVYAWNRKRISN	69.90
HLA-DRB1*01:01	1	382	396	15	FNGIGVTQN	QMAYRFNGIGVTQNV	70.00
HLA-DRB1*01:01	1	1176	1190	15	FHNIRGRWT	QRFHNIRGRWTGDYK	72.20
HLA-DRB1*01:01	1	1099	1113	15	FAPSASAFF	AQFAPSASAFFGMSR	73.30
HLA-DRB1*01:01	1	1174	1188	15	FHNIRGRWT	KKQRFHNIRGRWTGD	81.60
HLA-DRB1*01:01	1	719	733	15	CNIVNVSLV	LCAYCCNIVNVSLVK	82.20
HLA-DRB1*01:01	1	567	581	15	FKNHTSPDV	ELDKYFKNHTSPDVD	83.10
HLA-DRB1*01:01	1	884	898	15	YEGNSPFHP	STYEGNSPFHPLADN	83.70
HLA-DRB1*01:01	1	1173	1187	15	FHNIRGRWT	DKKQRFHNIRGRWTG	84.70
HLA-DRB1*01:01	1	408	422	15	VVNQNAQAL	KLQDVVNQNAQALNT	86.90
HLA-DRB1*01:01	1	837	851	15	ITVATSRTL	DLPKEITVATSRTLS	87.50
HLA-DRB1*01:01	1	411	425	15	VVNQNAQAL	DVVNQNAQALNTLVK	88.10
HLA-DRB1*01:01	1	798	812	15	FNPETNILL	TRSMWSFNPETNILL	95.20
HLA-DRB1*01:01	1	386	400	15	FNGIGVTQN	RFNGIGVTQNVLYEN	98.40
HLA-DRB1*01:01	1	324	338	15	LLFNKVTLA	SFIEDLLFNKVTLAD	99.10
HLA-DRB1*01:01	1	1113	1127	15	IGMEVTPSG	RIGMEVTPSGTWLTY	101.40
HLA-DRB1*01:01	1	1109	1123	15	IGMEVTPSG	FGMSRIGMEVTPSGT	101.50
HLA-DRB1*01:01	1	1106	1120	15	FFGMSRIGM	SAFFGMSRIGMEVTP	101.90
HLA-DRB1*01:01	1	1094	1108	15	QIAQFAPSA	HWPQIAQFAPSASAF	107.20

Allele	#	Start	End	Length	Core Sequence	Peptide Sequence	IC50
HLA-DRB1*01:01	1	1104	1118	15	FFGMSRIGM	SASAFFGMSRIGMEV	112.00
HLA-DRB1*01:01	1	1177	1191	15	FHNIRGRWT	RFHNIRGRWTGDYKD	112.20
HLA-DRB1*01:01	1	1015	1029	15	LLLDRLNQL	LALLLLDRLNQLESK	117.40
HLA-DRB1*01:01	1	381	395	15	YRFNGIGVT	MQMAYRFNGIGVTQN	122.50
HLA-DRB1*01:01	1	571	585	15	FKNHTSPDV	YFKNHTSPDVDLGDI	123.60
HLA-DRB1*01:01	1	659	673	15	EPVLKGVKL	EDDSEPVLKGVKLHY	129.20
HLA-DRB1*01:01	1	448	462	15	IDRLITGRL	VEAEVQIDRLITGRL	131.00
HLA-DRB1*01:01	1	1100	1114	15	FAPSASAFF	QFAPSASAFFGMSRI	131.90
HLA-DRB1*01:01	1	1108	1122	15	IGMEVTPSG	FFGMSRIGMEVTPSG	133.60
HLA-DRB1*01:01	1	581	595	15	ISGINASVV	DLGDISGINASVVNI	135.60
HLA-DRB1*01:01	1	582	596	15	ISGINASVV	LGDISGINASVVNIQ	137.30
HLA-DRB1*01:01	1	718	732	15	CNIVNVSLV	RLCAYCCNIVNVSLV	138.30
HLA-DRB1*01:01	1	412	426	15	NAQALNTLV	VVNQNAQALNTLVKQ	139.30
HLA-DRB1*01:01	1	683	697	15	FVSEETGTL	SFVSEETGTLIVNSV	140.80
HLA-DRB1*01:01	1	842	856	15	ITVATSRTL	ITVATSRTLSYYKLG	140.90
HLA-DRB1*01:01	1	345	359	15	ICAQKFNGL	RDLICAQKFNGLTVL	146.40
HLA-DRB1*01:01	1	580	594	15	ISGINASVV	VDLGDISGINASVVN	147.60
HLA-DRB1*01:01	1	885	899	15	YEGNSPFHP	TYEGNSPFHPLADNK	156.30
HLA-DRB1*01:01	1	407	421	15	VVNQNAQAL	GKLQDVVNQNAQALN	158.60
HLA-DRB1*01:01	1	414	428	15	NAQALNTLV	NQNAQALNTLVKQLS	158.80
HLA-DRB1*01:01	1	1103	1117	15	FFGMSRIGM	PSASAFFGMSRIGME	163.30
HLA-DRB1*01:01	1	978	992	15	LQLPQGTTL	VLQLPQGTTLPKGFY	163.50
HLA-DRB1*01:01	1	929	943	15	WFTALTQHG	PNNTASWFTALTQHG	170.70
HLA-DRB1*01:01	1	583	597	15	ISGINASVV	GDISGINASVVNIQK	174.20
HLA-DRB1*01:01	1	413	427	15	NAQALNTLV	VNQNAQALNTLVKQL	177.30
HLA-DRB1*01:01	1	232	246	15	YAWNRKRIS	FASVYAWNRKRISNC	183.90
HLA-DRB1*01:01	1	415	429	15	NAQALNTLV	QNAQALNTLVKQLSS	183.90
HLA-DRB1*01:01	1	259	273	15	YQPYRVVVL	GYQPYRVVVLSFELL	184.40
HLA-DRB1*01:01	1	497	511	15	DFCGKGYHL	QSKRVDFCGKGYHLM	191.10
HLA-DRB1*01:01	1	635	649	15	FIAGLIAIV	WLGFIAGLIAIVMVT	194.30
HLA-DRB1*01:01	1	493	507	15	CVLGQSKRV	CVLGQSKRVDFCGKG	197.70
HLA-DRB1*01:01	1	1130	1144	15	ILLNKHIDA	VILLNKHIDAYKTFP	202.40
HLA-DRB1*01:01	1	346	360	15	ICAQKFNGL	DLICAQKFNGLTVLP	207.80
HLA-DRB1*01:01	1	387	401	15	IGVTQNVLY	FNGIGVTQNVLYENQ	219.00
HLA-DRB1*01:01	1	579	593	15	LGDISGINA	DVDLGDISGINASVV	225.20

Allele	#	Start	End	Length	Core Sequence	Peptide Sequence	IC50
HLA-DRB1*01:01	1	584	598	15	ISGINASVV	DISGINASVVNIQKE	227.00
HLA-DRB1*01:01	1	713	727	15	ALRLCAYCC	ILTALRLCAYCCNIV	230.30
HLA-DRB1*01:01	1	416	430	15	LNTLVKQLS	NAQALNTLVKQLSSN	234.10
HLA-DRB1*01:01	1	714	728	15	ALRLCAYCC	LTALRLCAYCCNIVN	241.40
HLA-DRB1*01:01	1	551	565	15	YDPLQPELD	NTVYDPLQPELDSFK	256.00
HLA-DRB1*01:01	1	687	701	15	GTLIVNSVL	EETGTLIVNSVLLFL	270.40
HLA-DRB1*01:01	1	793	807	15	FARTRSMWS	RLFARTRSMWSFNPE	271.60
HLA-DRB1*01:01	1	566	580	15	FKNHTSPDV	EELDKYFKNHTSPDV	292.00
HLA-DRB1*01:01	1	689	703	15	GTLIVNSVL	TGTLIVNSVLLFLAF	297.70
HLA-DRB1*01:01	1	241	255	15	CVADYSVLY	KRISNCVADYSVLYN	300.20
HLA-DRB1*01:01	1	658	672	15	EPVLKGVKL	DEDDSEPVLKGVKLH	301.00
HLA-DRB1*01:01	1	960	974	15	FYYLGTGPE	LSPRWYFYYLGTGPE	303.40
HLA-DRB1*01:01	1	715	729	15	ALRLCAYCC	TALRLCAYCCNIVNV	304.50
HLA-DRB1*01:01	1	235	249	15	NRKRISNCV	VYAWNRKRISNCVAD	306.50
HLA-DRB1*01:01	1	240	254	15	ISNCVADYS	RKRISNCVADYSVLY	314.80
HLA-DRB1*01:01	1	1092	1106	15	QIAQFAPSA	YKHWPQIAQFAPSAS	321.00
HLA-DRB1*01:01	1	700	714	15	FLLVTLAIL	FLAFVVFLLVTLAIL	322.80
HLA-DRB1*01:01	1	552	566	15	YDPLQPELD	TVYDPLQPELDSFKE	323.30
HLA-DRB1*01:01	1	684	698	15	VSEETGTLI	FVSEETGTLIVNSVL	327.00
HLA-DRB1*01:01	1	242	256	15	CVADYSVLY	RISNCVADYSVLYNS	330.50
HLA-DRB1*01:01	1	836	850	15	ITVATSRTL	KDLPKEITVATSRTL	344.90
HLA-DRB1*01:01	1	236	250	15	NRKRISNCV	YAWNRKRISNCVADY	349.10
HLA-DRB1*01:01	1	701	715	15	FLLVTLAIL	LAFVVFLLVTLAILT	356.90
HLA-DRB1*01:01	1	602	616	15	VAKNLNESL	LNEVAKNLNESLIDL	366.20
HLA-DRB1*01:01	1	989	1003	15	FYAEGSRGG	KGFYAEGSRGGSQAS	369.80
HLA-DRB1*01:01	1	1140	1154	15	YKTFPPTEP	YKTFPPTEPKKDKKK	384.60
HLA-DRB1*01:01	1	717	731	15	CAYCCNIVN	LRLCAYCCNIVNVSL	390.70
HLA-DRB1*01:01	1	1134	1148	15	YKTFPPTEP	NKHIDAYKTFPPTEP	392.30
HLA-DRB1*01:01	1	636	650	15	FIAGLIAIV	LGFIAGLIAIVMVTI	396.50
HLA-DRB1*01:01	1	797	811	15	TRSMWSFNP	RTRSMWSFNPETNIL	411.50
HLA-DRB1*01:01	1	603	617	15	VAKNLNESL	NEVAKNLNESLIDLQ	412.30
HLA-DRB1*01:01	1	585	599	15	ISGINASVV	ISGINASVVNIQKEI	413.30
HLA-DRB1*01:01	1	982	996	15	TTLPKGFYA	PQGTTLPKGFYAEGS	416.00
HLA-DRB1*01:01	1	703	717	15	FLLVTLAIL	FVVFLLVTLAILTAL	420.40
HLA-DRB1*01:01	1	794	808	15	TRSMWSFNP	LFARTRSMWSFNPET	421.10

Allele	#	Start	End	Length	Core Sequence	Peptide Sequence	IC50
HLA-DRB1*01:01	1	534	548	15	FVSGNCDVV	DNTFVSGNCDVVIGI	429.80
HLA-DRB1*01:01	1	844	858	15	SRTLSYYKL	VATSRTLSYYKLGAS	429.80
HLA-DRB1*01:01	1	983	997	15	TTLPKGFYA	QGTTLPKGFYAEGSR	431.00
HLA-DRB1*01:01	1	1178	1192	15	FHNIRGRWT	FHNIRGRWTGDYKDD	431.10
HLA-DRB1*01:01	1	690	704	15	LIVNSVLLF	GTLIVNSVLLFLAFV	451.80
HLA-DRB1*01:01	1	704	718	15	FLLVTLAIL	VVFLLVTLAILTALR	452.20
HLA-DRB1*01:01	1	685	699	15	GTLIVNSVL	VSEETGTLIVNSVLL	466.70
HLA-DRB1*01:01	1	1101	1115	15	FAPSASAFF	FAPSASAFFGMSRIG	471.90
HLA-DRB1*01:01	1	388	402	15	IGVTQNVLY	NGIGVTQNVLYENQK	472.40
HLA-DRB1*01:01	1	406	420	15	VVNQNAQAL	LGKLQDVVNQNAQAL	472.80
HLA-DRB1*01:01	1	987	1001	15	FYAEGSRGG	LPKGFYAEGSRGGSQ	474.00
HLA-DRB1*01:01	1	578	592	15	LGDISGINA	PDVDLGDISGINASV	475.40
HLA-DRB1*01:01	1	886	900	15	YEGNSPFHP	YEGNSPFHPLADNKF	481.60
HLA-DRB1*01:01	1	238	252	15	NRKRISNCV	WNRKRISNCVADYSV	484.30
HLA-DRB1*01:01	1	432	446	15	ISSVLNDIL	GAISSVLNDILSRLD	495.40
HLA-DRB1*01:01	1	1131	1145	15	NKHIDAYKT	ILLNKHIDAYKTFPP	510.30
HLA-DRB1*01:01	1	239	253	15	ISNCVADYS	NRKRISNCVADYSVL	510.40
HLA-DRB1*01:01	1	981	995	15	TTLPKGFYA	LPQGTTLPKGFYAEG	513.80
HLA-DRB1*01:01	1	888	902	15	FHPLADNKF	GNSPFHPLADNKFAL	521.70
HLA-DRB1*01:01	1	553	567	15	YDPLQPELD	VYDPLQPELDSFKEE	531.20
HLA-DRB1*01:01	1	795	809	15	TRSMWSFNP	FARTRSMWSFNPETN	543.10
HLA-DRB1*01:01	1	434	448	15	SVLNDILSR	ISSVLNDILSRLDKV	548.20
HLA-DRB1*01:01	1	533	547	15	FVSGNCDVV	TDNTFVSGNCDVVIG	559.50
HLA-DRB1*01:01	1	529	543	15	IITTDNTFV	QIITTDNTFVSGNCD	572.90
HLA-DRB1*01:01	1	637	651	15	FIAGLIAIV	GFIAGLIAIVMVTIA	585.50
HLA-DRB1*01:01	1	212	226	15	ITNLCPFGE	FPNITNLCPFGEVFN	594.60
HLA-DRB1*01:01	1	604	618	15	VAKNLNESL	EVAKNLNESLIDLQE	618.50
HLA-DRB1*01:01	1	601	615	15	VAKNLNESL	RLNEVAKNLNESLID	623.30
HLA-DRB1*01:01	1	980	994	15	TTLPKGFYA	QLPQGTTLPKGFYAE	640.90
HLA-DRB1*01:01	1	211	225	15	ITNLCPFGE	RFPNITNLCPFGEVF	718.10
HLA-DRB1*01:01	1	1064	1078	15	VTQAFGRRG	YNVTQAFGRRGPEQT	737.90
HLA-DRB1*01:01	1	657	671	15	EPVLKGVKL	FDEDDSEPVLKGVKL	751.70
HLA-DRB1*01:01	1	990	1004	15	FYAEGSRGG	GFYAEGSRGGSQASS	757.20
HLA-DRB1*01:01	1	214	228	15	ITNLCPFGE	NITNLCPFGEVFNAT	791.70
HLA-DRB1*01:01	1	928	942	15	NTASWFTAL	LPNNTASWFTALTQH	811.30

Allele	#	Start	End	Length	Core Sequence	Peptide Sequence	IC50
HLA-DRB1*01:01	1	532	546	15	FVSGNCDVV	TTDNTFVSGNCDVVI	814.30
HLA-DRB1*01:01	1	834	848	15	IKDLPKEIT	DIKDLPKEITVATSR	831.00
HLA-DRB1*01:01	1	600	614	15	VAKNLNESL	DRLNEVAKNLNESLI	835.30
HLA-DRB1*01:01	1	1090	1104	15	HWPQIAQFA	TDYKHWPQIAQFAPS	848.70
HLA-DRB1*01:01	1	835	849	15	LPKEITVAT	IKDLPKEITVATSRT	894.00
HLA-DRB1*01:01	1	572	586	15	FKNHTSPDV	FKNHTSPDVDLGDIS	930.60
HLA-DRB1*01:01	1	832	846	15	IKDLPKEIT	RCDIKDLPKEITVAT	941.30
HLA-DRB1*01:01	1	437	451	15	DILSRLDKV	VLNDILSRLDKVEAE	949.10
HLA-DRB1*01:01	1	1089	1103	15	HWPQIAQFA	GTDYKHWPQIAQFAP	960.90
HLA-DRB1*01:01	1	833	847	15	IKDLPKEIT	CDIKDLPKEITVATS	966.00
HLA-DRB1*01:01	1	1132	1146	15	NKHIDAYKT	LLNKHIDAYKTFPPT	984.40
HLA-DRB1*01:01	1	1088	1102	15	DYKHWPQIA	QGTDYKHWPQIAQFA	989.20

HLA-DRB1*04:01

Allele	#	Start	End	Length	Core Sequence	Peptide Sequence	IC50
HLA-DRB1*04:01	1	792	806	15	FARTRSMWS	FRLFARTRSMWSFNP	10.70
HLA-DRB1*04:01	1	791	805	15	FARTRSMWS	SFRLFARTRSMWSFN	10.80
HLA-DRB1*04:01	1	793	807	15	FARTRSMWS	RLFARTRSMWSFNPE	11.50
HLA-DRB1*04:01	1	421	435	15	VKQLSSNFG	NTLVKQLSSNFGAIS	16.00
HLA-DRB1*04:01	1	420	434	15	VKQLSSNFG	LNTLVKQLSSNFGAI	16.20
HLA-DRB1*04:01	1	794	808	15	FARTRSMWS	LFARTRSMWSFNPET	18.00
HLA-DRB1*04:01	1	422	436	15	VKQLSSNFG	TLVKQLSSNFGAISS	22.00
HLA-DRB1*04:01	1	849	863	15	YKLGASQRV	TLSYYKLGASQRVAG	25.50
HLA-DRB1*04:01	1	850	864	15	YKLGASQRV	LSYYKLGASQRVAGD	28.40
HLA-DRB1*04:01	1	423	437	15	VKQLSSNFG	LVKQLSSNFGAISSV	42.50
HLA-DRB1*04:01	1	296	310	15	YGSFCTQLN	LLQYGSFCTQLNRAL	49.30
HLA-DRB1*04:01	1	800	814	15	FNPETNILL	SMWSFNPETNILLNV	57.80
HLA-DRB1*04:01	1	801	815	15	FNPETNILL	MWSFNPETNILLNVP	58.60
HLA-DRB1*04:01	1	418	432	15	VKQLSSNFG	QALNTLVKQLSSNFG	61.50
HLA-DRB1*04:01	1	802	816	15	FNPETNILL	WSFNPETNILLNVPL	68.00
HLA-DRB1*04:01	1	847	861	15	YKLGASQRV	SRTLSYYKLGASQRV	68.70
HLA-DRB1*04:01	1	1097	1111	15	FAPSASAFF	QIAQFAPSASAFFGM	69.50
HLA-DRB1*04:01	1	1096	1110	15	FAPSASAFF	PQIAQFAPSASAFFG	71.30
HLA-DRB1*04:01	1	1098	1112	15	FAPSASAFF	IAQFAPSASAFFGMS	77.90
HLA-DRB1*04:01	1	383	397	15	FNGIGVTQN	MAYRFNGIGVTQNVL	79.60
HLA-DRB1*04:01	1	840	854	15	VATSRTLSY	KEITVATSRTLSYYK	82.60

Allele	#	Start	End	Length	Core Sequence	Peptide Sequence	IC50
HLA-DRB1*04:01	1	681	695	15	FVSEETGTL	MYSFVSEETGTLIVN	88.30
HLA-DRB1*04:01	1	382	396	15	FNGIGVTQN	QMAYRFNGIGVTQNV	89.00
HLA-DRB1*04:01	1	841	855	15	VATSRTLSY	EITVATSRTLSYYKL	90.10
HLA-DRB1*04:01	1	428	442	15	FGAISSVLN	SSNFGAISSVLNDIL	99.90
HLA-DRB1*04:01	1	1015	1029	15	LLLDRLNQL	LALLLLDRLNQLESK	107.00
HLA-DRB1*04:01	1	327	341	15	LLFNKVTLA	EDLLFNKVTLADAGF	107.50
HLA-DRB1*04:01	1	427	441	15	FGAISSVLN	LSSNFGAISSVLNDI	108.70
HLA-DRB1*04:01	1	795	809	15	FARTRSMWS	FARTRSMWSFNPETN	111.70
HLA-DRB1*04:01	1	842	856	15	VATSRTLSY	ITVATSRTLSYYKLG	112.00
HLA-DRB1*04:01	1	385	399	15	FNGIGVTQN	YRFNGIGVTQNVLYE	113.30
HLA-DRB1*04:01	1	326	340	15	LLFNKVTLA	IEDLLFNKVTLADAG	119.70
HLA-DRB1*04:01	1	294	308	15	YGSFCTQLN	NLLLQYGSFCTQLNR	120.20
HLA-DRB1*04:01	1	426	440	15	FGAISSVLN	QLSSNFGAISSVLND	120.50
HLA-DRB1*04:01	1	839	853	15	VATSRTLSY	PKEITVATSRTLSYY	122.00
HLA-DRB1*04:01	1	529	543	15	ITTDNTFVS	QIITTDNTFVSGNCD	123.10
HLA-DRB1*04:01	1	682	696	15	FVSEETGTL	YSFVSEETGTLIVNS	129.40
HLA-DRB1*04:01	1	1095	1109	15	FAPSASAFF	WPQIAQFAPSASAFF	130.70
HLA-DRB1*04:01	1	798	812	15	FNPETNILL	TRSMWSFNPETNILL	133.90
HLA-DRB1*04:01	1	931	945	15	WFTALTQHG	NTASWFTALTQHGKE	135.00
HLA-DRB1*04:01	1	430	444	15	FGAISSVLN	NFGAISSVLNDILSR	158.80
HLA-DRB1*04:01	1	425	439	15	FGAISSVLN	KQLSSNFGAISSVLN	161.70
HLA-DRB1*04:01	1	381	395	15	FNGIGVTQN	MQMAYRFNGIGVTQN	166.40
HLA-DRB1*04:01	1	324	338	15	LLFNKVTLA	SFIEDLLFNKVTLAD	187.50
HLA-DRB1*04:01	1	568	582	15	FKNHTSPDV	LDKYFKNHTSPDVDL	188.50
HLA-DRB1*04:01	1	930	944	15	WFTALTQHG	NNTASWFTALTQHGK	192.80
HLA-DRB1*04:01	1	1100	1114	15	FAPSASAFF	QFAPSASAFFGMSRI	195.20
HLA-DRB1*04:01	1	718	732	15	YCCNIVNVS	RLCAYCCNIVNVSLV	203.10
HLA-DRB1*04:01	1	459	473	15	LQSLQTYVT	TGRLQSLQTYVTQQL	207.80
HLA-DRB1*04:01	1	386	400	15	FNGIGVTQN	RFNGIGVTQNVLYEN	208.40
HLA-DRB1*04:01	1	424	438	15	LSSNFGAIS	VKQLSSNFGAISSVL	215.40
HLA-DRB1*04:01	1	717	731	15	CAYCCNIVN	LRLCAYCCNIVNVSL	215.50
HLA-DRB1*04:01	1	1112	1126	15	IGMEVTPSG	SRIGMEVTPSGTWLT	216.60
HLA-DRB1*04:01	1	569	583	15	FKNHTSPDV	DKYFKNHTSPDVDLG	216.80
HLA-DRB1*04:01	1	242	256	15	CVADYSVLY	RISNCVADYSVLYNS	219.20
HLA-DRB1*04:01	1	348	362	15	FNGLTVLPP	ICAQKFNGLTVLPPL	225.60
HLA-DRB1*04:01	1	1110	1124	15	IGMEVTPSG	GMSRIGMEVTPSGTW	232.10
HLA-DRB1*04:01	1	683	697	15	FVSEETGTL	SFVSEETGTLIVNSV	232.40

Allele	#	Start	End	Length	Core Sequence	Peptide Sequence	IC50
HLA-DRB1*04:01	1	241	255	15	CVADYSVLY	KRISNCVADYSVLYN	237.90
HLA-DRB1*04:01	1	567	581	15	FKNHTSPDV	ELDKYFKNHTSPDVD	241.20
HLA-DRB1*04:01	1	1111	1125	15	IGMEVTPSG	MSRIGMEVTPSGTWL	243.90
HLA-DRB1*04:01	1	989	1003	15	FYAEGSRGG	KGFYAEGSRGGSQAS	250.60
HLA-DRB1*04:01	1	460	474	15	LQSLQTYVT	GRLQSLQTYVTQQLI	255.60
HLA-DRB1*04:01	1	719	733	15	YCCNIVNVS	LCAYCCNIVNVSLVK	258.00
HLA-DRB1*04:01	1	210	224	15	FPNITNLCP	VRFPNITNLCPFGEV	261.90
HLA-DRB1*04:01	1	1113	1127	15	IGMEVTPSG	RIGMEVTPSGTWLTY	264.50
HLA-DRB1*04:01	1	458	472	15	LQSLQTYVT	ITGRLQSLQTYVTQQ	265.90
HLA-DRB1*04:01	1	987	1001	15	FYAEGSRGG	LPKGFYAEGSRGGSQ	269.30
HLA-DRB1*04:01	1	838	852	15	VATSRTLSY	LPKEITVATSRTLSY	275.30
HLA-DRB1*04:01	1	570	584	15	FKNHTSPDV	KYFKNHTSPDVDLGD	280.10
HLA-DRB1*04:01	1	406	420	15	LQDVVNQNA	LGKLQDVVNQNAQAL	284.90
HLA-DRB1*04:01	1	1109	1123	15	IGMEVTPSG	FGMSRIGMEVTPSGT	289.40
HLA-DRB1*04:01	1	1108	1122	15	IGMEVTPSG	FFGMSRIGMEVTPSG	319.90
HLA-DRB1*04:01	1	431	445	15	ISSVLNDIL	FGAISSVLNDILSRL	342.40
HLA-DRB1*04:01	1	407	421	15	LQDVVNQNA	GKLQDVVNQNAQALN	350.90
HLA-DRB1*04:01	1	240	254	15	CVADYSVLY	RKRISNCVADYSVLY	359.80
HLA-DRB1*04:01	1	276	290	15	FNGLTGTGV	CVNFNFNGLTGTGVL	370.80
HLA-DRB1*04:01	1	347	361	15	FNGLTVLPP	LICAQKFNGLTVLPP	371.80
HLA-DRB1*04:01	1	530	544	15	ITTDNTFVS	IITTDNTFVSGNCDV	376.60
HLA-DRB1*04:01	1	986	1000	15	FYAEGSRGG	TLPKGFYAEGSRGGS	377.10
HLA-DRB1*04:01	1	211	225	15	FPNITNLCP	RFPNITNLCPFGEVF	396.40
HLA-DRB1*04:01	1	566	580	15	FKNHTSPDV	EELDKYFKNHTSPDV	401.60
HLA-DRB1*04:01	1	457	471	15	LQSLQTYVT	LITGRLQSLQTYVTQ	409.00
HLA-DRB1*04:01	1	1094	1108	15	QIAQFAPSA	HWPQIAQFAPSASAF	415.50
HLA-DRB1*04:01	1	990	1004	15	FYAEGSRGG	GFYAEGSRGGSQASS	417.40
HLA-DRB1*04:01	1	461	475	15	LQSLQTYVT	RLQSLQTYVTQQLIR	431.10
HLA-DRB1*04:01	1	277	291	15	FNGLTGTGV	VNFNFNGLTGTGVLT	458.70
HLA-DRB1*04:01	1	846	860	15	LSYYKLGAS	TSRTLSYYKLGASQR	479.10
HLA-DRB1*04:01	1	375	389	15	FAMQMAYRF	LQIPFAMQMAYRFNG	511.20
HLA-DRB1*04:01	1	454	468	15	LITGRLQSL	IDRLITGRLQSLQTY	556.50
HLA-DRB1*04:01	1	456	470	15	LQSLQTYVT	RLITGRLQSLQTYVT	556.50
HLA-DRB1*04:01	1	1092	1106	15	QIAQFAPSA	YKHWPQIAQFAPSAS	571.70
HLA-DRB1*04:01	1	715	729	15	CAYCCNIVN	TALRLCAYCCNIVNV	618.00
HLA-DRB1*04:01	1	259	273	15	YQPYRVVVL	GYQPYRVVVLSFELL	628.70
HLA-DRB1*04:01	1	376	390	15	FAMQMAYRF	QIPFAMQMAYRFNGI	640.00

Allele	#	Start	End	Length	Core Sequence	Peptide Sequence	IC50
HLA-DRB1*04:01	1	571	585	15	FKNHTSPDV	YFKNHTSPDVDLGDI	667.80
HLA-DRB1*04:01	1	797	811	15	SMWSFNPET	RTRSMWSFNPETNIL	671.60
HLA-DRB1*04:01	1	408	422	15	LQDVVNQNA	KLQDVVNQNAQALNT	679.50
HLA-DRB1*04:01	1	585	599	15	INASVVNIQ	ISGINASVVNIQKEI	680.80
HLA-DRB1*04:01	1	455	469	15	LITGRLQSL	DRLITGRLQSLQTYV	684.30
HLA-DRB1*04:01	1	929	943	15	WFTALTQHG	PNNTASWFTALTQHG	684.90
HLA-DRB1*04:01	1	583	597	15	INASVVNIQ	GDISGINASVVNIQK	695.10
HLA-DRB1*04:01	1	584	598	15	INASVVNIQ	DISGINASVVNIQKE	705.00
HLA-DRB1*04:01	1	453	467	15	LITGRLQSL	QIDRLITGRLQSLQT	711.30
HLA-DRB1*04:01	1	635	649	15	FIAGLIAIV	WLGFIAGLIAIVMVT	721.30
HLA-DRB1*04:01	1	636	650	15	FIAGLIAIV	LGFIAGLIAIVMVTI	727.40
HLA-DRB1*04:01	1	377	391	15	FAMQMAYRF	IPFAMQMAYRFNGIG	740.40
HLA-DRB1*04:01	1	1089	1103	15	YKHWPQIAQ	GTDYKHWPQIAQFAP	801.00
HLA-DRB1*04:01	1	452	466	15	LITGRLQSL	VQIDRLITGRLQSLQ	864.20
HLA-DRB1*04:01	1	451	465	15	VQIDRLITG	EVQIDRLITGRLQSL	866.80
HLA-DRB1*04:01	1	378	392	15	FAMQMAYRF	PFAMQMAYRFNGIGV	882.50
HLA-DRB1*04:01	1	387	401	15	IGVTQNVLY	FNGIGVTQNVLYENQ	901.20
HLA-DRB1*04:01	1	1090	1104	15	YKHWPQIAQ	TDYKHWPQIAQFAPS	916.50
HLA-DRB1*04:01	1	844	858	15	VATSRTLSY	VATSRTLSYYKLGAS	957.30
HLA-DRB1*04:01	1	1136	1150	15	YKTFPPTEP	HIDAYKTFPPTEPKK	971.30
HLA-DRB1*04:01	1	1135	1149	15	YKTFPPTEP	KHIDAYKTFPPTEPK	986.70
HLA-DRB1*04:01	1	553	567	15	LQPELDSFK	VYDPLQPELDSFKEE	987.50

HLA-DRB1*07:01

Allele	#	Start	End	Length	Core Sequence	Peptide Sequence	IC50
HLA-DRB1*07:01	1	259	273	15	RVVVLSFEL	GYQPYRVVVLSFELL	23.60
HLA-DRB1*07:01	1	793	807	15	FARTRSMWS	RLFARTRSMWSFNPE	34.30
HLA-DRB1*07:01	1	836	850	15	ITVATSRTL	KDLPKEITVATSRTL	34.30
HLA-DRB1*07:01	1	840	854	15	ITVATSRTL	KEITVATSRTLSYYK	40.30
HLA-DRB1*07:01	1	841	855	15	ITVATSRTL	EITVATSRTLSYYKL	41.30
HLA-DRB1*07:01	1	1095	1109	15	FAPSASAFF	WPQIAQFAPSASAFF	41.50
HLA-DRB1*07:01	1	1096	1110	15	FAPSASAFF	PQIAQFAPSASAFFG	53.70
HLA-DRB1*07:01	1	378	392	15	FAMQMAYRF	PFAMQMAYRFNGIGV	56.10
HLA-DRB1*07:01	1	376	390	15	FAMQMAYRF	QIPFAMQMAYRFNGI	56.30
HLA-DRB1*07:01	1	837	851	15	ITVATSRTL	DLPKEITVATSRTLS	56.70
HLA-DRB1*07:01	1	838	852	15	ITVATSRTL	LPKEITVATSRTLSY	56.90

Allele	#	Start	End	Length	Core Sequence	Peptide Sequence	IC50
HLA-DRB1*07:01	1	794	808	15	FARTRSMWS	LFARTRSMWSFNPET	57.60
HLA-DRB1*07:01	1	1098	1112	15	FAPSASAFF	IAQFAPSASAFFGMS	59.40
HLA-DRB1*07:01	1	375	389	15	FAMQMAYRF	LQIPFAMQMAYRFNG	62.90
HLA-DRB1*07:01	1	1100	1114	15	FAPSASAFF	QFAPSASAFFGMSRI	64.20
HLA-DRB1*07:01	1	839	853	15	ITVATSRTL	PKEITVATSRTLSYY	67.10
HLA-DRB1*07:01	1	842	856	15	TSRTLSYYK	ITVATSRTLSYYKLG	67.30
HLA-DRB1*07:01	1	377	391	15	FAMQMAYRF	IPFAMQMAYRFNGIG	70.70
HLA-DRB1*07:01	1	705	719	15	LAILTALRL	VFLLVTLAILTALRL	75.90
HLA-DRB1*07:01	1	379	393	15	FAMQMAYRF	FAMQMAYRFNGIGVT	76.80
HLA-DRB1*07:01	1	276	290	15	FNGLTGTGV	CVNFNFNGLTGTGVL	79.20
HLA-DRB1*07:01	1	849	863	15	YKLGASQRV	TLSYYKLGASQRVAG	84.70
HLA-DRB1*07:01	1	681	695	15	SEETGTLIV	MYSFVSEETGTLIVN	104.50
HLA-DRB1*07:01	1	277	291	15	FNGLTGTGV	VNFNFNGLTGTGVLT	105.10
HLA-DRB1*07:01	1	706	720	15	LAILTALRL	FLLVTLAILTALRLC	105.80
HLA-DRB1*07:01	1	850	864	15	YKLGASQRV	LSYYKLGASQRVAGD	107.90
HLA-DRB1*07:01	1	710	724	15	LAILTALRL	TLAILTALRLCAYCC	123.00
HLA-DRB1*07:01	1	1101	1115	15	FAPSASAFF	FAPSASAFFGMSRIG	132.60
HLA-DRB1*07:01	1	795	809	15	FARTRSMWS	FARTRSMWSFNPETN	133.60
HLA-DRB1*07:01	1	682	696	15	SEETGTLIV	YSFVSEETGTLIVNS	151.60
HLA-DRB1*07:01	1	711	725	15	LAILTALRL	LAILTALRLCAYCCN	153.50
HLA-DRB1*07:01	1	1113	1127	15	MEVTPSGTW	RIGMEVTPSGTWLTY	160.40
HLA-DRB1*07:01	1	1173	1187	15	FHNIRGRWT	DKKQRFHNIRGRWTG	161.00
HLA-DRB1*07:01	1	427	441	15	NFGAISSVL	LSSNFGAISSVLNDI	163.40
HLA-DRB1*07:01	1	1174	1188	15	FHNIRGRWT	KKQRFHNIRGRWTGD	170.50
HLA-DRB1*07:01	1	1104	1118	15	GMSRIGMEV	SASAFFGMSRIGMEV	170.60
HLA-DRB1*07:01	1	385	399	15	IGVTQNVLY	YRFNGIGVTQNVLYE	176.10
HLA-DRB1*07:01	1	635	649	15	WLGFIAGLI	WLGFIAGLIAIVMVT	176.60
HLA-DRB1*07:01	1	460	474	15	LQTYVTQQL	GRLQSLQTYVTQQLI	179.00
HLA-DRB1*07:01	1	708	722	15	LAILTALRL	LVTLAILTALRLCAY	184.60
HLA-DRB1*07:01	1	459	473	15	LQTYVTQQL	TGRLQSLQTYVTQQL	189.40
HLA-DRB1*07:01	1	1175	1189	15	FHNIRGRWT	KQRFHNIRGRWTGDY	191.60
HLA-DRB1*07:01	1	425	439	15	NFGAISSVL	KQLSSNFGAISSVLN	192.50
HLA-DRB1*07:01	1	718	732	15	CNIVNVSLV	RLCAYCCNIVNVSLV	204.50
HLA-DRB1*07:01	1	709	723	15	LAILTALRL	VTLAILTALRLCAYC	205.60
HLA-DRB1*07:01	1	798	812	15	FNPETNILL	TRSMWSFNPETNILL	205.70
HLA-DRB1*07:01	1	383	397	15	GIGVTQNVL	MAYRFNGIGVTQNVL	206.60
HLA-DRB1*07:01	1	888	902	15	FHPLADNKF	GNSPFHPLADNKFAL	206.70

Allele	#	Start	End	Length	Core Sequence	Peptide Sequence	IC50
HLA-DRB1*07:01	1	719	733	15	CNIVNVSLV	LCAYCCNIVNVSLVK	207.70
HLA-DRB1*07:01	1	1111	1125	15	MEVTPSGTW	MSRIGMEVTPSGTWL	210.80
HLA-DRB1*07:01	1	231	245	15	FASVYAWNR	RFASVYAWNRKRISN	211.30
HLA-DRB1*07:01	1	685	699	15	SEETGTLIV	VSEETGTLIVNSVLL	223.70
HLA-DRB1*07:01	1	684	698	15	SEETGTLIV	FVSEETGTLIVNSVL	229.20
HLA-DRB1*07:01	1	424	438	15	NFGAISSVL	VKQLSSNFGAISSVL	230.60
HLA-DRB1*07:01	1	426	440	15	NFGAISSVL	QLSSNFGAISSVLND	231.40
HLA-DRB1*07:01	1	296	310	15	FCTQLNRAL	LLQYGSFCTQLNRAL	232.10
HLA-DRB1*07:01	1	386	400	15	IGVTQNVLY	RFNGIGVTQNVLYEN	247.90
HLA-DRB1*07:01	1	1106	1120	15	GMSRIGMEV	SAFFGMSRIGMEVTP	255.80
HLA-DRB1*07:01	1	1064	1078	15	YNVTQAFGR	YNVTQAFGRRGPEQT	259.10
HLA-DRB1*07:01	1	1176	1190	15	FHNIRGRWT	QRFHNIRGRWTGDYK	272.90
HLA-DRB1*07:01	1	1177	1191	15	FHNIRGRWT	RFHNIRGRWTGDYKD	277.40
HLA-DRB1*07:01	1	461	475	15	LQTYVTQQL	RLQSLQTYVTQQLIR	281.10
HLA-DRB1*07:01	1	886	900	15	FHPLADNKF	YEGNSPFHPLADNKF	281.20
HLA-DRB1*07:01	1	1103	1117	15	SAFFGMSRI	PSASAFFGMSRIGME	307.20
HLA-DRB1*07:01	1	568	582	15	FKNHTSPDV	LDKYFKNHTSPDVDL	315.80
HLA-DRB1*07:01	1	382	396	15	AYRFNGIGV	QMAYRFNGIGVTQNV	317.60
HLA-DRB1*07:01	1	381	395	15	AYRFNGIGV	MQMAYRFNGIGVTQN	326.60
HLA-DRB1*07:01	1	456	470	15	RLQSLQTYV	RLITGRLQSLQTYVT	343.80
HLA-DRB1*07:01	1	457	471	15	RLQSLQTYV	LITGRLQSLQTYVTQ	344.20
HLA-DRB1*07:01	1	1110	1124	15	MEVTPSGTW	GMSRIGMEVTPSGTW	347.60
HLA-DRB1*07:01	1	715	729	15	ALRLCAYCC	TALRLCAYCCNIVNV	354.00
HLA-DRB1*07:01	1	765	779	15	WPVTLACFV	FLWLLWPVTLACFVL	362.90
HLA-DRB1*07:01	1	714	728	15	ALRLCAYCC	LTALRLCAYCCNIVN	367.10
HLA-DRB1*07:01	1	713	727	15	ALRLCAYCC	ILTALRLCAYCCNIV	374.90
HLA-DRB1*07:01	1	387	401	15	IGVTQNVLY	FNGIGVTQNVLYENQ	384.50
HLA-DRB1*07:01	1	458	472	15	RLQSLQTYV	ITGRLQSLQTYVTQQ	408.00
HLA-DRB1*07:01	1	489	503	15	CVLGQSKRV	KMSECVLGQSKRVDF	414.60
HLA-DRB1*07:01	1	802	816	15	FNPETNILL	WSFNPETNILLNVPL	424.60
HLA-DRB1*07:01	1	349	363	15	FNGLTVLPP	CAQKFNGLTVLPPLL	428.80
HLA-DRB1*07:01	1	801	815	15	FNPETNILL	MWSFNPETNILLNVP	440.20
HLA-DRB1*07:01	1	569	583	15	FKNHTSPDV	DKYFKNHTSPDVDLG	460.30
HLA-DRB1*07:01	1	232	246	15	FASVYAWNR	FASVYAWNRKRISNC	474.70
HLA-DRB1*07:01	1	567	581	15	FKNHTSPDV	ELDKYFKNHTSPDVD	480.00
HLA-DRB1*07:01	1	532	546	15	TFVSGNCDV	TTDNTFVSGNCDVVI	487.20
HLA-DRB1*07:01	1	570	584	15	FKNHTSPDV	KYFKNHTSPDVDLGD	490.20

Allele	#	Start	End	Length	Core Sequence	Peptide Sequence	IC50
HLA-DRB1*07:01	1	327	341	15	FNKVTLADA	EDLLFNKVTLADAGF	506.30
HLA-DRB1*07:01	1	420	434	15	LVKQLSSNF	LNTLVKQLSSNFGAI	506.50
HLA-DRB1*07:01	1	566	580	15	FKNHTSPDV	EELDKYFKNHTSPDV	508.50
HLA-DRB1*07:01	1	490	504	15	CVLGQSKRV	MSECVLGQSKRVDFC	510.60
HLA-DRB1*07:01	1	413	427	15	NAQALNTLV	VNQNAQALNTLVKQL	526.60
HLA-DRB1*07:01	1	421	435	15	LVKQLSSNF	NTLVKQLSSNFGAIS	537.50
HLA-DRB1*07:01	1	448	462	15	IDRLITGRL	VEAEVQIDRLITGRL	539.20
HLA-DRB1*07:01	1	455	469	15	RLQSLQTYV	DRLITGRLQSLQTYV	542.80
HLA-DRB1*07:01	1	388	402	15	IGVTQNVLY	NGIGVTQNVLYENQK	566.90
HLA-DRB1*07:01	1	451	465	15	IDRLITGRL	EVQIDRLITGRLQSL	573.40
HLA-DRB1*07:01	1	348	362	15	FNGLTVLPP	ICAQKFNGLTVLPPL	574.30
HLA-DRB1*07:01	1	326	340	15	FNKVTLADA	IEDLLFNKVTLADAG	576.80
HLA-DRB1*07:01	1	700	714	15	FLLVTLAIL	FLAFVVFLLVTLAIL	582.70
HLA-DRB1*07:01	1	431	445	15	ISSVLNDIL	FGAISSVLNDILSRL	591.50
HLA-DRB1*07:01	1	1178	1192	15	FHNIRGRWT	FHNIRGRWTGDYKDD	593.50
HLA-DRB1*07:01	1	422	436	15	LVKQLSSNF	TLVKQLSSNFGAISS	612.10
HLA-DRB1*07:01	1	410	424	15	NAQALNTLV	QDVVNQNAQALNTLV	620.30
HLA-DRB1*07:01	1	701	715	15	FLLVTLAIL	LAFVVFLLVTLAILT	620.80
HLA-DRB1*07:01	1	687	701	15	TLIVNSVLL	EETGTLIVNSVLLFL	626.40
HLA-DRB1*07:01	1	449	463	15	IDRLITGRL	EAEVQIDRLITGRLQ	656.00
HLA-DRB1*07:01	1	533	547	15	TFVSGNCDV	TDNTFVSGNCDVVIG	661.50
HLA-DRB1*07:01	1	345	359	15	AQKFNGLTV	RDLICAQKFNGLTVL	665.30
HLA-DRB1*07:01	1	717	731	15	LCAYCCNIV	LRLCAYCCNIVNVSL	668.30
HLA-DRB1*07:01	1	235	249	15	NRKRISNCV	VYAWNRKRISNCVAD	672.70
HLA-DRB1*07:01	1	452	466	15	IDRLITGRL	VQIDRLITGRLQSLQ	676.90
HLA-DRB1*07:01	1	414	428	15	NAQALNTLV	NQNAQALNTLVKQLS	679.40
HLA-DRB1*07:01	1	703	717	15	FLLVTLAIL	FVVFLLVTLAILTAL	679.50
HLA-DRB1*07:01	1	411	425	15	NAQALNTLV	DVVNQNAQALNTLVK	685.50
HLA-DRB1*07:01	1	534	548	15	TFVSGNCDV	DNTFVSGNCDVVIGI	685.50
HLA-DRB1*07:01	1	618	632	15	YEQYIKWPW	ELGKYEQYIKWPWYI	709.00
HLA-DRB1*07:01	1	423	437	15	KQLSSNFGA	LVKQLSSNFGAISSV	736.40
HLA-DRB1*07:01	1	1108	1122	15	GMSRIGMEV	FFGMSRIGMEVTPSG	765.90
HLA-DRB1*07:01	1	1131	1145	15	IDAYKTFPP	ILLNKHIDAYKTFPP	767.70
HLA-DRB1*07:01	1	453	467	15	IDRLITGRL	QIDRLITGRLQSLQT	782.00
HLA-DRB1*07:01	1	347	361	15	FNGLTVLPP	LICAQKFNGLTVLPP	803.20
HLA-DRB1*07:01	1	492	506	15	CVLGQSKRV	ECVLGQSKRVDFCGK	806.90
HLA-DRB1*07:01	1	530	544	15	TFVSGNCDV	IITTDNTFVSGNCDV	816.90

Allele	#	Start	End	Length	Core Sequence	Peptide Sequence	IC50
HLA-DRB1*07:01	1	797	811	15	WSFNPETNI	RTRSMWSFNPETNIL	822.60
HLA-DRB1*07:01	1	324	338	15	LLFNKVTLA	SFIEDLLFNKVTLAD	831.30
HLA-DRB1*07:01	1	636	650	15	LGFIAGLIA	LGFIAGLIAIVMVTI	837.40
HLA-DRB1*07:01	1	582	596	15	ISGINASVV	LGDISGINASVVNIQ	841.50
HLA-DRB1*07:01	1	412	426	15	NAQALNTLV	VVNQNAQALNTLVKQ	853.30
HLA-DRB1*07:01	1	581	595	15	ISGINASVV	DLGDISGINASVVNI	866.60
HLA-DRB1*07:01	1	488	502	15	CVLGQSKRV	TKMSECVLGQSKRVD	871.70
HLA-DRB1*07:01	1	571	585	15	FKNHTSPDV	YFKNHTSPDVDLGDI	878.50
HLA-DRB1*07:01	1	415	429	15	NAQALNTLV	QNAQALNTLVKQLSS	893.30
HLA-DRB1*07:01	1	689	703	15	TLIVNSVLL	TGTLIVNSVLLFLAF	936.30
HLA-DRB1*07:01	1	927	941	15	PNNTASWFT	GLPNNTASWFTALTQ	949.20
HLA-DRB1*07:01	1	583	597	15	ISGINASVV	GDISGINASVVNIQK	955.80

HLA-DRB1*09:01

Allele	#	Start	End	Length	Core Sequence	Peptide Sequence	IC50
HLA-DRB1*09:01	1	1096	1110	15	FAPSASAFF	PQIAQFAPSASAFFG	4.70
HLA-DRB1*09:01	1	1095	1109	15	FAPSASAFF	WPQIAQFAPSASAFF	4.80
HLA-DRB1*09:01	1	1097	1111	15	FAPSASAFF	QIAQFAPSASAFFGM	4.80
HLA-DRB1*09:01	1	1098	1112	15	FAPSASAFF	IAQFAPSASAFFGMS	5.10
HLA-DRB1*09:01	1	1100	1114	15	FAPSASAFF	QFAPSASAFFGMSRI	7.40
HLA-DRB1*09:01	1	849	863	15	YKLGASQRV	TLSYYKLGASQRVAG	13.00
HLA-DRB1*09:01	1	850	864	15	YKLGASQRV	LSYYKLGASQRVAGD	13.50
HLA-DRB1*09:01	1	277	291	15	FNGLTGTGV	VNFNFNGLTGTGVLT	14.60
HLA-DRB1*09:01	1	1101	1115	15	FAPSASAFF	FAPSASAFFGMSRIG	15.00
HLA-DRB1*09:01	1	276	290	15	FNGLTGTGV	CVNFNFNGLTGTGVL	16.40
HLA-DRB1*09:01	1	838	852	15	ITVATSRTL	LPKEITVATSRTLSY	37.30
HLA-DRB1*09:01	1	837	851	15	ITVATSRTL	DLPKEITVATSRTLS	37.70
HLA-DRB1*09:01	1	839	853	15	ITVATSRTL	PKEITVATSRTLSYY	39.10
HLA-DRB1*09:01	1	836	850	15	ITVATSRTL	KDLPKEITVATSRTL	41.00
HLA-DRB1*09:01	1	1094	1108	15	IAQFAPSAS	HWPQIAQFAPSASAF	47.30
HLA-DRB1*09:01	1	635	649	15	FIAGLIAIV	WLGFIAGLIAIVMVT	51.50
HLA-DRB1*09:01	1	840	854	15	ITVATSRTL	KEITVATSRTLSYYK	54.40
HLA-DRB1*09:01	1	425	439	15	FGAISSVLN	KQLSSNFGAISSVLN	59.40
HLA-DRB1*09:01	1	1104	1118	15	FGMSRIGME	SASAFFGMSRIGMEV	63.30
HLA-DRB1*09:01	1	636	650	15	FIAGLIAIV	LGFIAGLIAIVMVTI	63.90
HLA-DRB1*09:01	1	1103	1117	15	FGMSRIGME	PSASAFFGMSRIGME	69.90

Allele	#	Start	End	Length	Core Sequence	Peptide Sequence	IC50
HLA-DRB1*09:01	1	378	392	15	AYRFNGIGV	PFAMQMAYRFNGIGV	70.30
HLA-DRB1*09:01	1	379	393	15	AYRFNGIGV	FAMQMAYRFNGIGVT	70.50
HLA-DRB1*09:01	1	1106	1120	15	FGMSRIGME	SAFFGMSRIGMEVTP	72.40
HLA-DRB1*09:01	1	426	440	15	FGAISSVLN	QLSSNFGAISSVLND	73.70
HLA-DRB1*09:01	1	422	436	15	LSSNFGAIS	TLVKQLSSNFGAISS	76.40
HLA-DRB1*09:01	1	421	435	15	LVKQLSSNF	NTLVKQLSSNFGAIS	77.40
HLA-DRB1*09:01	1	841	855	15	ITVATSRTL	EITVATSRTLSYYKL	86.40
HLA-DRB1*09:01	1	424	438	15	LSSNFGAIS	VKQLSSNFGAISSVL	88.80
HLA-DRB1*09:01	1	349	363	15	FNGLTVLPP	CAQKFNGLTVLPPLL	91.50
HLA-DRB1*09:01	1	382	396	15	AYRFNGIGV	QMAYRFNGIGVTQNV	94.60
HLA-DRB1*09:01	1	423	437	15	LSSNFGAIS	LVKQLSSNFGAISSV	95.40
HLA-DRB1*09:01	1	427	441	15	FGAISSVLN	LSSNFGAISSVLNDI	96.40
HLA-DRB1*09:01	1	681	695	15	FVSEETGTL	MYSFVSEETGTLIVN	100.60
HLA-DRB1*09:01	1	381	395	15	AYRFNGIGV	MQMAYRFNGIGVTQN	100.80
HLA-DRB1*09:01	1	792	806	15	FARTRSMWS	FRLFARTRSMWSFNP	108.20
HLA-DRB1*09:01	1	374	388	15	FAMQMAYRF	ALQIPFAMQMAYRFN	111.50
HLA-DRB1*09:01	1	383	397	15	AYRFNGIGV	MAYRFNGIGVTQNVL	112.30
HLA-DRB1*09:01	1	793	807	15	FARTRSMWS	RLFARTRSMWSFNPE	121.30
HLA-DRB1*09:01	1	488	502	15	CVLGQSKRV	TKMSECVLGQSKRVD	127.00
HLA-DRB1*09:01	1	489	503	15	CVLGQSKRV	KMSECVLGQSKRVDF	131.00
HLA-DRB1*09:01	1	375	389	15	FAMQMAYRF	LQIPFAMQMAYRFNG	141.90
HLA-DRB1*09:01	1	348	362	15	FNGLTVLPP	ICAQKFNGLTVLPPL	155.30
HLA-DRB1*09:01	1	420	434	15	LVKQLSSNF	LNTLVKQLSSNFGAI	156.30
HLA-DRB1*09:01	1	490	504	15	CVLGQSKRV	MSECVLGQSKRVDFC	158.50
HLA-DRB1*09:01	1	794	808	15	FARTRSMWS	LFARTRSMWSFNPET	160.70
HLA-DRB1*09:01	1	569	583	15	FKNHTSPDV	DKYFKNHTSPDVDLG	163.40
HLA-DRB1*09:01	1	568	582	15	FKNHTSPDV	LDKYFKNHTSPDVDL	170.30
HLA-DRB1*09:01	1	459	473	15	LQSLQTYVT	TGRLQSLQTYVTQQL	177.70
HLA-DRB1*09:01	1	376	390	15	FAMQMAYRF	QIPFAMQMAYRFNGI	178.20
HLA-DRB1*09:01	1	457	471	15	LQSLQTYVT	LITGRLQSLQTYVTQ	180.70
HLA-DRB1*09:01	1	842	856	15	ITVATSRTL	ITVATSRTLSYYKLG	183.50
HLA-DRB1*09:01	1	960	974	15	FYYLGTGPE	LSPRWYFYYLGTGPE	183.60
HLA-DRB1*09:01	1	377	391	15	FAMQMAYRF	IPFAMQMAYRFNGIG	183.90
HLA-DRB1*09:01	1	570	584	15	FKNHTSPDV	KYFKNHTSPDVDLGD	187.80
HLA-DRB1*09:01	1	456	470	15	LQSLQTYVT	RLITGRLQSLQTYVT	188.80
HLA-DRB1*09:01	1	259	273	15	YQPYRVVVL	GYQPYRVVVLSFELL	190.10
HLA-DRB1*09:01	1	458	472	15	LQSLQTYVT	ITGRLQSLQTYVTQQ	192.60

Allele	#	Start	End	Length	Core Sequence	Peptide Sequence	IC50
HLA-DRB1*09:01	1	795	809	15	TRSMWSFNP	FARTRSMWSFNPETN	194.00
HLA-DRB1*09:01	1	347	361	15	FNGLTVLPP	LICAQKFNGLTVLPP	197.30
HLA-DRB1*09:01	1	460	474	15	LQSLQTYVT	GRLQSLQTYVTQQLI	201.60
HLA-DRB1*09:01	1	418	432	15	LVKQLSSNF	QALNTLVKQLSSNFG	202.20
HLA-DRB1*09:01	1	1108	1122	15	FGMSRIGME	FFGMSRIGMEVTPSG	206.90
HLA-DRB1*09:01	1	430	444	15	FGAISSVLN	NFGAISSVLNDILSR	207.70
HLA-DRB1*09:01	1	1092	1106	15	IAQFAPSAS	YKHWPQIAQFAPSAS	212.60
HLA-DRB1*09:01	1	567	581	15	FKNHTSPDV	ELDKYFKNHTSPDVD	219.10
HLA-DRB1*09:01	1	1113	1127	15	VTPSGTWLT	RIGMEVTPSGTWLTY	249.30
HLA-DRB1*09:01	1	798	812	15	FNPETNILL	TRSMWSFNPETNILL	255.00
HLA-DRB1*09:01	1	888	902	15	FHPLADNKF	GNSPFHPLADNKFAL	273.20
HLA-DRB1*09:01	1	492	506	15	CVLGQSKRV	ECVLGQSKRVDFCGK	284.20
HLA-DRB1*09:01	1	765	779	15	LWLLWPVTL	FLWLLWPVTLACFVL	286.80
HLA-DRB1*09:01	1	682	696	15	FVSEETGTL	YSFVSEETGTLIVNS	289.90
HLA-DRB1*09:01	1	296	310	15	FCTQLNRAL	LLQYGSFCTQLNRAL	301.30
HLA-DRB1*09:01	1	797	811	15	MWSFNPETN	RTRSMWSFNPETNIL	306.70
HLA-DRB1*09:01	1	931	945	15	WFTALTQHG	NTASWFTALTQHGKE	309.00
HLA-DRB1*09:01	1	231	245	15	FASVYAWNR	RFASVYAWNRKRISN	311.30
HLA-DRB1*09:01	1	566	580	15	FKNHTSPDV	EELDKYFKNHTSPDV	325.70
HLA-DRB1*09:01	1	1112	1126	15	VTPSGTWLT	SRIGMEVTPSGTWLT	328.70
HLA-DRB1*09:01	1	930	944	15	WFTALTQHG	NNTASWFTALTQHGK	329.50
HLA-DRB1*09:01	1	571	585	15	FKNHTSPDV	YFKNHTSPDVDLGDI	340.10
HLA-DRB1*09:01	1	461	475	15	LQSLQTYVT	RLQSLQTYVTQQLIR	361.40
HLA-DRB1*09:01	1	618	632	15	QYIKWPWYI	ELGKYEQYIKWPWYI	375.50
HLA-DRB1*09:01	1	327	341	15	FNKVTLADA	EDLLFNKVTLADAGF	385.50
HLA-DRB1*09:01	1	1064	1078	15	YNVTQAFGR	YNVTQAFGRRGPEQT	394.70
HLA-DRB1*09:01	1	846	860	15	YYKLGASQR	TSRTLSYYKLGASQR	401.10
HLA-DRB1*09:01	1	929	943	15	WFTALTQHG	PNNTASWFTALTQHG	401.40
HLA-DRB1*09:01	1	345	359	15	AQKFNGLTV	RDLICAQKFNGLTVL	447.00
HLA-DRB1*09:01	1	886	900	15	FHPLADNKF	YEGNSPFHPLADNKF	448.10
HLA-DRB1*09:01	1	534	548	15	FVSGNCDVV	DNTFVSGNCDVVIGI	449.40
HLA-DRB1*09:01	1	705	719	15	LAILTALRL	VFLLVTLAILTALRL	457.10
HLA-DRB1*09:01	1	533	547	15	FVSGNCDVV	TDNTFVSGNCDVVIG	462.40
HLA-DRB1*09:01	1	346	360	15	AQKFNGLTV	DLICAQKFNGLTVLP	471.60
HLA-DRB1*09:01	1	385	399	15	IGVTQNVLY	YRFNGIGVTQNVLYE	481.40
HLA-DRB1*09:01	1	326	340	15	FNKVTLADA	IEDLLFNKVTLADAG	493.00
HLA-DRB1*09:01	1	532	546	15	FVSGNCDVV	TTDNTFVSGNCDVVI	511.90

Allele	#	Start	End	Length	Core Sequence	Peptide Sequence	IC50
HLA-DRB1*09:01	1	451	465	15	IDRLITGRL	EVQIDRLITGRLQSL	513.90
HLA-DRB1*09:01	1	452	466	15	IDRLITGRL	VQIDRLITGRLQSLQ	519.10
HLA-DRB1*09:01	1	453	467	15	IDRLITGRL	QIDRLITGRLQSLQT	526.10
HLA-DRB1*09:01	1	1109	1123	15	FGMSRIGME	FGMSRIGMEVTPSGT	527.80
HLA-DRB1*09:01	1	455	469	15	RLQSLQTYV	DRLITGRLQSLQTYV	530.00
HLA-DRB1*09:01	1	232	246	15	VYAWNRKRI	FASVYAWNRKRISNC	556.60
HLA-DRB1*09:01	1	414	428	15	ALNTLVKQL	NQNAQALNTLVKQLS	606.70
HLA-DRB1*09:01	1	713	727	15	LCAYCCNIV	ILTALRLCAYCCNIV	642.00
HLA-DRB1*09:01	1	415	429	15	ALNTLVKQL	QNAQALNTLVKQLSS	645.90
HLA-DRB1*09:01	1	583	597	15	ISGINASVV	GDISGINASVVNIQK	653.60
HLA-DRB1*09:01	1	449	463	15	IDRLITGRL	EAEVQIDRLITGRLQ	658.70
HLA-DRB1*09:01	1	431	445	15	FGAISSVLN	FGAISSVLNDILSRL	666.30
HLA-DRB1*09:01	1	582	596	15	ISGINASVV	LGDISGINASVVNIQ	666.50
HLA-DRB1*09:01	1	715	729	15	LCAYCCNIV	TALRLCAYCCNIVNV	674.40
HLA-DRB1*09:01	1	714	728	15	LCAYCCNIV	LTALRLCAYCCNIVN	686.00
HLA-DRB1*09:01	1	1175	1189	15	FHNIRGRWT	KQRFHNIRGRWTGDY	693.00
HLA-DRB1*09:01	1	454	468	15	IDRLITGRL	IDRLITGRLQSLQTY	701.00
HLA-DRB1*09:01	1	801	815	15	FNPETNILL	MWSFNPETNILLNVP	719.60
HLA-DRB1*09:01	1	706	720	15	LAILTALRL	FLLVTLAILTALRLC	720.50
HLA-DRB1*09:01	1	1174	1188	15	FHNIRGRWT	KKQRFHNIRGRWTGD	721.30
HLA-DRB1*09:01	1	413	427	15	ALNTLVKQL	VNQNAQALNTLVKQL	722.70
HLA-DRB1*09:01	1	1173	1187	15	FHNIRGRWT	DKKQRFHNIRGRWTG	749.90
HLA-DRB1*09:01	1	987	1001	15	FYAEGSRGG	LPKGFYAEGSRGGSQ	759.40
HLA-DRB1*09:01	1	410	424	15	VVNQNAQAL	QDVVNQNAQALNTLV	776.60
HLA-DRB1*09:01	1	493	507	15	CVLGQSKRV	CVLGQSKRVDFCGKG	784.60
HLA-DRB1*09:01	1	584	598	15	ISGINASVV	DISGINASVVNIQKE	800.40
HLA-DRB1*09:01	1	1136	1150	15	YKTFPPTEP	HIDAYKTFPPTEPKK	802.30
HLA-DRB1*09:01	1	710	724	15	LAILTALRL	TLAILTALRLCAYCC	816.20
HLA-DRB1*09:01	1	1135	1149	15	YKTFPPTEP	KHIDAYKTFPPTEPK	820.90
HLA-DRB1*09:01	1	1137	1151	15	YKTFPPTEP	IDAYKTFPPTEPKKD	842.90
HLA-DRB1*09:01	1	718	732	15	LCAYCCNIV	RLCAYCCNIVNVSLV	853.80
HLA-DRB1*09:01	1	411	425	15	VVNQNAQAL	DVVNQNAQALNTLVK	858.90
HLA-DRB1*09:01	1	802	816	15	FNPETNILL	WSFNPETNILLNVPL	868.40
HLA-DRB1*09:01	1	531	545	15	FVSGNCDVV	ITTDNTFVSGNCDVV	871.30
HLA-DRB1*09:01	1	416	430	15	ALNTLVKQL	NAQALNTLVKQLSSN	873.30
HLA-DRB1*09:01	1	684	698	15	FVSEETGTL	FVSEETGTLIVNSVL	877.60
HLA-DRB1*09:01	1	581	595	15	ISGINASVV	DLGDISGINASVVNI	882.20

Allele	#	Start	End	Length	Core Sequence	Peptide Sequence	IC50
HLA-DRB1*09:01	1	1176	1190	15	FHNIRGRWT	QRFHNIRGRWTGDYK	883.30
HLA-DRB1*09:01	1	386	400	15	IGVTQNVLY	RFNGIGVTQNVLYEN	902.10
HLA-DRB1*09:01	1	989	1003	15	FYAEGSRGG	KGFYAEGSRGGSQAS	908.50
HLA-DRB1*09:01	1	708	722	15	LAILTALRL	LVTLAILTALRLCAY	919.00
HLA-DRB1*09:01	1	709	723	15	LAILTALRL	VTLAILTALRLCAYC	936.70
HLA-DRB1*09:01	1	294	308	15	YGSFCTQLN	NLLLQYGSFCTQLNR	957.90
HLA-DRB1*09:01	1	603	617	15	AKNLNESLI	NEVAKNLNESLIDLQ	983.10
HLA-DRB1*09:01	1	1138	1152	15	YKTFPPTEP	DAYKTFPPTEPKKDK	983.50
HLA-DRB1*09:01	1	927	941	15	ASWFTALTQ	GLPNNTASWFTALTQ	984.50

HLA-DRB1*13:02

Allele	#	Start	End	Length	Core Sequence	Peptide Sequence	IC50
HLA-DRB1*13:02	1	582	596	15	ISGINASVV	LGDISGINASVVNIQ	12.00
HLA-DRB1*13:02	1	324	338	15	LLFNKVTLA	SFIEDLLFNKVTLAD	12.80
HLA-DRB1*13:02	1	583	597	15	ISGINASVV	GDISGINASVVNIQK	13.80
HLA-DRB1*13:02	1	326	340	15	LLFNKVTLA	IEDLLFNKVTLADAG	14.60
HLA-DRB1*13:02	1	581	595	15	ISGINASVV	DLGDISGINASVVNI	15.90
HLA-DRB1*13:02	1	409	423	15	VVNQNAQAL	LQDVVNQNAQALNTL	16.80
HLA-DRB1*13:02	1	408	422	15	VVNQNAQAL	KLQDVVNQNAQALNT	18.50
HLA-DRB1*13:02	1	687	701	15	LIVNSVLLF	EETGTLIVNSVLLFL	19.20
HLA-DRB1*13:02	1	584	598	15	INASVVNIQ	DISGINASVVNIQKE	19.60
HLA-DRB1*13:02	1	407	421	15	VVNQNAQAL	GKLQDVVNQNAQALN	19.80
HLA-DRB1*13:02	1	686	700	15	LIVNSVLLF	SEETGTLIVNSVLLF	20.90
HLA-DRB1*13:02	1	410	424	15	VVNQNAQAL	QDVVNQNAQALNTLV	21.50
HLA-DRB1*13:02	1	327	341	15	LLFNKVTLA	EDLLFNKVTLADAGF	22.40
HLA-DRB1*13:02	1	689	703	15	LIVNSVLLF	TGTLIVNSVLLFLAF	22.90
HLA-DRB1*13:02	1	580	594	15	ISGINASVV	VDLGDISGINASVVN	25.80
HLA-DRB1*13:02	1	839	853	15	ITVATSRTL	PKEITVATSRTLSYY	26.30
HLA-DRB1*13:02	1	406	420	15	VVNQNAQAL	LGKLQDVVNQNAQAL	27.30
HLA-DRB1*13:02	1	838	852	15	ITVATSRTL	LPKEITVATSRTLSY	30.00
HLA-DRB1*13:02	1	840	854	15	ITVATSRTL	KEITVATSRTLSYYK	30.10
HLA-DRB1*13:02	1	602	616	15	VAKNLNESL	LNEVAKNLNESLIDL	30.60
HLA-DRB1*13:02	1	579	593	15	ISGINASVV	DVDLGDISGINASVV	31.10
HLA-DRB1*13:02	1	585	599	15	INASVVNIQ	ISGINASVVNIQKEI	34.40
HLA-DRB1*13:02	1	600	614	15	VAKNLNESL	DRLNEVAKNLNESLI	36.20
HLA-DRB1*13:02	1	690	704	15	LIVNSVLLF	GTLIVNSVLLFLAFV	36.40

Allele	#	Start	End	Length	Core Sequence	Peptide Sequence	IC50
HLA-DRB1*13:02	1	601	615	15	VAKNLNESL	RLNEVAKNLNESLID	36.80
HLA-DRB1*13:02	1	411	425	15	VVNQNAQAL	DVVNQNAQALNTLVK	41.10
HLA-DRB1*13:02	1	421	435	15	LSSNFGAIS	NTLVKQLSSNFGAIS	41.70
HLA-DRB1*13:02	1	603	617	15	VAKNLNESL	NEVAKNLNESLIDLQ	41.70
HL-DRB1*13:02	1	841	855	15	VTSRTLSY	EITVTSRTLSYYKL	47.10
HLA-DRB1*13:02	1	422	436	15	LSSNFGAIS	TLVKQLSSNFGAISS	47.70
HLA-DRB1*13:02	1	431	445	15	VLNDILSRL	FGAISSVLNDILSRL	48.30
HLA-DRB1*13:02	1	423	437	15	LSSNFGAIS	LVKQLSSNFGAISSV	51.00
HLA-DRB1*13:02	1	424	438	15	LSSNFGAIS	VKQLSSNFGAISSVL	54.00
HLA-DRB1*13:02	1	599	613	15	VAKNLNESL	IDRLNEVAKNLNESL	54.30
HLA-DRB1*13:02	1	434	448	15	VLNDILSRL	ISSVLNDILSRLDKV	55.40
HLA-DRB1*13:02	1	432	446	15	VLNDILSRL	GAISSVLNDILSRLD	58.50
HLA-DRB1*13:02	1	420	434	15	LVKQLSSNF	LNTLVKQLSSNFGAI	69.00
HLA-DRB1*13:02	1	837	851	15	ITVATSRTL	DLPKEITVATSRTLS	77.30
HLA-DRB1*13:02	1	691	705	15	LIVNSVLLF	TLIVNSVLLFLAFVV	85.80
HLA-DRB1*13:02	1	534	548	15	VSGNCDVVI	DNTFVSGNCDVVIGI	86.30
HLA-DRB1*13:02	1	425	439	15	LSSNFGAIS	KQLSSNFGAISSVLN	86.50
HLA-DRB1*13:02	1	836	850	15	ITVATSRTL	KDLPKEITVATSRTL	87.80
HLA-DRB1*13:02	1	529	543	15	ITTDNTFVS	QIITTDNTFVSGNCD	91.60
HLA-DRB1*13:02	1	604	618	15	VAKNLNESL	EVAKNLNESLIDLQE	98.30
HLA-DRB1*13:02	1	427	441	15	AISSVLNDI	LSSNFGAISSVLNDI	100.00
HLA-DRB1*13:02	1	376	390	15	FAMQMAYRF	QIPFAMQMAYRFNGI	100.10
HLA-DRB1*13:02	1	532	546	15	VSGNCDVVI	TTDNTFVSGNCDVVI	101.80
HLA-DRB1*13:02	1	451	465	15	IDRLITGRL	EVQIDRLITGRLQSL	111.20
HLA-DRB1*13:02	1	533	547	15	VSGNCDVVI	TDNTFVSGNCDVVIG	112.80
HLA-DRB1*13:02	1	412	426	15	VVNQNAQAL	VVNQNAQALNTLVKQ	124.50
HLA-DRB1*13:02	1	238	252	15	RISNCVADY	WNRKRISNCVADYSV	131.20
HLA-DRB1*13:02	1	379	393	15	YRFNGIGVT	FAMQMAYRFNGIGVT	131.80
HLA-DRB1*13:02	1	1130	1144	15	ILLNKHIDA	VILLNKHIDAYKTFP	132.50
HLA-DRB1*13:02	1	1095	1109	15	FAPSASAFF	WPQIAQFAPSASAFF	133.20
HLA-DRB1*13:02	1	239	253	15	RISNCVADY	NRKRISNCVADYSVL	133.90
HLA-DRB1*13:02	1	236	250	15	RISNCVADY	YAWNRKRISNCVADY	140.90
HLA-DRB1*13:02	1	1096	1110	15	FAPSASAFF	PQIAQFAPSASAFFG	145.20
HLA-DRB1*13:02	1	1097	1111	15	FAPSASAFF	QIAQFAPSASAFFGM	145.30
HLA-DRB1*13:02	1	375	389	15	FAMQMAYRF	LQIPFAMQMAYRFNG	149.50
HLA-DRB1*13:02	1	595	609	15	DRLNEVAKN	IQKEIDRLNEVAKNL	149.80
HLA-DRB1*13:02	1	385	399	15	IGVTQNVLY	YRFNGIGVTQNVLYE	152.20

Allele	#	Start	End	Length	Core Sequence	Peptide Sequence	IC50
HLA-DRB1*13:02	1	449	463	15	IDRLITGRL	EAEVQIDRLITGRLQ	164.20
HLA-DRB1*13:02	1	452	466	15	IDRLITGRL	VQIDRLITGRLQSLQ	165.00
HLA-DRB1*13:02	1	241	255	15	CVADYSVLY	KRISNCVADYSVLYN	166.10
HLA-DRB1*13:02	1	382	396	15	YRFNGIGVT	QMAYRFNGIGVTQNV	171.10
HLA-DRB1*13:02	1	842	856	15	VATSRTLSY	ITVATSRTLSYYKLG	171.30
HLA-DRB1*13:02	1	1098	1112	15	FAPSASAFF	IAQFAPSASAFFGMS	173.60
HLA-DRB1*13:02	1	596	610	15	DRLNEVAKN	QKEIDRLNEVAKNLN	174.80
HLA-DRB1*13:02	1	377	391	15	FAMQMAYRF	IPFAMQMAYRFNGIG	176.10
HLA-DRB1*13:02	1	378	392	15	FAMQMAYRF	PFAMQMAYRFNGIGV	177.30
HLA-DRB1*13:02	1	594	608	15	DRLNEVAKN	NIQKEIDRLNEVAKN	178.60
HLA-DRB1*13:02	1	381	395	15	YRFNGIGVT	MQMAYRFNGIGVTQN	184.40
HLA-DRB1*13:02	1	386	400	15	IGVTQNVLY	RFNGIGVTQNVLYEN	186.30
HLA-DRB1*13:02	1	448	462	15	IDRLITGRL	VEAEVQIDRLITGRL	199.10
HLA-DRB1*13:02	1	387	401	15	IGVTQNVLY	FNGIGVTQNVLYENQ	206.30
HLA-DRB1*13:02	1	426	440	15	LSSNFGAIS	QLSSNFGAISSVLND	212.20
HLA-DRB1*13:02	1	383	397	15	YRFNGIGVT	MAYRFNGIGVTQNVL	232.10
HLA-DRB1*13:02	1	597	611	15	DRLNEVAKN	KEIDRLNEVAKNLNE	239.10
HLA-DRB1*13:02	1	635	649	15	FIAGLIAIV	WLGFIAGLIAIVMVT	255.70
HLA-DRB1*13:02	1	388	402	15	IGVTQNVLY	NGIGVTQNVLYENQK	265.70
HLA-DRB1*13:02	1	849	863	15	YKLGASQRV	TLSYYKLGASQRVAG	266.70
HLA-DRB1*13:02	1	925	939	15	LPNNTASWF	PQGLPNNTASWFTAL	268.90
HLA-DRB1*13:02	1	453	467	15	LITGRLQSL	QIDRLITGRLQSLQT	269.60
HLA-DRB1*13:02	1	530	544	15	ITTDNTFVS	IITTDNTFVSGNCDV	281.80
HLA-DRB1*13:02	1	923	937	15	GLPNNTASW	RRPQGLPNNTASWFT	293.20
HLA-DRB1*13:02	1	850	864	15	YKLGASQRV	LSYYKLGASQRVAGD	295.50
HLA-DRB1*13:02	1	345	359	15	ICAQKFNGL	RDLICAQKFNGLTVL	300.40
HLA-DRB1*13:02	1	801	815	15	WSFNPETNI	MWSFNPETNILLNVP	304.10
HLA-DRB1*13:02	1	437	451	15	DILSRLDKV	VLNDILSRLDKVEAE	320.30
HLA-DRB1*13:02	1	242	256	15	CVADYSVLY	RISNCVADYSVLYNS	320.70
HLA-DRB1*13:02	1	922	936	15	GLPNNTASW	QRRPQGLPNNTASWF	340.20
HLA-DRB1*13:02	1	598	612	15	DRLNEVAKN	EIDRLNEVAKNLNES	343.50
HLA-DRB1*13:02	1	802	816	15	PETNILLNV	WSFNPETNILLNVPL	356.30
HLA-DRB1*13:02	1	692	706	15	LIVNSVLLF	LIVNSVLLFLAFVVF	363.10
HLA-DRB1*13:02	1	636	650	15	FIAGLIAIV	LGFIAGLIAIVMVTI	366.70
HLA-DRB1*13:02	1	418	432	15	LVKQLSSNF	QALNTLVKQLSSNFG	367.10
HLA-DRB1*13:02	1	926	940	15	LPNNTASWF	QGLPNNTASWFTALT	369.60
HLA-DRB1*13:02	1	1131	1145	15	ILLNKHIDA	ILLNKHIDAYKTFPP	391.30

Allele	#	Start	End	Length	Core Sequence	Peptide Sequence	IC50
HLA-DRB1*13:02	1	832	846	15	DIKDLPKEI	RCDIKDLPKEITVAT	434.60
HLA-DRB1*13:02	1	346	360	15	QKFNGLTVL	DLICAQKFNGLTVLP	435.60
HLA-DRB1*13:02	1	798	812	15	WSFNPETNI	TRSMWSFNPETNILL	454.30
HLA-DRB1*13:02	1	413	427	15	QALNTLVKQ	VNQNAQALNTLVKQL	469.60
HLA-DRB1*13:02	1	685	699	15	TLIVNSVLL	VSEETGTLIVNSVLL	470.70
HLA-DRB1*13:02	1	1113	1127	15	VTPSGTWLT	RIGMEVTPSGTWLTY	518.10
HLA-DRB1*13:02	1	593	607	15	KEIDRLNEV	VNIQKEIDRLNEVAK	518.70
HLA-DRB1*13:02	1	588	602	15	INASVVNIQ	INASVVNIQKEIDRL	526.10
HLA-DRB1*13:02	1	454	468	15	LITGRLQSL	IDRLITGRLQSLQTY	531.80
HLA-DRB1*13:02	1	210	224	15	RFPNITNLC	VRFPNITNLCPFGEV	539.30
HLA-DRB1*13:02	1	568	582	15	FKNHTSPDV	LDKYFKNHTSPDVDL	549.60
HLA-DRB1*13:02	1	605	619	15	VAKNLNESL	VAKNLNESLIDLQEL	561.90
HLA-DRB1*13:02	1	347	361	15	QKFNGLTVL	LICAQKFNGLTVLPP	577.50
HLA-DRB1*13:02	1	276	290	15	VNFNFNGLT	CVNFNFNGLTGTGVL	605.70
HLA-DRB1*13:02	1	414	428	15	QALNTLVKQ	NQNAQALNTLVKQLS	640.10
HLA-DRB1*13:02	1	235	249	15	NRKRISNCV	VYAWNRKRISNCVAD	656.90
HLA-DRB1*13:02	1	349	363	15	QKFNGLTVL	CAQKFNGLTVLPPLL	660.20
HLA-DRB1*13:02	1	569	583	15	FKNHTSPDV	DKYFKNHTSPDVDLG	670.00
HLA-DRB1*13:02	1	719	733	15	YCCNIVNVS	LCAYCCNIVNVSLVK	671.40
HLA-DRB1*13:02	1	833	847	15	DIKDLPKEI	CDIKDLPKEITVATS	680.30
HLA-DRB1*13:02	1	531	545	15	FVSGNCDVV	ITTDNTFVSGNCDVV	683.50
HLA-DRB1*13:02	1	705	719	15	LAILTALRL	VFLLVTLAILTALRL	688.70
HLA-DRB1*13:02	1	567	581	15	FKNHTSPDV	ELDKYFKNHTSPDVD	696.40
HLA-DRB1*13:02	1	566	580	15	YFKNHTSPD	EELDKYFKNHTSPDV	725.60
HLA-DRB1*13:02	1	348	362	15	QKFNGLTVL	ICAQKFNGLTVLPPL	748.70
HLA-DRB1*13:02	1	718	732	15	YCCNIVNVS	RLCAYCCNIVNVSLV	756.10
HLA-DRB1*13:02	1	717	731	15	YCCNIVNVS	LRLCAYCCNIVNVSL	761.00
HLA-DRB1*13:02	1	797	811	15	WSFNPETNI	RTRSMWSFNPETNIL	764.50
HLA-DRB1*13:02	1	1100	1114	15	FAPSASAFF	QFAPSASAFFGMSRI	778.50
HLA-DRB1*13:02	1	792	806	15	FARTRSMWS	FRLFARTRSMWSFNP	787.70
HLA-DRB1*13:02	1	415	429	15	QALNTLVKQ	QNAQALNTLVKQLSS	814.90
HLA-DRB1*13:02	1	681	695	15	VSEETGTLI	MYSFVSEETGTLIVN	818.60
HLA-DRB1*13:02	1	682	696	15	VSEETGTLI	YSFVSEETGTLIVNS	845.50
HLA-DRB1*13:02	1	277	291	15	FNGLTGTGV	VNFNFNGLTGTGVLT	890.40
HLA-DRB1*13:02	1	570	584	15	FKNHTSPDV	KYFKNHTSPDVDLGD	890.40
HLA-DRB1*13:02	1	715	729	15	LCAYCCNIV	TALRLCAYCCNIVNV	897.10
HLA-DRB1*13:02	1	489	503	15	CVLGQSKRV	KMSECVLGQSKRVDF	901.90

Allele	#	Start	End	Length	Core Sequence	Peptide Sequence	IC50
HLA-DRB1*13:02	1	438	452	15	DILSRLDKV	LNDILSRLDKVEAEV	938.80

HLA-DRB1*15:01

Allele	#	Start	End	Length	Core Sequence	Peptide Sequence	IC50
HL-DRB1*15:01	1	294	308	15	LLQYGSFCT	NLLLQYGSFCTQLNR	12.10
HLA-DRB1*15:01	1	460	474	15	LQSLQTYVT	GRLQSLQTYVTQQLI	15.70
HLA-DRB1*15:01	1	459	473	15	LQSLQTYVT	TGRLQSLQTYVTQQL	16.80
HLA-DRB1*15:01	1	461	475	15	LQTYVTQQL	RLQSLQTYVTQQLIR	22.10
HLA-DRB1*15:01	1	421	435	15	VKQLSSNFG	NTLVKQLSSNFGAIS	25.60
HLA-DRB1*15:01	1	420	434	15	VKQLSSNFG	LNTLVKQLSSNFGAI	26.30
HLA-DRB1*15:01	1	458	472	15	LQSLQTYVT	ITGRLQSLQTYVTQQ	31.80
HLA-DRB1*15:01	1	422	436	15	VKQLSSNFG	TLVKQLSSNFGAISS	39.00
HLA-DRB1*15:01	1	1132	1146	15	IDAYKTFPP	LLNKHIDAYKTFPPT	43.30
HLA-DRB1*15:01	1	457	471	15	LQSLQTYVT	LITGRLQSLQTYVTQ	44.50
HLA-DRB1*15:01	1	1133	1147	15	IDAYKTFPP	LNKHIDAYKTFPPTE	49.50
HLA-DRB1*15:01	1	846	860	15	LSYYKLGAS	TSRTLSYYKLGASQR	52.60
HLA-DRB1*15:01	1	1134	1148	15	IDAYKTFPP	NKHIDAYKTFPPTEP	57.80
HLA-DRB1*15:01	1	1131	1145	15	IDAYKTFPP	ILLNKHIDAYKTFPP	61.60
HL-DRB1*15:01	1	1135	1149	15	IDAYKTFPP	KHIDAYKTFPPTEPK	72.80
HLA-DRB1*15:01	1	714	728	15	ALRLCAYCC	LTALRLCAYCCNIVN	78.20
HLA-DRB1*15:01	1	715	729	15	LCAYCCNIV	TALRLCAYCCNIVNV	80.90
HLA-DRB1*15:01	1	844	858	15	TLSYYKLGA	VATSRTLSYYKLGAS	82.60
HLA-DRB1*15:01	1	710	724	15	LAILTALRL	TLAILTALRLCAYCC	82.80
HLA-DRB1*15:01	1	709	723	15	LAILTALRL	VTLAILTALRLCAYC	83.90
HLA-DRB1*15:01	1	708	722	15	LAILTALRL	LVTLAILTALRLCAY	85.40
HLA-DRB1*15:01	1	456	470	15	LQSLQTYVT	RLITGRLQSLQTYVT	85.60
HLA-DRB1*15:01	1	452	466	15	IDRLITGRL	VQIDRLITGRLQSLQ	89.20
HLA-DRB1*15:01	1	418	432	15	VKQLSSNFG	QALNTLVKQLSSNFG	91.80
HLA-DRB1*15:01	1	453	467	15	IDRLITGRL	QIDRLITGRLQSLQT	92.50
HLA-DRB1*15:01	1	296	310	15	LLQYGSFCT	LLQYGSFCTQLNRAL	93.90
HLA-DRB1*15:01	1	451	465	15	IDRLITGRL	EVQIDRLITGRLQSL	94.40
HLA-DRB1*15:01	1	713	727	15	ALRLCAYCC	ILTALRLCAYCCNIV	96.70
HLA-DRB1*15:01	1	378	392	15	FAMQMAYRF	PFAMQMAYRFNGIGV	99.60
HLA-DRB1*15:01	1	618	632	15	YEQYIKWPW	ELGKYEQYIKWPWYI	114.30
HLA-DRB1*15:01	1	1130	1144	15	NKHIDAYKT	VILLNKHIDAYKTFP	117.10
HLA-DRB1*15:01	1	376	390	15	FAMQMAYRF	QIPFAMQMAYRFNGI	122.50

Allele	#	Start	End	Length	Core Sequence	Peptide Sequence	IC50
HLA-DRB1*15:01	1	843	857	15	SRTLSYYKL	TVATSRTLSYYKLGA	125.20
HLA-DRB1*15:01	1	960	974	15	PRWYFYYLG	LSPRWYFYYLGTGPE	126.40
HLA-DRB1*15:01	1	840	854	15	ITVATSRTL	KEITVATSRTLSYYK	128.80
HLA-DRB1*15:01	1	375	389	15	FAMQMAYRF	LQIPFAMQMAYRFNG	134.70
HLA-DRB1*15:01	1	259	273	15	RVVVLSFEL	GYQPYRVVVLSFELL	136.20
HLA-DRB1*15:01	1	423	437	15	VKQLSSNFG	LVKQLSSNFGAISSV	141.80
HLA-DRB1*15:01	1	454	468	15	IDRLITGRL	IDRLITGRLQSLQTY	150.90
HLA-DRB1*15:01	1	377	391	15	FAMQMAYRF	IPFAMQMAYRFNGIG	151.10
HLA-DRB1*15:01	1	841	855	15	ITVATSRTL	EITVATSRTLSYYKL	151.80
HLA-DRB1*15:01	1	706	720	15	LAILTALRL	FLLVTLAILTALRLC	156.90
HLA-DRB1*15:01	1	839	853	15	ITVATSRTL	PKEITVATSRTLSYY	159.20
HLA-DRB1*15:01	1	711	725	15	ALRLCAYCC	LAILTALRLCAYCCN	160.70
HLA-DRB1*15:01	1	379	393	15	AYRFNGIGV	FAMQMAYRFNGIGVT	168.00
HLA-DRB1*15:01	1	449	463	15	IDRLITGRL	EAEVQIDRLITGRLQ	183.10
HLA-DRB1*15:01	1	1096	1110	15	IAQFAPSAS	PQIAQFAPSASAFFG	191.20
HLA-DRB1*15:01	1	1095	1109	15	IAQFAPSAS	WPQIAQFAPSASAFF	191.50
HLA-DRB1*15:01	1	231	245	15	FASVYAWNR	RFASVYAWNRKRISN	192.20
HLA-DRB1*15:01	1	1136	1150	15	IDAYKTFPP	HIDAYKTFPPTEPKK	199.30
HLA-DRB1*15:01	1	705	719	15	LAILTALRL	VFLLVTLAILTALRL	209.10
HLA-DRB1*15:01	1	842	856	15	TSRTLSYYK	ITVATSRTLSYYKLG	215.30
HLA-DRB1*15:01	1	838	852	15	ITVATSRTL	LPKEITVATSRTLSY	224.90
HLA-DRB1*15:01	1	849	863	15	YKLGASQRV	TLSYYKLGASQRVAG	232.00
HLA-DRB1*15:01	1	617	631	15	LGKYEQYIK	QELGKYEQYIKWPWY	233.10
HLA-DRB1*15:01	1	381	395	15	AYRFNGIGV	MQMAYRFNGIGVTQN	262.50
HLA-DRB1*15:01	1	1097	1111	15	IAQFAPSAS	QIAQFAPSASAFFGM	269.90
HLA-DRB1*15:01	1	689	703	15	TLIVNSVLL	TGTLIVNSVLLFLAF	277.20
HLA-DRB1*15:01	1	616	630	15	LGKYEQYIK	LQELGKYEQYIKWPW	285.70
HLA-DRB1*15:01	1	687	701	15	TLIVNSVLL	EETGTLIVNSVLLFL	288.90
HLA-DRB1*15:01	1	717	731	15	LCAYCCNIV	LRLCAYCCNIVNVSL	301.00
HLA-DRB1*15:01	1	690	704	15	TLIVNSVLL	GTLIVNSVLLFLAFV	307.20
HLA-DRB1*15:01	1	382	396	15	AYRFNGIGV	QMAYRFNGIGVTQNV	320.20
HLA-DRB1*15:01	1	718	732	15	LCAYCCNIV	RLCAYCCNIVNVSLV	322.50
HLA-DRB1*15:01	1	792	806	15	FARTRSMWS	FRLFARTRSMWSFNP	324.90
HLA-DRB1*15:01	1	1094	1108	15	IAQFAPSAS	HWPQIAQFAPSASAF	326.10
HLA-DRB1*15:01	1	345	359	15	ICAQKFNGL	RDLICAQKFNGLTVL	330.00
HLA-DRB1*15:01	1	455	469	15	ITGRLQSLQ	DRLITGRLQSLQTYV	403.50
HLA-DRB1*15:01	1	448	462	15	IDRLITGRL	VEAEVQIDRLITGRL	403.90

Allele	#	Start	End	Length	Core Sequence	Peptide Sequence	IC50
HLA-DRB1*15:01	1	837	851	15	ITVATSRTL	DLPKEITVATSRTLS	408.40
HLA-DRB1*15:01	1	765	779	15	LWLLWPVTL	FLWLLWPVTLACFVL	421.10
HLA-DRB1*15:01	1	793	807	15	FARTRSMWS	RLFARTRSMWSFNPE	425.50
HLA-DRB1*15:01	1	660	674	15	VLKGVKLHY	DDSEPVLKGVKLHYR	442.90
HLA-DRB1*15:01	1	346	360	15	ICAQKFNGL	DLICAQKFNGLTVLP	503.00
HLA-DRB1*15:01	1	691	705	15	VLLFLAFVV	TLIVNSVLLFLAFVV	504.80
HLA-DRB1*15:01	1	1015	1029	15	LLLLDRLNQ	LALLLLDRLNQLESK	528.30
HLA-DRB1*15:01	1	383	397	15	AYRFNGIGV	MAYRFNGIGVTQNVL	574.40
HLA-DRB1*15:01	1	850	864	15	YKLGASQRV	LSYYKLGASQRVAGD	575.60
HLA-DRB1*15:01	1	794	808	15	TRSMWSFNP	LFARTRSMWSFNPET	577.00
HLA-DRB1*15:01	1	232	246	15	VYAWNRKRI	FASVYAWNRKRISNC	590.90
HLA-DRB1*15:01	1	347	361	15	AQKFNGLTV	LICAQKFNGLTVLPP	602.10
HLA-DRB1*15:01	1	802	816	15	FNPETNILL	WSFNPETNILLNVPL	606.70
HLA-DRB1*15:01	1	615	629	15	LGKYEQYIK	DLQELGKYEQYIKWP	644.20
HLA-DRB1*15:01	1	567	581	15	DKYFKNHTS	ELDKYFKNHTSPDVD	650.60
HLA-DRB1*15:01	1	1173	1187	15	FHNIRGRWT	DKKQRFHNIRGRWTG	686.50
HLA-DRB1*15:01	1	693	707	15	VLLFLAFVV	IVNSVLLFLAFVVFL	688.70
HLA-DRB1*15:01	1	1098	1112	15	FAPSASAFF	IAQFAPSASAFFGMS	692.50
HLA-DRB1*15:01	1	424	438	15	LSSNFGAIS	VKQLSSNFGAISSVL	698.80
HLA-DRB1*15:01	1	692	706	15	VLLFLAFVV	LIVNSVLLFLAFVVF	732.00
HLA-DRB1*15:01	1	694	708	15	VLLFLAFVV	VNSVLLFLAFVVFLL	750.90
HLA-DRB1*15:01	1	564	578	15	DKYFKNHTS	FKEELDKYFKNHTSP	756.10
HLA-DRB1*15:01	1	1174	1188	15	FHNIRGRWT	KKQRFHNIRGRWTGD	761.80
HLA-DRB1*15:01	1	563	577	15	DKYFKNHTS	SFKEELDKYFKNHTS	767.80
HLA-DRB1*15:01	1	685	699	15	TLIVNSVLL	VSEETGTLIVNSVLL	791.40
HLA-DRB1*15:01	1	1175	1189	15	FHNIRGRWT	KQRFHNIRGRWTGDY	803.80
HLA-DRB1*15:01	1	565	579	15	DKYFKNHTS	KEELDKYFKNHTSPD	813.90
HLA-DRB1*15:01	1	1104	1118	15	FFGMSRIGM	SASAFFGMSRIGMEV	843.30
HLA-DRB1*15:01	1	614	628	15	LGKYEQYIK	IDLQELGKYEQYIKW	848.40
HLA-DRB1*15:01	1	719	733	15	LCAYCCNIV	LCAYCCNIVNVSLVK	854.00
HLA-DRB1*15:01	1	1100	1114	15	FAPSASAFF	QFAPSASAFFGMSRI	887.00
HLA-DRB1*15:01	1	695	709	15	VLLFLAFVV	NSVLLFLAFVVFLLV	906.00
HLA-DRB1*15:01	1	795	809	15	TRSMWSFNP	FARTRSMWSFNPETN	906.60
HLA-DRB1*15:01	1	384	398	15	IGVTQNVLY	AYRFNGIGVTQNVLY	932.80
HLA-DRB1*15:01	1	349	363	15	AQKFNGLTV	CAQKFNGLTVLPPLL	933.00
HLA-DRB1*15:01	1	568	582	15	FKNHTSPDV	LDKYFKNHTSPDVDL	939.40
HLA-DRB1*15:01	1	1103	1117	15	FFGMSRIGM	PSASAFFGMSRIGME	957.90

Allele	#	Start	End	Length	Core Sequence	Peptide Sequence	IC50
HLA-DRB1*15:01	1	432	446	15	ISSVLNDIL	GAISSVLNDILSRLD	961.90
HLA-DRB1*15:01	1	348	362	15	AQKFNGLTV	ICAQKFNGLTVLPPL	964.30
HLA-DRB1*15:01	1	798	812	15	FNPETNILL	TRSMWSFNPETNILL	983.20

HLA-DRB5*01:01

Allele	#	Start	End	Length	Core Sequence	Peptide Sequence	IC50
HLA-DRB5*01:01	1	231	245	15	FASVYAWNR	RFASVYAWNRKRISN	8.00
HLA-DRB5*01:01	1	375	389	15	FAMQMAYRF	LQIPFAMQMAYRFNG	15.90
HLA-DRB5*01:01	1	376	390	15	FAMQMAYRF	QIPFAMQMAYRFNGI	17.20
HLA-DRB5*01:01	1	232	246	15	FASVYAWNR	FASVYAWNRKRISNC	19.90
HLA-DRB5*01:01	1	849	863	15	YYKLGASQR	TLSYYKLGASQRVAG	20.70
HLA-DRB5*01:01	1	850	864	15	YYKLGASQR	LSYYKLGASQRVAGD	21.30
HLA-DRB5*01:01	1	377	391	15	FAMQMAYRF	IPFAMQMAYRFNGIG	23.40
HLA-DRB5*01:01	1	931	945	15	FTALTQHGK	NTASWFTALTQHGKE	31.60
HLA-DRB5*01:01	1	847	861	15	YYKLGASQR	SRTLSYYKLGASQRV	35.40
HLA-DRB5*01:01	1	378	392	15	FAMQMAYRF	PFAMQMAYRFNGIGV	35.90
HLA-DRB5*01:01	1	791	805	15	FARTRSMWS	SFRLFARTRSMWSFN	38.30
HLA-DRB5*01:01	1	841	855	15	TSRTLSYYK	EITVATSRTLSYYKL	49.00
HLA-DRB5*01:01	1	705	719	15	LAILTALRL	VFLLVTLAILTALRL	51.80
HLA-DRB5*01:01	1	1174	1188	15	FHNIRGRWT	KKQRFHNIRGRWTGD	52.10
HLA-DRB5*01:01	1	708	722	15	LAILTALRL	LVTLAILTALRLCAY	54.50
HLA-DRB5*01:01	1	706	720	15	LAILTALRL	FLLVTLAILTALRLC	54.80
HLA-DRB5*01:01	1	1098	1112	15	FAPSASAFF	IAQFAPSASAFFGMS	54.80
HLA-DRB5*01:01	1	842	856	15	TSRTLSYYK	ITVATSRTLSYYKLG	54.90
HLA-DRB5*01:01	1	1173	1187	15	FHNIRGRWT	DKKQRFHNIRGRWTG	54.90
HLA-DRB5*01:01	1	1097	1111	15	FAPSASAFF	QIAQFAPSASAFFGM	59.60
HLA-DRB5*01:01	1	930	944	15	FTALTQHGK	NNTASWFTALTQHGK	60.90
HLA-DRB5*01:01	1	1064	1078	15	YNVTQAFGR	YNVTQAFGRRGPEQT	63.90
HLA-DRB5*01:01	1	1175	1189	15	FHNIRGRWT	KQRFHNIRGRWTGDY	64.50
HLA-DRB5*01:01	1	792	806	15	FARTRSMWS	FRLFARTRSMWSFNP	66.90
HLA-DRB5*01:01	1	844	858	15	TSRTLSYYK	VATSRTLSYYKLGAS	69.80
HLA-DRB5*01:01	1	840	854	15	TSRTLSYYK	KEITVATSRTLSYYK	74.10
HLA-DRB5*01:01	1	709	723	15	LAILTALRL	VTLAILTALRLCAYC	80.90
HLA-DRB5*01:01	1	1096	1110	15	FAPSASAFF	PQIAQFAPSASAFFG	84.70
HLA-DRB5*01:01	1	379	393	15	FAMQMAYRF	FAMQMAYRFNGIGVT	98.80
HLA-DRB5*01:01	1	1100	1114	15	FAPSASAFF	QFAPSASAFFGMSRI	99.50

Allele	#	Start	End	Length	Core Sequence	Peptide Sequence	IC50
HLA-DRB5*01:01	1	1176	1190	15	FHNIRGRWT	QRFHNIRGRWTGDYK	104.50
HLA-DRB5*01:01	1	710	724	15	LAILTALRL	TLAILTALRLCAYCC	115.80
HLA-DRB5*01:01	1	414	428	15	AQALNTLVK	NQNAQALNTLVKQLS	127.60
HLA-DRB5*01:01	1	681	695	15	FVSEETGTL	MYSFVSEETGTLIVN	129.60
HLA-DRB5*01:01	1	793	807	15	FARTRSMWS	RLFARTRSMWSFNPE	131.90
HLA-DRB5*01:01	1	413	427	15	AQALNTLVK	VNQNAQALNTLVKQL	153.40
HLA-DRB5*01:01	1	415	429	15	AQALNTLVK	QNAQALNTLVKQLSS	171.40
HLA-DRB5*01:01	1	1095	1109	15	FAPSASAFF	WPQIAQFAPSASAFF	179.70
HLA-DRB5*01:01	1	451	465	15	IDRLITGRL	EVQIDRLITGRLQSL	192.80
HLA-DRB5*01:01	1	682	696	15	FVSEETGTL	YSFVSEETGTLIVNS	202.10
HLA-DRB5*01:01	1	1177	1191	15	FHNIRGRWT	RFHNIRGRWTGDYKD	204.30
HLA-DRB5*01:01	1	412	426	15	AQALNTLVK	VVNQNAQALNTLVKQ	207.00
HLA-DRB5*01:01	1	452	466	15	IDRLITGRL	VQIDRLITGRLQSLQ	215.70
HLA-DRB5*01:01	1	450	464	15	IDRLITGRL	AEVQIDRLITGRLQS	242.80
HLA-DRB5*01:01	1	673	687	15	RRSMYSFVS	YRGRKRRSMYSFVSE	244.50
HLA-DRB5*01:01	1	1085	1099	15	IRQGTDYKH	LIRQGTDYKHWPQIA	250.10
HLA-DRB5*01:01	1	1129	1143	15	LNKHIDAYK	AVILLNKHIDAYKTF	252.60
HLA-DRB5*01:01	1	838	852	15	ITVATSRTL	LPKEITVATSRTLSY	265.70
HLA-DRB5*01:01	1	1101	1115	15	FAPSASAFF	FAPSASAFFGMSRIG	290.00
HLA-DRB5*01:01	1	711	725	15	LAILTALRL	LAILTALRLCAYCCN	293.60
HLA-DRB5*01:01	1	839	853	15	ITVATSRTL	PKEITVATSRTLSYY	295.70
HLA-DRB5*01:01	1	411	425	15	AQALNTLVK	DVVNQNAQALNTLVK	308.90
HLA-DRB5*01:01	1	416	430	15	AQALNTLVK	NAQALNTLVKQLSSN	311.00
HLA-DRB5*01:01	1	277	291	15	FNGLTGTGV	VNFNFNGLTGTGVLT	335.90
HLA-DRB5*01:01	1	837	851	15	ITVATSRTL	DLPKEITVATSRTLS	337.40
HLA-DRB5*01:01	1	1130	1144	15	LNKHIDAYK	VILLNKHIDAYKTFP	371.80
HLA-DRB5*01:01	1	449	463	15	IDRLITGRL	EAEVQIDRLITGRLQ	378.50
HLA-DRB5*01:01	1	794	808	15	FARTRSMWS	LFARTRSMWSFNPET	394.60
HLA-DRB5*01:01	1	453	467	15	IDRLITGRL	QIDRLITGRLQSLQT	418.80
HLA-DRB5*01:01	1	1104	1118	15	FFGMSRIGM	SASAFFGMSRIGMEV	428.80
HLA-DRB5*01:01	1	276	290	15	FNGLTGTGV	CVNFNFNGLTGTGVL	452.50
HLA-DRB5*01:01	1	1103	1117	15	ASAFFGMSR	PSASAFFGMSRIGME	490.20
HLA-DRB5*01:01	1	1131	1145	15	LNKHIDAYK	ILLNKHIDAYKTFPP	502.40
HLA-DRB5*01:01	1	618	632	15	YEQYIKWPW	ELGKYEQYIKWPWYI	513.80
HLA-DRB5*01:01	1	888	902	15	FHPLADNKF	GNSPFHPLADNKFAL	514.60
HLA-DRB5*01:01	1	409	423	15	VVNQNAQAL	LQDVVNQNAQALNTL	614.40
HLA-DRB5*01:01	1	960	974	15	FYYLGTGPE	LSPRWYFYYLGTGPE	675.40

Allele	#	Start	End	Length	Core Sequence	Peptide Sequence	IC50
HLA-DRB5*01:01	1	836	850	15	ITVATSRTL	KDLPKEITVATSRTL	679.60
HLA-DRB5*01:01	1	1132	1146	15	LNKHIDAYK	LLNKHIDAYKTFPPT	716.20
HLA-DRB5*01:01	1	635	649	15	IAGLIAIVM	WLGFIAGLIAIVMVT	731.20
HLA-DRB5*01:01	1	561	575	15	FKEELDKYF	LDSFKEELDKYFKNH	755.30
HLA-DRB5*01:01	1	1178	1192	15	FHNIRGRWT	FHNIRGRWTGDYKDD	755.70
HLA-DRB5*01:01	1	421	435	15	VKQLSSNFG	NTLVKQLSSNFGAIS	780.40
HLA-DRB5*01:01	1	420	434	15	VKQLSSNFG	LNTLVKQLSSNFGAI	786.90
HLA-DRB5*01:01	1	497	511	15	VDFCGKGYH	QSKRVDFCGKGYHLM	831.80
HLA-DRB5*01:01	1	560	574	15	FKEELDKYF	ELDSFKEELDKYFKN	847.40
HLA-DRB5*01:01	1	617	631	15	YEQYIKWPW	QELGKYEQYIKWPWY	863.10
HLA-DRB5*01:01	1	448	462	15	IDRLITGRL	VEAEVQIDRLITGRL	867.30
HLA-DRB5*01:01	1	410	424	15	VVNQNAQAL	QDVVNQNAQALNTLV	871.10
HLA-DRB5*01:01	1	259	273	15	YRVVVLSFE	GYQPYRVVVLSFELL	879.20
HLA-DRB5*01:01	1	408	422	15	VVNQNAQAL	KLQDVVNQNAQALNT	886.00
HLA-DRB5*01:01	1	489	503	15	CVLGQSKRV	KMSECVLGQSKRVDF	898.70
HLA-DRB5*01:01	1	349	363	15	FNGLTVLPP	CAQKFNGLTVLPPLL	902.90
HLA-DRB5*01:01	1	382	396	15	YRFNGIGVT	QMAYRFNGIGVTQNV	913.00
HLA-DRB5*01:01	1	562	576	15	FKEELDKYF	DSFKEELDKYFKNHT	922.30
HLA-DRB5*01:01	1	488	502	15	CVLGQSKRV	TKMSECVLGQSKRVD	948.10
HLA-DRB5*01:01	1	427	441	15	FGAISSVLN	LSSNFGAISSVLNDI	954.10
HLA-DRB5*01:01	1	1106	1120	15	FFGMSRIGM	SAFFGMSRIGMEVTP	965.10
HLA-DRB5*01:01	1	383	397	15	FNGIGVTQN	MAYRFNGIGVTQNVL	979.80
